# From ancient practice to modern innovation solving the clay mineral puzzle in brickmaking

**DOI:** 10.1038/s41467-026-78652-0

**Published:** 2026-10-14

**Authors:** Sen Wang, Lloyd Gainey, Lihui Liu, Zijun Zeng, Shusheng Xiao, Runliang Zhu, Sara Couperthwaite, Yuantong Gu, Yunfei Xi

**Affiliations:** 1 State Key Laboratory of Advanced Environmental Technology, Guangzhou Institute of Geochemistry, Chinese Academy of Sciences (CAS), Guangzhou, China; 2 School of Mechanical, Medical and Process Engineering, Faculty of Engineering, Queensland University of Technology (QUT), Brisbane, Queensland Australia; 3 Austral Bricks, Rochedale, Queensland Australia; 4 School of Environmental and Municipal Engineering, Lanzhou Jiaotong University, Gansu, China

**Keywords:** Ceramics, Mechanical properties

## Abstract

Clay minerals are the most crucial constituents in brickmaking clays, yet the specific role of different clay minerals remain poorly understood due to their natural coexistence and formidable challenges of separation. Here, we design a kaolinite-illite-quartz-feldspar system through a multiple-stage purification strategy, enabling direct evaluation of two of the most critical clay minerals for brick making - kaolinite and illite. Precise tuning the initial clay mineral assemblage yields brick strengths up to 8.5 times the ASTM C62 Grade MW requirement, without upgrading existing production methods. Mineralogical mechanisms responsible for substantial strength enhancement are revealed. Extensive analyses of 60 brick formulations uncovered a breakdown of conventional linear relationships among different brick properties due to the temperature-dependent responses of kaolinite and illite; specific formation pathways of mullite and amorphous phases; and over-firing defects above 1200 °C due to K^+^-induced fluxing from illite. Notably, a previously unrecognized negative correlation between mullite and brick strength is identified, suggesting a need to reconsider the widely held assumption of mullite as a strengthening phase. This work not only presents insights for optimizing high-strength brick formulations but also opens avenues for investigating the intricate contributions of distinct starting components to key brick performance.

## Abstract (web summary)

This study shows how the common clay minerals kaolinite and illite shape mineral transformations, pore development and strength during brick firing, offering a temperature-based guide to reduce defects and produce stronger bricks.

## Introduction

Fired bricks have been manufactured for over 6000 years^1^, yet low mechanization in traditional brickmaking significantly limited production efficiency and consistency, and the complexity of brickmaking clays, which comprises clay minerals, quartz, feldspars, carbonates, organic matter, and Fe and/or Al oxyhydroxide^2,3^, have made it difficult to understand the underlying material science of brick formation. Modern factories have adopted high-tech machinery—crusher, mixers, extruders, and temperature control systems—to ensure efficient and consistent production of a homogeneously performing product. Additionally, the commercialization of analytical tools like X-ray diffraction (XRD) and X-ray fluorescence (XRF) enabled detailed analysis of the composition and thermal behavior of clays. Tools like the SiO_2_-Al_2_O_3_-MgO and SiO_2_-Al_2_O_3_-CaO phase diagrams now allow researchers to map high-temperature mineral transformations more accurately^4^. Thus, a comprehensive understanding of the relationship between raw materials and final brick properties has only recently emerged^1,5^. However, despite these advances, defect rates in current brick production remain stubbornly high—at approximately 3–5% even in top-performing production lines^6,7^. These defects, including cracks, spalling, chips, and efflorescence, translate into substantial economic losses, given the global annual production of 1500 billion clay bricks^8^. More critically, defective bricks waste energy and natural clay resources, increase pressure on the availability of suitable virgin raw materials, and generate additional environmental pollution and CO_2_ emissions through replacement production, firing, and carbonate decomposition^9,10^.

A major source of these defects is the inherent variability in raw clays, arising from differences in parent-material mineralogy, crystallinity, and chemical composition, which govern clay mineral formation during weathering across regions and even within a single mining site^11,12^. This variability is further amplified by the gap between scientific research and industrial practice because most existing studies are based on region-specific clays subjected to varied firing regimes, with limited use of controlled experimental designs and systematic compositional gradients^13,14^. This narrow focus makes manufacturers struggle to extract practical, locally adaptable guidelines from the current body of scientific knowledge. Consequently, brick research and production still echoes ancient trial-and-error methods without innovative breakthroughs.

To date, the most pressing challenge in the brick industry is establishing a universal understanding of how different components in clay influence the final properties of bricks. However, precisely tuning these components remains challenging due to their inherent coexistence in natural clay. In addition, clay minerals warrant special attention owing to their diversity and pivotal roles like imparting essential plasticity and vitrification upon firing to densify the brick body^15^. In 1992, Dunham^5^ systematically investigated brick-making clays in the UK, classifying clay minerals into six categories and identifying two primary variations: the kaolinite/illite (Kln/Ilt) ratio and the quartz/total clay mineral ratio. These ratios are particularly significant because Kln and Ilt are considered the two most common clay minerals in ceramic clays^16^, and their proportional variations have been widely documented from brickmaking in Spain^17^, Italy^18^, Australia^19^, Czechia^20^, and Brazil^21^.

Despite the recognized importance and wide compositional variability of Kln and Ilt, there remains a surprising lack of studies directly linking them to critical brick properties. Limited research has explored their influence on mullite and cristobalite formation^22^, and the contributions of Ilt-rich clay to brick bulk density and Young’s modulus^16^. To date, no definitive conclusions have been reached regarding how Kln and Ilt affect the thermal behavior of clay mixes and key technological performance of final bricks. This lack of fundamental insights not only significantly limits innovation in the brick industry but also increases the risk of producing defective bricks due to mismatched raw materials and processing conditions.

This study aims to complete a critical piece of the brick manufacturing puzzle by systematically revealing the roles of the clay minerals Kln and Ilt in brick production. We designed a Kln-Ilt-Quartz-Feldspar (KIQF) system that allows flexible tailoring in types, proportions, and characteristics of clay mineral in brick raw materials. Gradient variations of Kln and Ilt were obtained using KIQF, and a total of 60 different brick formulations were prepared across a wide firing temperature range. The impacts of Kln and Ilt on phase composition, microstructure, color, and the physical and mechanical properties of fired bricks were comprehensively assessed. Specifically, optimal clay mix formulations at different firing temperatures were proposed, and the underlying mineralogical mechanisms were elucidated. Quantitative correlation analyses presented insights into the relationships among phase compositions, porosity, and strength of fired bricks. These findings offer practical guidance for improving product consistency and mechanical performance in industrial brick manufacturing.

## Results

### Design of KIQF system

Precisely controlling the relative proportions of Kln and Ilt through mineral separation and purification is a critical first step in understanding their individual roles in brickmaking, but this remains a major challenge and has rarely been reported^19^. This difficulty stems from the common coexistence of clay minerals, which exhibit similar particle sizes and overlapping structural characteristics, making their separation and independent manipulation extremely difficult^11,23,24^. In addition, because clay minerals commonly form through the weathering of different parent rocks and coexist with other mineral phases, exceptionally pure natural clay minerals are rare^23,25^.

To address this challenge, we designed a KIQF system composed of three adjustable ingredients: heavy particles (Hp), Kln, and Ilt, through a multi-stage purification strategy. KIQF enables precise control of different clay minerals, providing a design concept that enables systematic differentiation of their individual contributions. Hp fraction was derived from industrial brick-making clay in Queensland, Australia, with its clay mineral components removed through purification (details provided in Supplementary Note 1). Hp represents the non-clay mineral components typically present in brick raw materials. In addition, Kln and Ilt were isolated from natural clay sources and further purified in the laboratory, as described in Supplementary Note 2. The effectiveness of the purification was evaluated by comparing XRD patterns from the powder and clay slides before and after treatment (Supplementary Figs. 1 and 2). Rietveld refinement demonstrates that the purity of Kln and Ilt increased from 76.68% and 45.5% to 93.54% and 93.85%, respectively, while Hp consists of quartz (65.81%), feldspar (2.57%), hematite, goethite, and minor clay minerals (Supplementary Fig. 3).

To further characterize the raw materials, their morphologies and particle size distributions were measured. Scanning electron microscopy (SEM) images reveal that both Kln and Ilt exhibit layered stacking, with Ilt showing an irregular plate-like morphology and Kln presenting a pseudohexagonal shape (Supplementary Fig. 4). Laser particle size analyses show that Kln and Ilt exhibit comparable particle size ranges, although Kln is slightly finer than Ilt (*D*_50_ = 1.28 and 3.94 μm; *D*_90_ = 5.39 and 10.2 μm for Kln and Ilt, respectively, Supplementary Fig. 5, Supplementary Table 1). Hp exhibits a broader bimodal distribution, with a *D*_50_ of 11.5 μm and a much larger *D*_90_ of 1760 μm due to the presence of coarse particles.

A mixture of Hp, Kln, and Ilt was used to construct the KIQF system as a representative brickmaking raw material. To comprehensively capture the variability of Kln and Ilt contents encountered in practical brick production and fully elucidate their roles, a broad compositional and temperature space was systematically established. Three independent variables were set: (1) total clay mineral content in KIQF, calculated as (Kln+Ilt)/(Kln+Ilt+Hp), at 20%, 35%, and 50%, where Kln+Ilt+Hp represents the complete KIQF raw-material system; (2) Kln-to-clay mineral ratio, calculated as Kln/(Kln+Ilt), at 0, 33.3%, 50%, 66.6%, and 100%; and (3) firing temperatures at 900, 1050, 1200, and 1350 °C. The Ilt-to-clay mineral ratio was not separately included as an independent variable because it is mathematically complementary to the Kln-to-clay mineral ratio (i.e., Ilt/(Kln+Ilt) = 1-Kln/(Kln+Ilt)). It is important to note that the variability within and between geological formations of brick clays primarily arises from differences in Kln/Ilt ratio and the proportion of clay minerals relative to non-clay heavy particles (mainly quartz)^5^. In the KIQF system, these two compositional variations are represented by Kln/(Kln+Ilt) and (Kln+Ilt)/(Kln+Ilt+Hp), respectively, enabling their individual effects on fired brick properties to be systematically evaluated. In addition, the wide temperature range was selected to span both low-temperature energy efficiency and high-temperature strength optimization practices typically used in industrial brick firing. Table 1 summarizes the 15 clay mixes prepared. With each composition subjected to four firing temperatures, a total of 60 brick formulations were generated. To the best of our knowledge, such a comprehensive and systematic exploration of raw material variations has not yet been reported in existing literature.

**Table 1:** Formulations of clay mixes

Clay mix ID	(Kln+Ilt)/(Kln+Ilt+Hp) (%)	Kln/(Kln+Ilt) (%)
20/0	20	0
20/33	20	33
20/50	20	50
20/66	20	66
20/100	20	100
35/0	35	0
35/33	35	33
35/50	35	50
35/66	35	66
35/100	35	100
50/0	50	0
50/33	50	33
50/50	50	50
50/66	50	66
50/100	50	100

### Thermal expansion and shrinkage of clay mixes

**Fig. 1:**
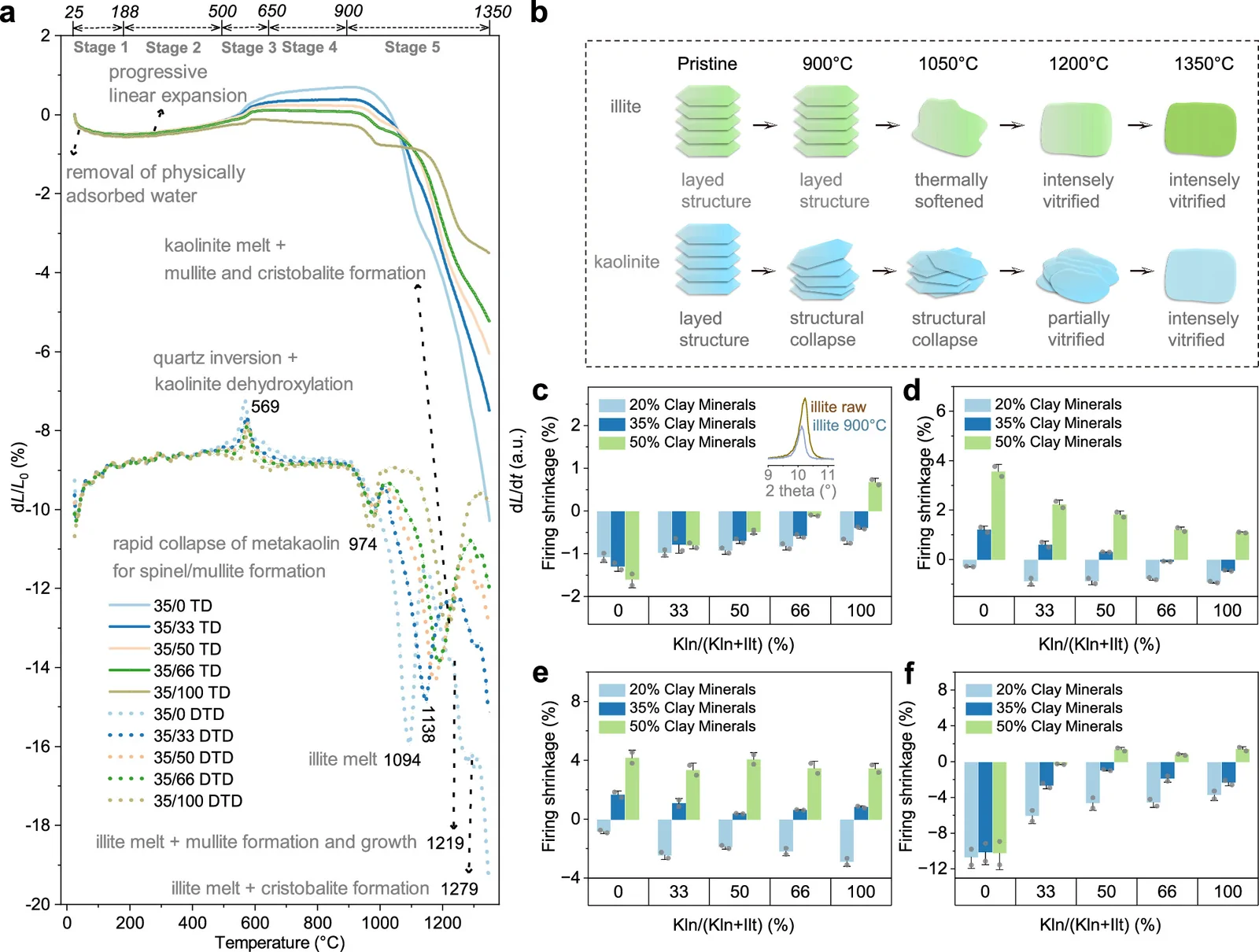
Volume changes in Kln, Ilt and clay formulations during heating. **a** Thermal dilatometry curves and their first derivatives (d*L*/d*t*) for the 35/0, 35/33, 35/50, 35/66, and 35/100 clay mixtures. **b** Schematic illustration of the morphological evolution of Kln and Ilt upon heating. **c**–**f** Firing shrinkage of the clay mixtures as a function of the Kln/(Kln + Ilt) ratio at **c** 900 °C, **d** 1050 °C, **e** 1200 °C, and **f** 1350 °C. Data in (**c**–**f**) are presented as the mean of two independent measurements (*n*  =  2), with gray circles indicating the individual measurement results. Error bars represent the sample standard deviation (SD), calculated by dividing the sum of squared deviations from the mean by *n* − 1 and taking the square root. The inset in **c** compares the (001) XRD reflections of raw Ilt and Ilt fired at 900 °C. Kln, kaolinite; Ilt, illite. Source data are provided as a Source Data file.

Prior to examining the properties of fired bricks, thermal shrinkage/expansion of clay mixtures were investigated, as these processes dictate porosity evolution and sintering—both significantly affecting the final physical and mechanical performance of bricks. Across 25–1350 °C, thermal shrinkage/expansion can be divided into five distinct stages (Fig. 1a and Supplementary Fig. 6)^15^. Notably, at a fixed total clay mineral content, variations in the Kln/(Kln + Ilt) ratio only affect the final three stages (above 500 °C).

In stage 3, brick expansion increases sharply due to the α-to-β quartz inversion near 573 °C (Fig. 1a). This behavior is partially counteracted by dehydroxylation of Kln, resulting in structural collapse and increases with the Kln/(Kln+Ilt) ratio^4,26^. In-situ high-temperature XRD results presented in Section “Physical and Mechanical Properties of Fired Bricks” indicates that Kln dehydroxylates by 550 °C, allowing the associated shrinkage to overlap with stage 3. Thus, greater incorporation of Kln may reduce thermally induced cracking in clay bodies. In contrast, Ilt shows no comparable collapse-related shrinkage within this temperature range. Although Ilt dehydroxylates between 600 and 700 °C (as will be discussed based on the in-situ XRD result in Section “Physical and Mechanical Properties of Fired Bricks”), its layered structure remains largely preserved. It is reported that Ilt continuously expands upon firing below 1000 °C^27^, aligning with the expansion observed in the 35/0 sample below 900 °C (Fig. 1a). Because the dehydroxylation temperatures of Kln and Ilt vary with chemistry and crystallinity, and additionally with octahedral-sheet occupancy for Ilt, this interpretation is specific to the present samples.

In stage 4 (650–900 °C), replacing Ilt with Kln shifts the clay mix from expansion (0.21% for 35/0) to shrinkage (−0.12% for 35/100) (Fig. 1a), attributed to progressive structural rearrangement of metakaolin prior to the formation of high-temperature crystalline phases^28^.

In stage 5 (900–1350 °C), the firing shrinkage decreases from 10.98% (35/0) to 7.87% (35/33), 6.27% (35/50), 5.30% (35/66), and 3.24% (35/100) with increasing Kln/(Kln+Ilt) ratio. This indicates that compared to Kln, Ilt in the clay mix leads to a higher degree of sintering, likely due to the fluxing action of K^+^ ions in its interlayer space^29,30^. XRF test in Supplementary Table 2 confirmed that Ilt, with a combined K, Na, Mg, and Ca oxide content of 10.79%, is a high-fluxing material, while Kln and Hp exhibit much lower levels of fluxing agents, at 0.64% and 2.49%, respectively. In addition, under SEM observation, pure Ilt vitrifies intensely at 1200 °C, indicating substantial viscous-phase formation, while Kln still partially reserves its morphology (Fig. 1b, Supplementary Fig. 7), further demonstrating the higher vitrification capacity of Ilt than Kln.

Apart from the thermal dilatometry, the dimensional change of brick buttons after firing were also examined. Green bodies show an expanding trend after 900 °C (Fig. 1c), with the expansion value decreasing as the Kln/(Kln+Ilt) ratio increases. At 1050 and 1200 °C, higher Kln/(Kln+Ilt) ratios result in reduced firing shrinkage due to the lower vitrification capacity of Kln compared to Ilt (Fig. 1d, e). Interestingly, bricks made from Ilt and Hp exhibit approximately 10% expansion at 1350 °C instead of shrinkage (Fig. 1f), attributed to the so-called “overfiring”^31^. During firing, open pores typically close as the brick densifies. However, beyond maximum densification, rising gas pressure in fully closed pores can lead to expansion as the temperature continues to rise. This mechanism is supported by micro-CT scans of the 50/0 specimen, where open porosity decreases from 9.85% to 1.55% while closed porosity increases from 0.12% to 3.40% as firing temperature increases from 900 to 1350°C (Supplementary Fig. 8). Introducing Kln to the clay mix can mitigate this effect, due to the superior refractory properties of Kln compared to Ilt (Fig. 1b).

In addition, higher total clay mineral content leads to greater firing shrinkage across most formulations (Fig. 1c–f), consistent with the thermal dilatometry results in Supplementary Fig. 6. An exception occurs when Kln/(Kln + Ilt) = 0 and the firing temperature is 900 °C, where increased Ilt content results in expansion rather than shrinkage (Fig. 1a). The d_(001)_ spacing of Ilt increases by only approximately 2% after firing at 900 °C (inset in Fig. 1c), possibly due to dehydroxylation-induced Al redistribution within the octahedral sheet^32,33^. Assuming complete transfer of this crystallographic strain and neglecting the random orientation of Ilt particles, the estimated contributions to bulk expansion are 0.4%, 0.7%, and 1.0% for formulations containing 20%, 35%, and 50% Ilt, respectively. These values are lower than the corresponding measured expansions in Fig. 1c, indicating that Ilt lattice expansion alone cannot fully account for the observed behavior. Because increasing the Ilt content simultaneously decreases the Hp proportion, associated changes in the overall particle-size distribution, particle packing, and pore structure may also contribute.

### Physical and mechanical properties of fired bricks

#### Microstructure and appearance

**Fig. 2:**
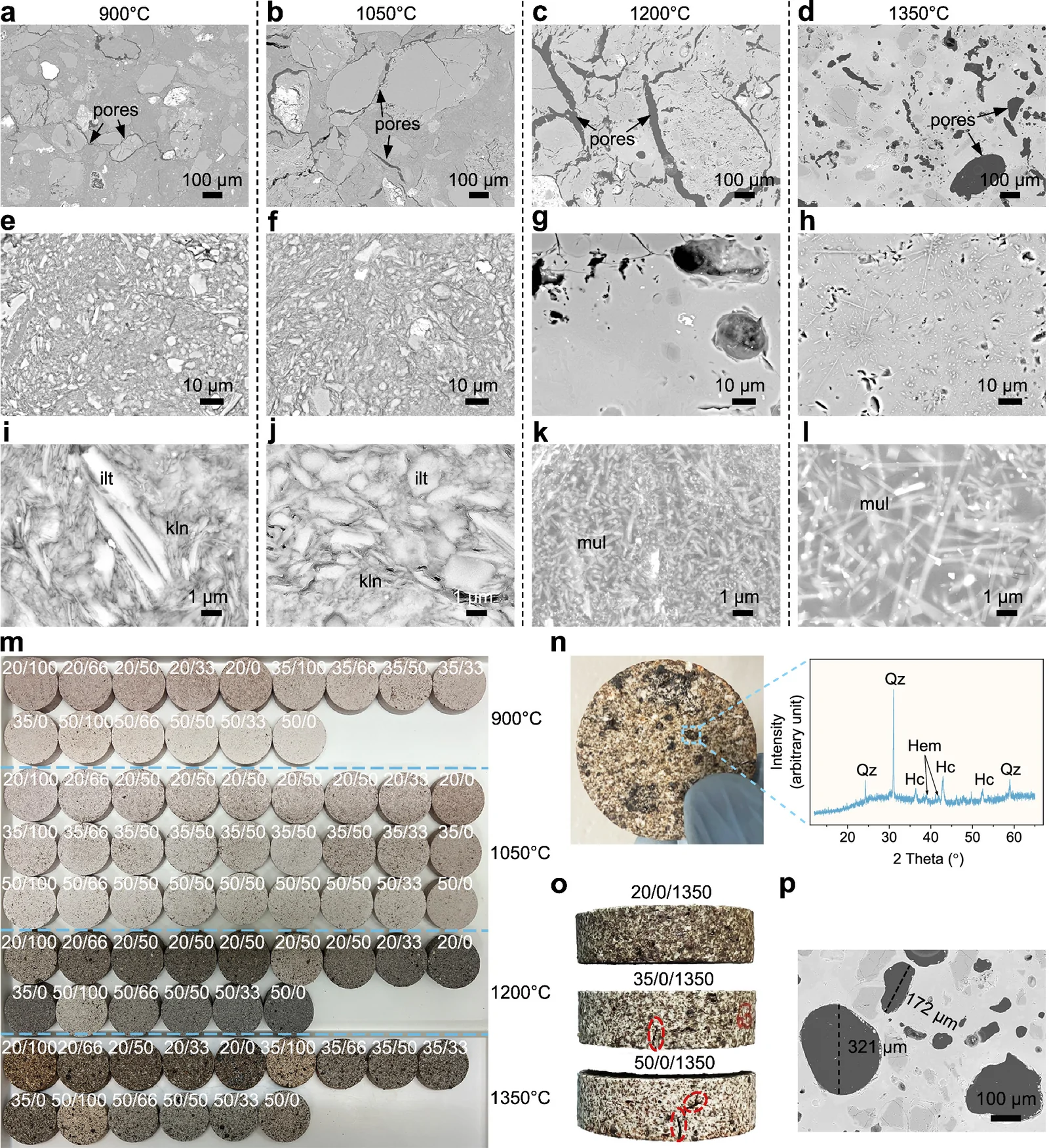
Microscopic and macroscopic morphological features of different fired formulations. Pore evolution in the 35/50 formulation fired at **a** 900 °C, **b** 1050 °C, **c** 1200 °C and **d** 1350 °C. Microstructural evolution of the clay mineral matrix in the 35/50 formulation fired at **e**, **i** 900 °C, **f**, **j** 1050 °C, **g**, **k** 1200 °C, and **h**, **l** 1350 °C. For each firing temperature, the paired images show the microstructure at different magnifications. **m** Photographs of 72 fired brick buttons. Dashed lines separate the brick buttons with firing temperatures. **n** Black spots in the 35/0 formulation fired at 1200 °C and the corresponding X-Ray diffraction pattern. The rectangular dashed box marks the black spot. **o** Crack development in the 20/0, 35/0 and 50/0 formulations fired at 1350 °C. Red circles indicate the cracks. **p** Closed, rounded pores in the 50/0 formulation fired at 1350 °C. Dashed lines indicate the pore diameters. Kln kaolinite, Ilt illite, Mul mullite, Qz quartz, Hem hematite, Hc hercynite. Source data are provided as a Source Data file.

SEM on fine polished bricks effectively reveals pore distribution and phase interaction, providing critical insights into the physical and mechanical properties. At 35% clay minerals and 1350 °C, decreasing the Kln/(Kln+Ilt) ratio results in (1) reduced pores and their connectivity, (2) transition from elongated to rounded-shape pores, and (3) a loss of small pores (Supplementary Fig. 9). These changes are primarily attributed to the superior vitrification behavior of Ilt compared to Kln, which enhances pore coalescence and elimination, as well as grain consolidation. Moreover, a distinct evolution of pore characteristics is observed with increasing temperature (Fig. 2a–d) –900 °C pores from loose particle packing; 1050 °C and 1200 °C elongated pores ( > 100 µm) from differential shrinkage of Kln, Ilt, and quartz particles; 1350 °C closed and rounded pores from substantial liquid-phase formation—reflecting the progressive sintering and densification of the brick structure^34,35^.

In addition to pore evolution, EDS mapping reveals the contact relationships between different phases (Supplementary Fig. 10). The evolution of the clay mineral matrix was also observed with firing temperature. At 900 and 1050 °C, the matrix comprises loosely packed Kln and Ilt with clearly defined phase boundaries (Fig. 2e, f, i, j). EDS analysis confirms that Kln is Al-rich, while Ilt contains higher concentrations of K, Fe, and Mg (Supplementary Fig. 11). Both phases retain their layered morphology, indicating limited vitrification at 900 °C. At 1200 °C, a partially vitrified matrix forms (Fig. 2c), incorporating fine, poorly crystallized mullite (<2 µm) (Fig. 2g, k). Some residual lamellar structures remain, with an Al: Si ratio of ~1: 1, suggesting they are unreacted Kln (Supplementary Fig. 12). Upon firing to 1350 °C, the microstructure transitions to a highly vitrified and homogeneous glassy matrix (Fig. 2d), accompanied by pore closure and reduction in size (Fig. 2h). Well-developed needle-shaped mullite crystals emerge, some exceeding 10 µm (Fig. 2l). EDS analysis shows an Al: Si ratio of ~3:2 in these areas (Supplementary Fig. 13), indicative of secondary mullite formation promoted by the presence of alkali fluxes^36^.

In terms of appearance, dried bricks shift their colors from oyster to neutral gray, becoming lighter at higher clay mineral contents (Supplementary Fig. 14). For fired bricks, color deepens with temperature, ranging from white at 900 °C to mushroom pink at 1050 °C, cloud gray at 1200 °C, and dark brown at 1350 °C (Fig. 2m). This darkening is due to the increased crystal size of hematite with temperature (Supplementary Fig. 15)^19^. In addition, higher clay mineral content results in lighter colors, attributed to the lower Fe content in Kln and Ilt compared to Hp. Decreasing the Kln/(Kln+Ilt) ratio also deepens the brick color, possibly because the higher fluidity of the Ilt-based glass matrix facilitates the growth of larger hematite crystals.

Interestingly, surface defects such as black spots and cracks emerge on Hp-Ilt bricks fired at 1200 °C or higher (Fig. 2n,o). XRD analysis reveals that the black spot consists of hercynite (FeAl_2_O_4_), hematite (Fe_2_O_3_), and quartz. According to Laita, Bauluz^37^, hercynite can evolve from Fe particles at high temperatures, partially replacing hematite crystals. In this experiment, the hercynite-rich exudate increases with both Ilt and Hp content. Their presence is due to the Fe-Al interaction in the low-viscosity glassy matrix resulting from intensive vitrification of Ilt. In addition, cracks gradually develop with increasing Ilt content in the starting clay (Fig. 2o), likely due to enhanced liquid-phase sintering, which produces large, closed pores that expand during heating and generate internal stress within the brick (Fig. 2p).

#### Phase composition and in-situ high-temperature XRD

**Fig. 3:**
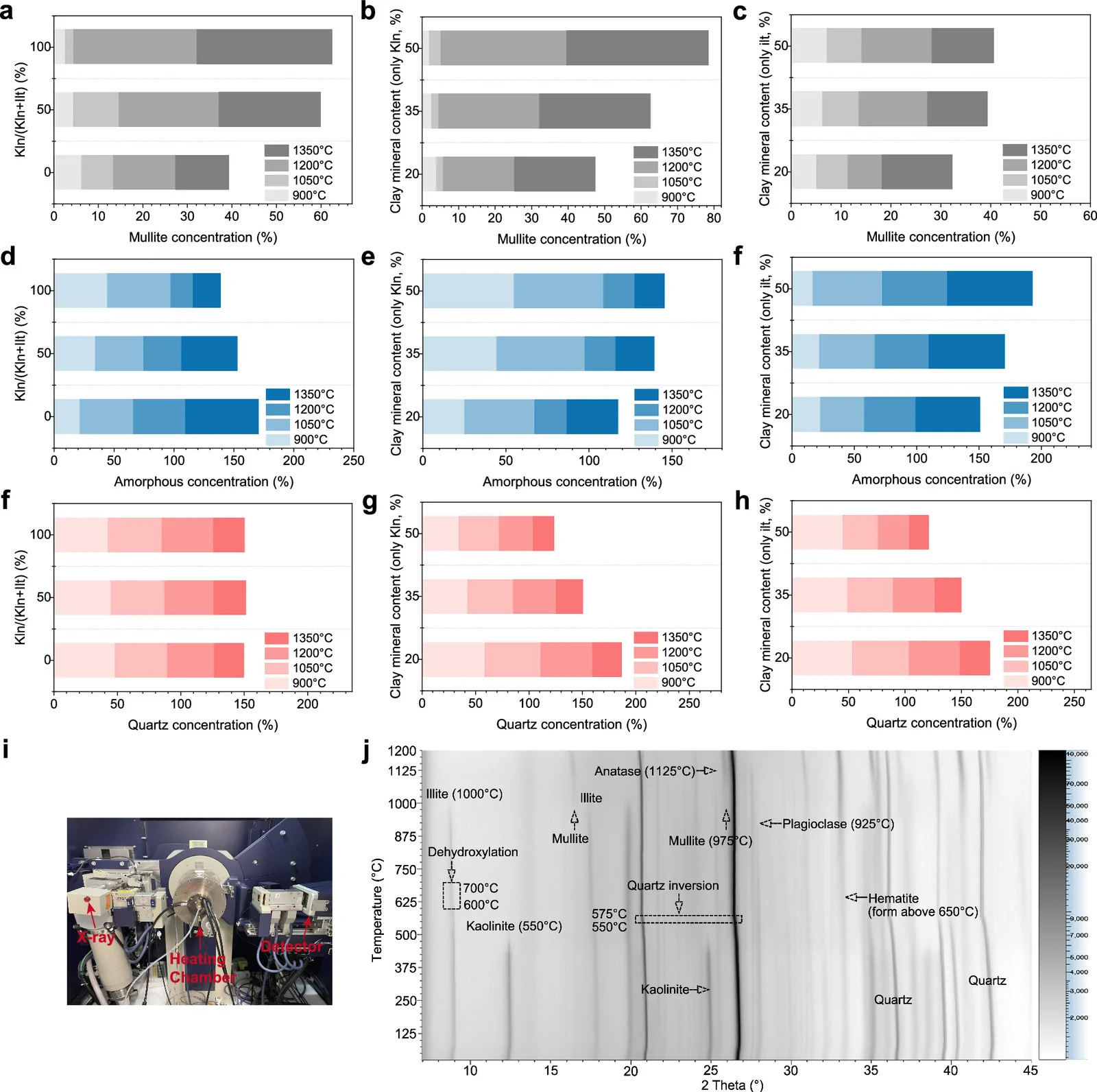
Quantitative phase analysis and in-situ high-temperature XRD patterns of clay formulations. Quantitative contents of **a**–**c** mullite, **d**–**f** the amorphous phase, and **g**–**i** quartz. The phase contents are plotted against the Kln/(Kln + Ilt) ratio at a fixed total clay content of 35% in **a**, **d**, **g**, and against the total clay content when **b**, **e**, **h** Kln is the sole clay mineral or when **c**, **f**, **i** Ilt is the sole clay mineral. **j** In-situ XRD experimental setup. **k** Two-dimensional in-situ XRD patterns of the 35/50 formulation collected from 25 to 1200 °C, with the characteristic reflections of Kln, Ilt, hematite, mullite, plagioclase and anatase labeled. Kln kaolinite, Ilt illite. Source data are provided as a Source Data file.

As a critical factor in determining brick performance, a quantitative investigation of phase composition is essential. In the absence of Ca and Mg, the primary phases in bricks are quartz, mullite, and amorphous phases^38,39^. Quantitative XRD analyses of these three phases were performed through Rietveld refinement (Supplementary Figs. 16–25), with results presented in Fig. 3a–i. In addition, in-situ high-temperature XRD was employed to reveal the real-time phase transitions of clay mix fired up to 1200 °C (Fig. 3j, k).

Mullite Formation: Mullite forms through the decomposition of clay minerals, with a formation temperature at 975 °C (Fig. 3k). The crystal size and morphology of mullite depend strongly on firing temperature and the starting aluminosilicate phases, with the effects further modified by mineral impurities^40^. Previous study suggest that fluxing agents like K and Na enhance the fluidity of Si-rich amorphous phases, promoting greater mullite crystal growth^41^. In addition, Fe often lowers the surface energy along specific crystallographic directions, favoring the development of elongated, acicular mullite grains^42^. Structural Fe can participate in the structural reorganization associated with mullite nucleation and enter the mullite structure^43^; consequently, Fe-enriched Kln may form primary mullite at lower temperatures. Because the Kln and Ilt examined here are relatively Fe-poor, less-weathered clays with higher Fe contents may exhibit different mullite formation temperatures, contents, and morphologies. In the current KIQF system, mullite formation is a complex process influenced by fluxing agents, the Al/Si ratio of clay minerals, and firing temperature:

1. At 900 °C: Mullite is primarily observed in Ilt-rich samples (Fig. 3a). The formation of mullite at such a low temperature is largely attributed to K^+^ and other alkali/alkaline-earth cations located in or near the holes of the hexagonal silica tetrahedral layers^44^. In contrast, Kln in the starting material promotes the formation of Al-rich or Al-Si spinel-type intermediates prior to substantial mullite crystallization, as confirmed by previous TEM observations^41^.
2. At 1050 °C: Maximum mullite content (10.19%) occurs when Kln and Ilt coexist (Fig. 3a). Compared to Ilt with an Al: Si of 1: 2, the Al: Si ratio of Kln (1: 1) is closer to that of mullite (3: 2 or 2: 1), promoting mullite crystallization. However, the highly viscous Si-rich amorphous phase from Kln decomposition simultaneously limits mullite growth. As for Ilt, although the highly fluid amorphous phase favors mullite formation, its lower Al: Si ratio (2: 1) hinders it. Therefore, a combination of Kln and Ilt yields the highest mullite content (10.19%) at 1050 °C. This complementary mechanism may account for the positive correlations previously reported between both Kln and Ilt contents and mullite formation^45^. Moreover, the effect of fluxing agents appears more significant than the Al: Si ratio, as evidenced by the lower mullite in the 35/100/1050 brick (1.92%) compared to the 35/0/1050 brick (7.22%) (Fig. 3a).
3. At 1200 °C and 1350 °C: Both Kln and Ilt vitrify extensively (Fig. 1b, Supplementary Fig. 7), and mullite formation is governed mainly by the overall Al: Si availability in the melt rather than by differences in fluxing action. As Kln provides a higher Al contribution than Ilt, increasing Kln shifts the system toward a more favorable Al: Si ratio for mullite, leading to a maximum mullite of 38.77% for 50/100/1350 (Fig. 3a).

Additionally, while mullite forms from the decomposition of clay minerals, a higher total clay mineral content in the starting clay does not always guarantee more mullite in the final bricks: when Kln is the dominant clay mineral, mullite content increases with clay mineral content above 1200 °C (Fig. 3b); in contrast, when Ilt is the primary clay mineral, this increase occurs at a lower temperature of 900 °C (Fig. 3c).

Amorphous Phase: Amorphous phase evolution inversely correlates with mullite because both phases derive from decomposed clay minerals (Fig. 3d)—amorphous phase comes from Si-rich components, while mullite forms from Al-rich components. Thus, the changes in amorphous content with both Kln/(Kln+Ilt) and clay mineral content are opposite to those of mullite (Fig. 3d–f). Note that the decrease in amorphous content above 1200 °C may also result from cristobalite formation from amorphous Si. In summary, both the formation of mullite and amorphous are significantly affected by initial Kln/(Kln+Ilt) and clay mineral content, highly dependent on firing temperature.

Quartz: Unlike mullite and amorphous phases, quartz content remains relatively stable across varying Kln/(Kln+Ilt) ratios (Fig. 3g, i). A specific interpretation on quartz variation among different formulations can be seen in Supplementary Note 3.

#### Water absorption, apparent porosity, and bulk density

**Fig. 4:**
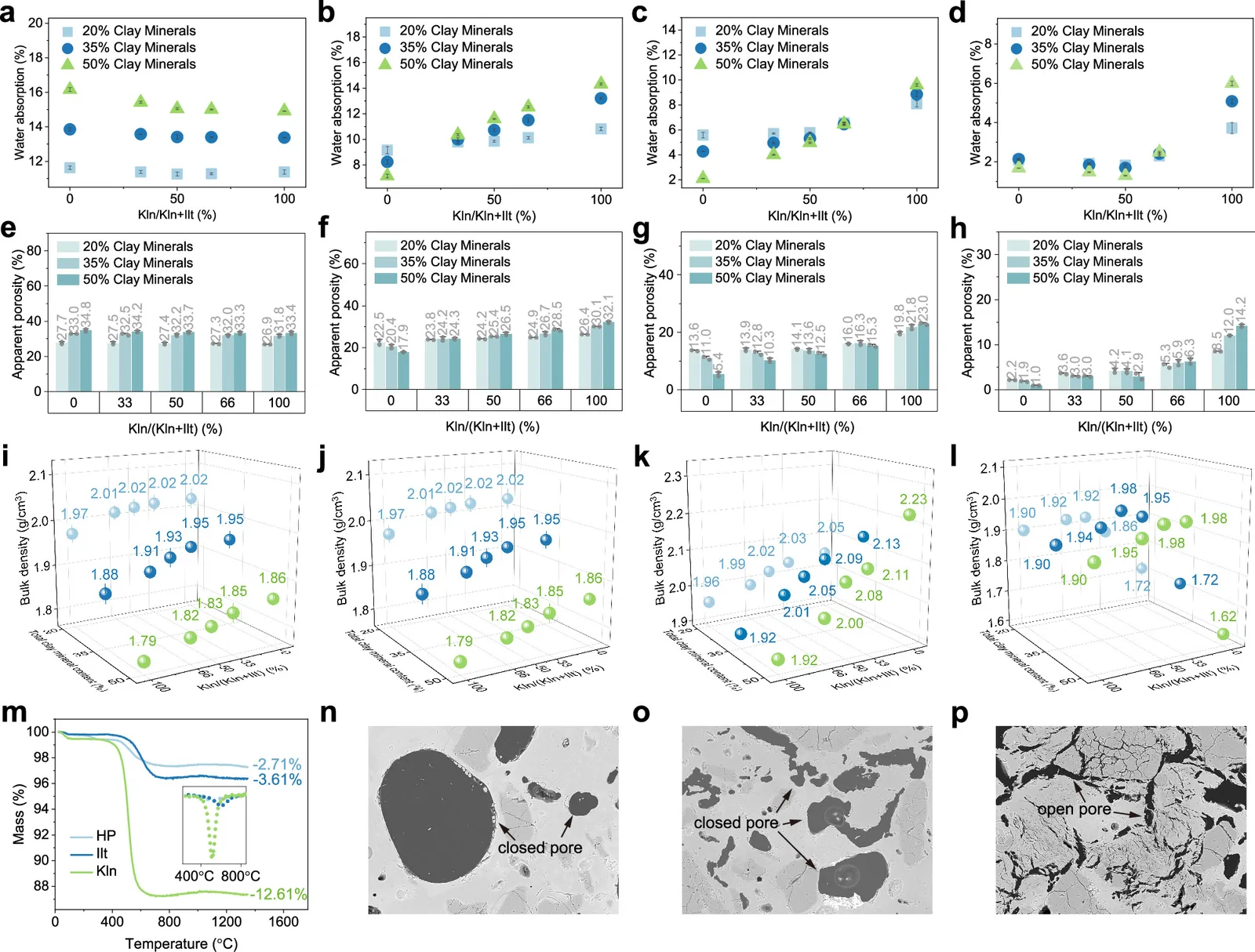
Physical properties, thermal behavior and pore evolution of different fired clay formulations. Water absorption of bricks fired at **a** 900 °C, **b** 1050 °C, **c** 1200 °C and **d** 1350 °C as a function of the Kln/(Kln + Ilt) ratio and total clay content. Apparent porosity of bricks fired at **e** 900 °C, **f** 1050 °C, **g** 1200 °C and **h** 1350 °C. Bulk density of bricks fired at **i** 900 °C, **j** 1050 °C, **k** 1200 °C and **l** 1350 °C. Cyan, blue, and green represent total clay contents of 20%, 35% and 50%, respectively. Data in **a**–**l** are presented as the mean of two independent measurements (*n*  =  2), with gray circles indicating the individual measurements. Error bars represent the sample standard deviation (SD), calculated by dividing the sum of squared deviations from the mean by *n* − 1 and taking the square root. **m** Thermogravimetric analysis of heavy particle (Hp), Ilt, and Kln. The inset in **m** shows the corresponding derivative thermogravimetry curves of Ilt (blue) and Kln (green). Micrographs showing the reduction in large closed pores from **n** 35/0/1350 to **o** 35/50/1350 and **p** 35/100/1350. Kln, kaolinite; Ilt, illite. Source data are provided as a Source Data file.

Water absorption (WA), apparent porosity (AP), and bulk density are pore-structure-related parameters that significantly influence the structural integrity, durability, and overall performance of the obtained brick^46^. The effects of Kln/(Kln+Ilt) on WA, AP, and bulk density are strongly interdependent with temperature, as indicated by the crossovers among the compositional trends in Fig. 4a–l. Because WA and AP both reflect the volume fraction of open, connected pores, they display consistent trends with changes in starting composition and firing temperature (Fig. 4a–h). At 900 °C, the increase of Kln/(Kln+Ilt) slightly reduces WA and AP (Fig. 4a, e), primarily due to the higher firing shrinkage of Kln compared to Ilt (Fig. 1a); however, the overall differences are relatively minor. At 1050 and 1200 °C, a higher Kln proportion leads to increased WA (Fig. 4b, c), as the weaker vitrification of Kln relative to Ilt limits pore closure and retains more interconnected porosity (Fig. 4f, g). At 1350 °C, the 50/50 formulation achieves the lowest WA value (1.30%, Fig. 4d), indicating superior sintering performance. Notably, the minimum AP (1.0%) occurs for the 50/0/1350, which is attributed to both enhanced closure of open pores and expansion of the brick body. In addition, for a given formulation, AP decreases with increasing firing temperature, confirming progressive densification driven by intensified vitrification of the clay minerals. The detailed variations of WA and AP with total clay mineral content are discussed in Supplementary Note 4.

Regarding the bulk density, the obtained value varies from 1.62 to 2.23 g cm^−3^ (Fig. 4i–l) across 60 formulations. At 900 °C, bulk density decreases as the Kln/(Kln+Ilt) ratio increases, ascribed to the higher thermal mass loss of Kln compared to Ilt (Fig. 4m). The reduction in bulk density at 1050 and 1200 °C is attributed to both a lower mass and an increased dimension of the brick (see the reduced firing shrinkage with Kln/(Kln+Ilt) in Fig. 1d, e). At 1350 °C, bulk density first increases with the Kln/(Kln+Ilt) ratio due to a reduced amount of large, closed pores (Fig. 4m, p and Supplementary Fig. 9), then declines after Kln/(Kln+Ilt) exceeds 50% as brick mass decreases and firing shrinkage remains stable (Fig. 1f). In addition, the bulk density of bricks correlates closely with total clay mineral content. An increase in the clay mineral content leads to lower bulk density at 900 °C regardless of the Kln-to-Ilt ratio (Fig. 4i). This is due to the higher thermal mass loss of both Kln and Ilt compared to Hp as shown in the thermogravimetric analysis (Fig. 4m). At 1050 and 1200 °C, Ilt-rich clays tend to increase bulk density, resulting from greater firing shrinkage, while Kln-rich clays lead to a lower density with clay mineral content because of higher mass loss and reduced firing shrinkage (Fig. 4j,k). At 1350 °C, the considerably low bulk density for formulations with Kln/(Kln+Ilt) = 0 is attributed to the expansion of the brick body by the formation of large, closed pores (Fig. 4l).

#### Compressive strength

**Fig. 5:**
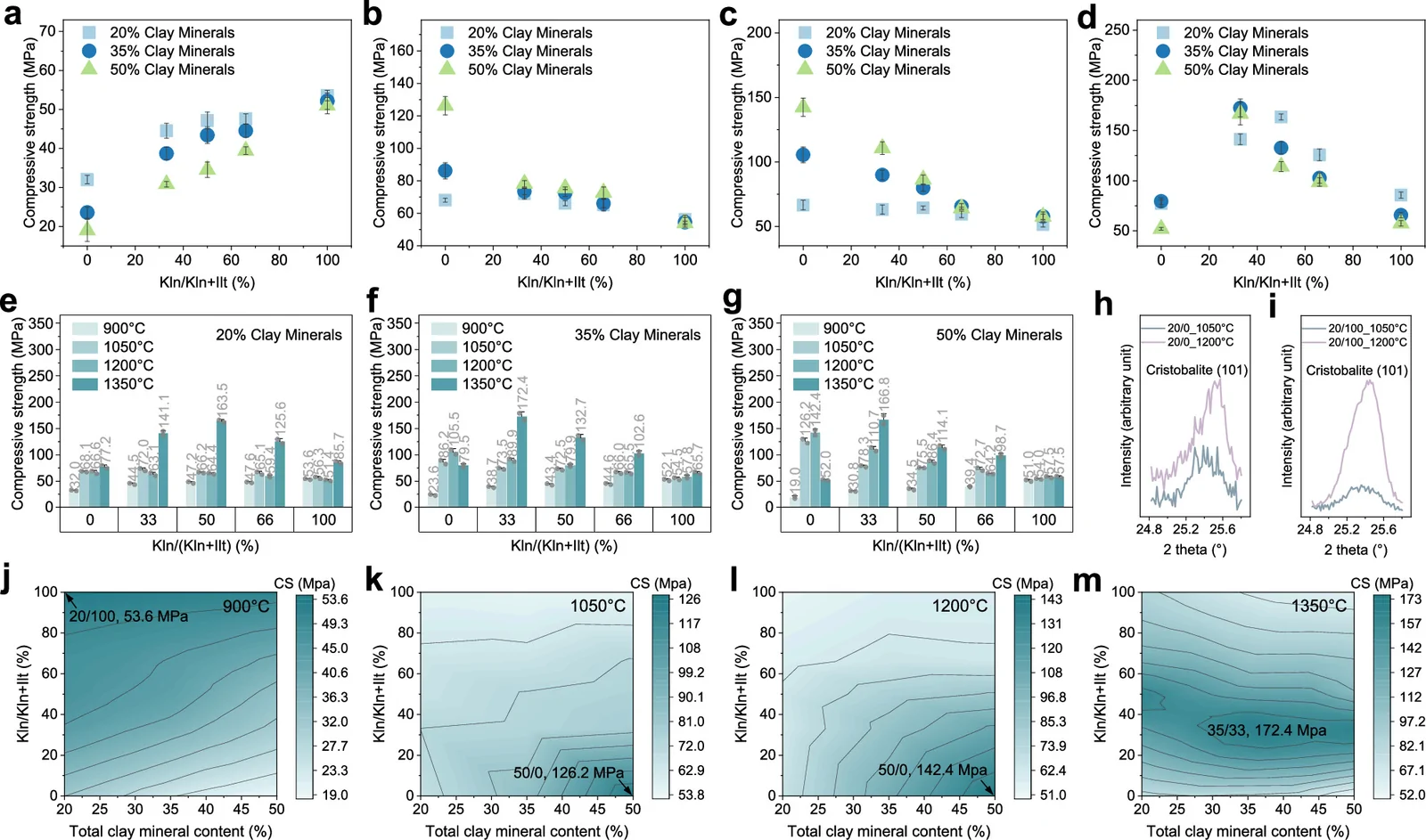
Compressive strength (CS) and cristobalite formation in different fired clay formulations. CS of bricks fired at **a** 900 °C, **b** 1050 °C, **c** 1200 °C, and **d** 1350 °C as a function of the Kln/(Kln + Ilt) ratio. Evolution of CS with firing temperature at total clay contents of **e** 20%, **f** 35% and **g** 50%. Data in **a**–**g** are presented as the mean of two independent measurements (*n*  =  2), with gray circles indicating the individual measurements. Error bars represent the sample standard deviation (SD), calculated by dividing the sum of squared deviations from the mean by *n* − 1 and taking the square root. XRD patterns showing cristobalite in the **h** 20/0 and **i** 20/100 formulations fired at 1050 °C and 1200 °C. Contour plots of CS as a function of total clay content and the Kln/(Kln + Ilt) ratio at **j** 900 °C, **k** 1050 °C, **l** 1200 °C, and **m** 1350 °C. Kln kaolinite, Ilt illite. Source data are provided as a Source Data file.

Compressive strength (CS) is crucial as fired bricks are primarily designed to withstand significant compressive loads^4^. The relationship between CS and the Kln/(Kln+Ilt) ratio varies across 900–1350 °C. At 900 °C, there is a positive correlation between CS and Kln/(Kln+Ilt) (Fig. 5a). Higher Kln content results in greater firing shrinkage due to the structural collapse and reorganization of Kln after dehydroxylation (Fig. 1a), which lowers AP (Fig. 4e) and thereby increases CS. In contrast, Ilt expands after dehydroxylation, producing porosity and reducing CS^4^. At 1050 and 1200 °C, a negative correlation between CS and Kln/(Kln+Ilt) is observed (Fig. 5b, c). This is due to the higher degree of vitrification of Ilt than Kln, which enhances brick densification, reduces AP (Fig. 4f, g), and enhances CS. At 1350 °C, the relationship between CS and Kln/(Kln+Ilt) becomes more complex (Fig. 5d). A combination of Kln and Ilt is required for optimal CS. Generally, Ilt promotes liquid sintering, reducing AP and strengthening particle-bonding, but excessive Ilt leads to cracks and the formation of large pores (Figs. 2o and 4j). As for Kln, while it increases mullite formation, its low level of vitrification weakens the liquid sintering and elevates residual AP (Figs. 1b and 4h). Consequently, the highest CS at 1350 °C is achieved in formulations where Ilt and Kln coexist, i.e., 20/50 (163.5 MPa), 35/33 (172.4 MPa), and 55/33 (166.8 MPa).

Regarding the effect of total clay mineral content: Higher clay mineral content leads to lower CS at 900 °C. This is due to the looser packing of fine clay mineral particles compared with Hp, which increases the initial porosity (Fig. 2i). At 1050 and 1200 °C, increasing clay mineral content enhances CS mainly in Ilt-rich formulations, where the greater fluxing capacity of Ilt promotes vitrification and densifies bricks (Fig. 5b, c). However, at 1350 °C, formulations with Kln/(Kln+Ilt) ratios ≥50% exhibit reduced CS when clay mineral content increases (Fig. 5d). This is attributed to the higher number of initial pores introduced by the addition of clay minerals, which fail to completely close due to lower vitrification of the materials with high Kln. Additionally, at 900–1200 °C, CS is more sensitive to the Ilt content than to the Kln content (Fig. 5a–c). A significant reduction in CS is observed with increasing Ilt content at 900 °C (from 20/0 to 50/0), while a notable increase occurs at 1050 and 1200 °C. In contrast, CS remains consistent with total clay mineral content when Kln dominates the clay mineral.

Furthermore, most formulations show increased CS at higher firing temperatures (Fig. 5e–g), in line with existing literature^47,48^. Two exceptions are: (1) the 50/0 formulation shows reduced CS at 1350 °C compared to 1200 °C, likely due to the formation of cracks from excessive liquid sintering (Fig. 2o); (2) for bricks with 20% total clay mineral content, lower CS is observed when increasing the temperature from 1050 to 1200 °C (Fig. 5e). This is attributed to the high quartz content in the starting clays. Quartz typically transforms into cristobalite above 1200 °C. Upon cooling, the formed cristobalite undergoes transition from β- to α-phase between 280 and 190 °C, resulting in ~5% volumetric shrinkage. This transformation may induce microcracks within the matrix, compromising structural integrity^49^. Importantly, because this inversion is reversible, repeated thermal cycling through the cristobalite inversion window may accumulate damage, severely degrade long-term durability, and ultimately lead to complete destruction of bricks after a sufficient number of cycles^50^. The considerable increase in cristobalite is confirmed by XRD in Fig. 5h, i. However, no further CS reduction is observed from 1200 to 1350 °C, likely due to enhanced sintering at 1350 °C, which imparts a more robust matrix capable of resisting cristobalite phase inversion.

To better visualize the CS value under different clay mineral assemblages, contour maps for 60 formulations are presented in Fig. 5j–m. Specifically, achieving a high CS (53.6 MPa) at 900 °C requires a low total clay mineral content and a high Kln/(Kln+Ilt) ratio. However, the clay mineral content should also be sufficient to ensure proper extrusion in practical brickmaking. At 1050 and 1200 °C, the trend reverses: a high clay mineral content and a low Kln/(Kln+Ilt) ratio improve CS, reaching peaks of 126.2 MPa and 142.4 MPa, respectively. For bricks fired at 1350 °C, achieving a high CS (172.4 MPa) requires avoiding extreme values for both clay mineral content and the Kln/(Kln+Ilt) ratio. These results demonstrate that by precisely adjusting the initial clay minerals, the brick CS can be significantly enhanced.

**Fig. 6:**
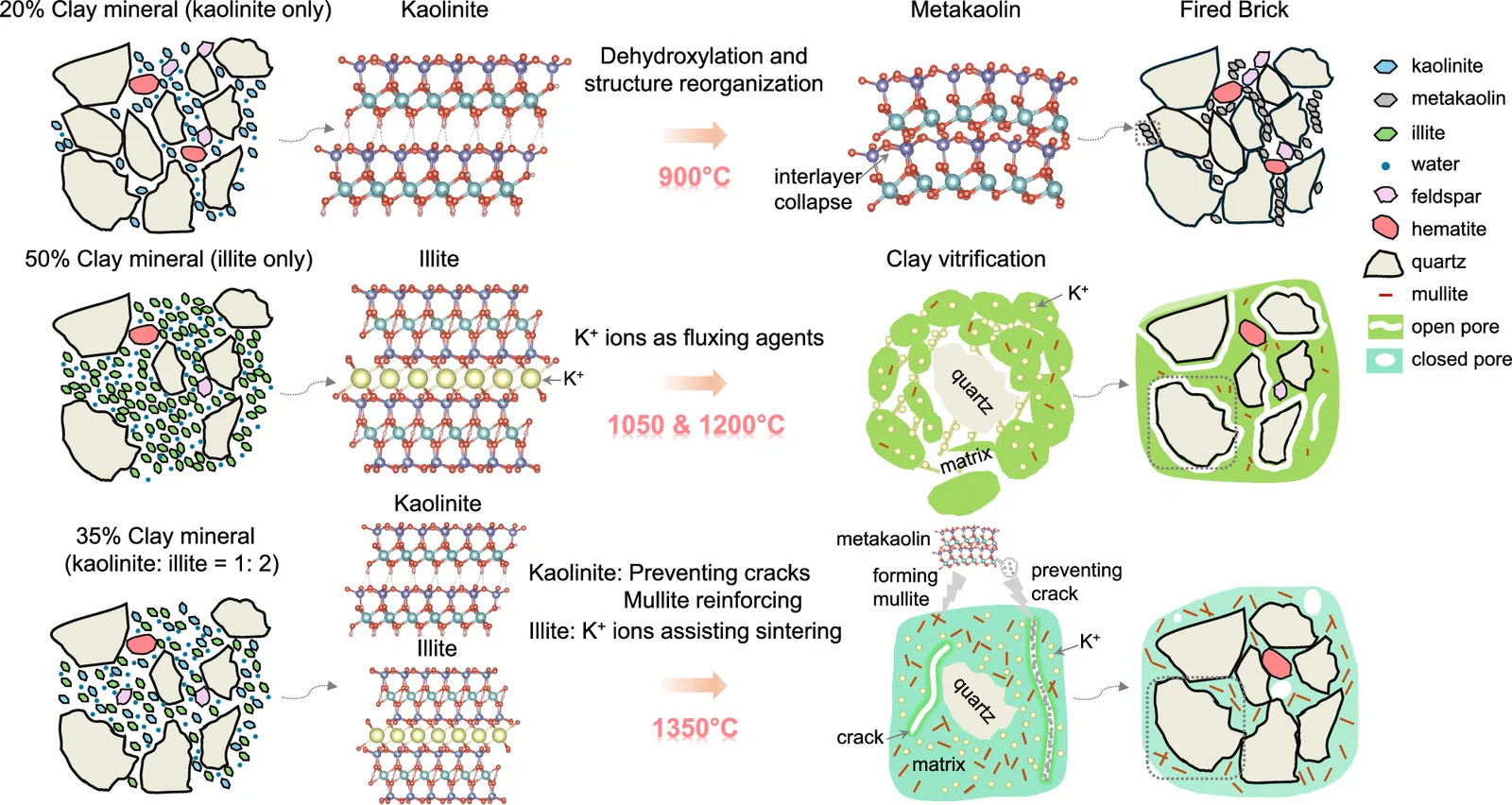
Proposed optimal clay formulations for enhanced compressive strength of bricks fired across 900–1350 °C, with underlying mechanistic insights.

Because the specimens prepared in this study were brick buttons rather than full-size units, a size effect must be considered: under identical pression, drying, and firing conditions, the CS of full-size bricks (230 × 110 × 75 mm) is conformed to be approximately 0.85 times that of the brick buttons (see comparisons in Supplementary Table 3 and Supplementary Note 5). This reduction is logically expected because larger specimens are more difficult to sinter uniformly, leading to heterogeneity in densification and greater residual porosity and internal defects. Applying this conversion factor, full-size bricks produced using the optimal formulations at different firing temperatures are estimated to reach approximately 2, 6, 7, and 8.5 times the minimum required CS (17.2 MPa) for Grade Moderate Weathering (MW) bricks, as specified by ASTM C62^51^. Meanwhile, these optimal formulations clearly outperform bricks derived from a wide range of industrial clays reported globally, including substantially higher CS and bulk density, together with lower WA (Supplementary Table 4). The recommended optimal clay formulations for enhanced CS at different firing temperatures are illustrated in Fig. 6.

### The importance of firing temperature

According to the above analyses, key properties of fired bricks are strongly influenced by starting clay minerals, with these effects varying significantly with firing temperature. This variability is primarily due to the differing responses of Kln and Ilt to heating, governed by their crystal structures (1: 1 or 2: 1) and chemical compositions (Al: Si ratio and fluxing agent concentration).

Since in most brick manufacturing scenarios, where Kln coexists with Ilt, and firing temperatures are around 1050 or 1200 °C^52^, the lower vitrification degree of Kln compared to Ilt results in lighter brick colors. This difference in vitrification also necessitates the coexistence of Kln and Ilt to achieve a high mullite content at 1050 °C, as discussed in Section “Physical and Mechanical Properties of Fired Bricks”. However, vitrification no longer limits mullite formation from metakaolin at 1200 °C.

**Fig. 7:**
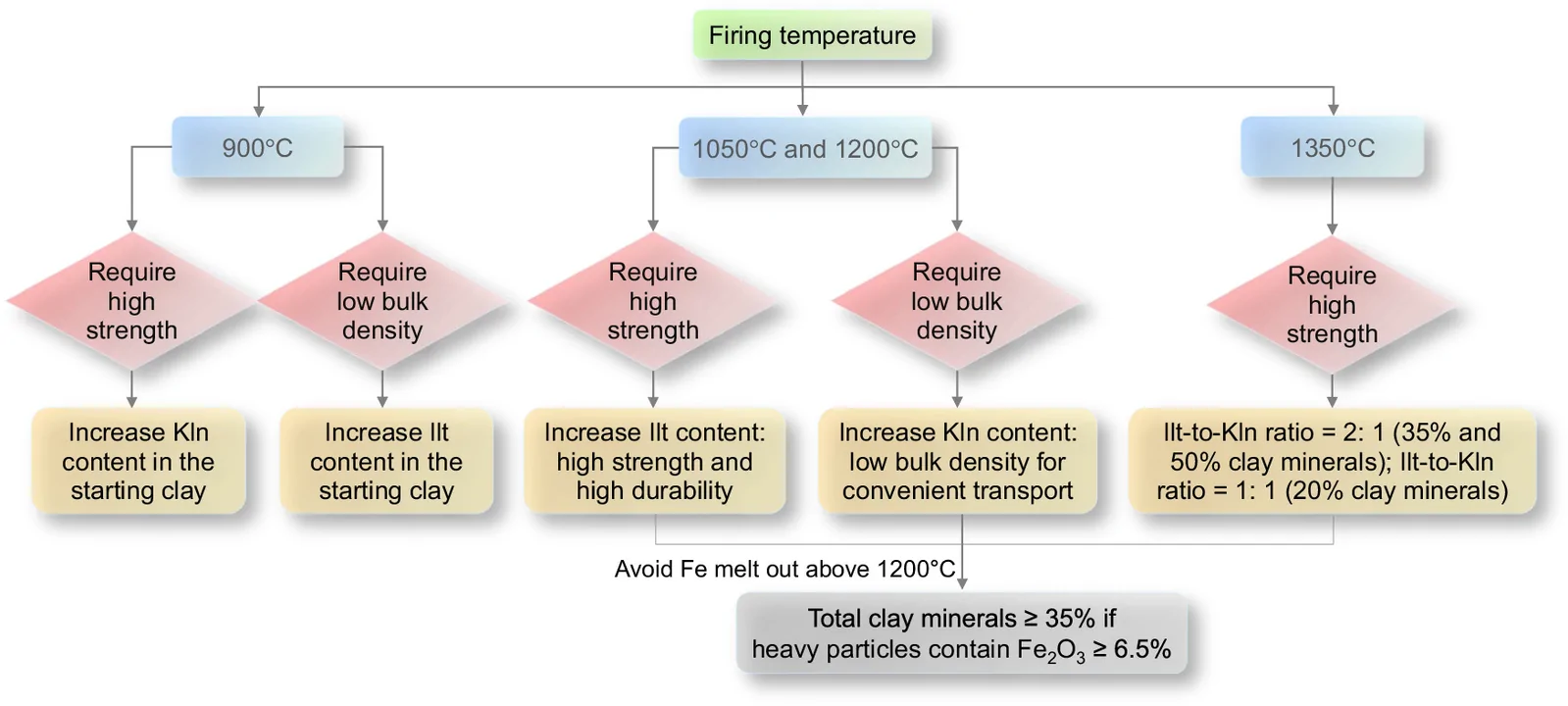
Temperature-dependent decision chart for the selection of Kln and Ilt for brick fired across 900–1350 °C. Kln kaolinite, Ilt illite.

For applications requiring lower bulk density (e.g., for easier transport), at 1050 and 1200 °C, increasing the Kln content can be beneficial, although this compromises the durability of bricks by increasing AP. Conversely, if higher bulk densities are required (enhancing durability and strength), a higher Ilt content in the starting clay is recommended. Additionally, to prevent vitrification of hercynite and hematite on the brick surface, particularly when Hp contains ≥6.5% of Fe_2_O_3_, the clay mineral should exceed 35%. Specific considerations also apply at the two less commonly used firing temperatures of 900 °C and 1350 °C. At 900 °C, Ilt fails to vitrify, weakening brick strength and making it unsuitable as a primary starting clay. At 1350 °C, optimal strength is achieved with an Ilt-to-Kln ratio of either 2: 1 or 1: 1, depending on the total clay mineral content. Based on these findings, a decision chart linking the firing window to the recommended Kln and Ilt in the starting clay is provided in Fig. 7.

### Correlations among diverse brick properties

Firing temperature impacts not only the thermal evolution of raw clays and related brick properties but also the interrelationships among these properties. While prior studies have frequently reported strong linear correlations (*R*^2^ ≥ 0.90)—such as a negative relationship between WA and firing shrinkage^53,54^, a positive correlation between CS and bulk density^13,55^, a negative correlation between CS and WA^13,55,56^, and a negative correlation between WA and bulk density^57^ - the present study did not observe similarly strong relationships after systematically evaluating 60 different formulations.

**Fig. 8:**
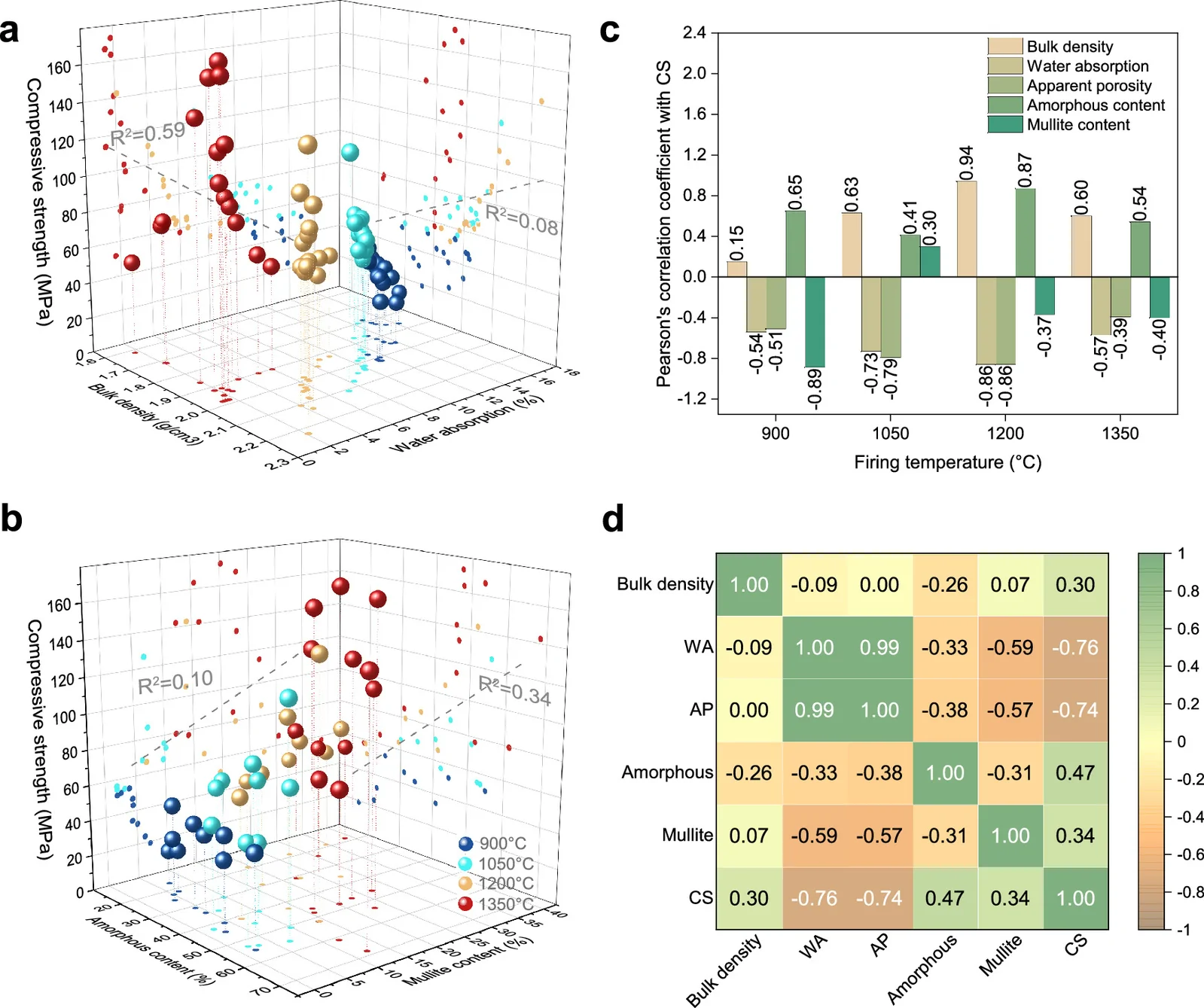
Relationships between physical properties, phase composition, and compressive strength of fired clay formulations. **a** Three-dimensional distributions and corresponding two-dimensional projections of bulk density, water absorption (WA), and compressive strength (CS) for 60 formulations. **b** Three-dimensional distributions and corresponding two-dimensional projections of amorphous content, mullite content and CS for 39 formulations. Dashed lines represent ordinary least-squares linear fits; the corresponding coefficients of determination (*R*²) are provided in each panel. **c** Pearson’s correlation coefficients (*r*) between CS and the other properties, calculated separately at each firing temperature and using the combined dataset across 900–1350 °C. The exact sample sizes used for the correlations are indicated in Section “Methods”. Correlations were classified as strong ( | *r* | > 0.75), moderately strong (0.50 < | *r* |   ≤  0.75) or weak ( | *r* | ≤ 0.50). Source data are provided as a Source Data file.

The highest coefficient of determination (*R*^2^) found in this study is only 0.59 for the CS-WA relationship, followed by 0.34 (CS-amorphous content), 0.10 (CS-mullite content), and 0.08 (CS-bulk density) (Fig. 8a, b). These values suggest that the strong linear correlations reported may only be applicable within narrow temperature or compositional ranges and may become obscured when data across the entire temperature range are combined. Indeed, the broader firing range examined here (900–1350 °C) reveals temperature-dependent correlations between CS and other properties. The strongest correlations occur at 1200 °C, where four of the five relationships have | *r* | >0.75. At 900 and 1050 °C, four and three relationships, respectively, are moderately strong to strong ( | *r* | >0.50), whereas those at 1350 °C are weak to moderately strong. In addition, a weak correlation between CS and bulk density at 900 °C (*r* = 0.15, Fig. 8c) is likely due to the unique behavior of Kln, which undergoes both significant shrinkage and water loss. At 1350 °C, the excessive vitrification of Ilt results in cracks and formation of closed pores, leading to irregular relationships among CS, bulk density, and WA.

According to quantitative analysis, the importance of temperature-combined factors influencing CS ranks as follows: WA (–0.76) > AP (–0.74) > amorphous content (0.47) > mullite content (0.34) > bulk density (0.30) (Fig. 8d). These correlations strongly depend on temperature (Fig. 8c), peaking mostly at 1200 °C, except for mullite content. Although the correlation between bulk density and CS is not statistically significant at the 95% confidence level (*p* = 0.06, Supplementary Fig. 26) when data from all temperatures are combined, temperature-separated analyses reveal a strong correlation at 1200 °C, moderately strong correlations at 1050 and 1350 °C, and a weak correlation at 900 °C (Fig. 8c). Therefore, the unexpectedly low impact of bulk density (*r* = 0.30) compared to literature is mainly due to its weak correlation with CS at 900 °C.

Interestingly, although mullite is widely acknowledged to strengthen ceramics by forming interlocking acicular crystal networks, refining Griffith flaws, and mismatched thermal expansion coefficient with glass phase matrix^58–61^, we have identified a negative correlation between mullite and CS at 900, 1200, and 1350 °C (the correlation coefficient at 1050 °C is also weakly positive at 0.3, Fig. 8c). This may be attributed to that mullite typically forms at the expense of the amorphous/glass phase. The latter contributes more significantly to CS development by facilitating sintering and particle bonding, as indicated by its stronger correlations with CS at 1050, 1200, and 1350 °C than those of mullite (Fig. 8c). Supporting this observation, nanoindentation statistics for 35/33/1350 and 35/100/1350 show lower hardness in mullite-bearing amorphous regions than in mullite-deficient amorphous regions (Supplementary Fig. 27). For 35/33/1350, the mean hardness (49 indents per sample) was 9.50 ± 0.90 GPa in mullite-deficient regions versus 8.64 ± 0.75 GPa in mullite-rich regions; for 35/100/1350, it was 9.58 ± 0.55 GPa versus 7.71 ± 0.96 GPa, respectively. Given that lower hardness is generally associated with lower local mechanical resistance, these results further suggest that the glassy/amorphous matrix plays a critical role in strengthening fired bricks. In addition, it can be reasonably inferred that the glass matrix is more important to fired bricks than other ceramic products, as the clayey materials used in brickmaking are generally coarser in particle size, creating larger initial voids and thus requiring more glass to achieve effective bonding. In fact, phase-related factors (amorphous and mullite contents) only play dominant roles at a low temperature of 900 °C, while above 1050 °C, porosity-related factors (WA, AP, and bulk density) are co-dominant (Fig. 8c). Inadvertent differences in the fine-particle content of Hp among different formulations may also affect particle packing, sintering, and pore development, thereby contributing to data scatter and weakening the observed correlations. Nevertheless, brick CS is governed by a combination of inter-related factors, including porosity (open and closed), phase composition and contact patterns, the degree of particle bonding (sintering), and the presence of cracks.

## Discussion

This study introduces a KIQF system for precisely adjusting the inherent phase composition of natural clays, thus enabling their individual contributions to brick performance to be evaluated across a broad and industrially relevant compositional range. Specific focus was put on kaolinite and illite—the two most common clay minerals in brick clay. We systematically varied total clay mineral content (20, 35, and 50%), kaolinite-to-total clay mineral ratios (0, 33, 50, 66, and 100%), and firing temperatures (900, 1050, 1200, and 1350 °C) and created 60 distinct brick formulations. Comprehensive material characterization revealed that optimal clay mineral assemblages are highly dependent on firing temperature, fundamentally governed by the unique crystalline structures of kaolinite and illite and their temperature-dependent thermal behaviors.

At 900 °C, formulations with 20% clay minerals composed entirely of kaolinite exhibited enhanced CS (53.6 MPa) and reduced WA (11.38%), attributed to the shrinkage from dehydroxylation and subsequent structural reorganization of kaolinite. In contrast, at 1050 and 1200 °C, bricks with 50% clay minerals predominantly consisting of illite demonstrated superior strength (126.2 and 142.4 MPa), likely due to the abundant K⁺ ions in illite promoting sintering and densification. The highest CS (172.4 MPa) and lowest WA (1.3%) were achieved at 1350 °C using a formulation with 35% clay minerals, where one-third of the clay minerals were kaolinite, effectively mitigating over-firing issues associated with illite vitrification.

Beyond these findings, an unexpected negative correlation between mullite content and CS is revealed, with Pearson correlation coefficients of −0.89, −0.37, and −0.40 at 900 °C, 1200 °C, and 1350 °C, respectively. This suggests that mullite formation may consume excessive glass phases that are more crucial to brick strength due to enhanced sintering and particle bonding. The formation of mullite and amorphous/glass phases is influenced by clay mineral content, Al: Si ratios, fluxing agents, and firing temperature. Additionally, the widely reported linear relationships among various technological properties of bricks persist only within narrow temperature ranges and deteriorate over the broader 900–1350 °C interval, due to the temperature-sensitive transformations of kaolinite and illite. Quantitative correlation analyses indicated that phase-related factors (amorphous and mullite contents) primarily influence CS at lower temperatures (900 °C), while porosity-related factors (WA and bulk density) become dominant above 1050 °C.

Other notable findings include that increased clay mineral content and higher kaolinite-to-illite ratios result in lighter brick colors because higher fluidity of the illite-based glass facilitates the growth of larger hematite crystals. However, excessive vitrification of illite from the interlayer K⁺ leads to hercynite exudation above 1200 °C and the formation of closed, rounded pores at 1350 °C, causing defects such as approximately 10% expansion and large cracks.

Through strategic tuning of clayey raw materials, our study demonstrates that the CS of bricks can achieve 2-, 6-, 7-, and 8.5-fold over the ASTM Moderate Weather strength requirement at 900, 1050, 1200, and 1350 °C firing. However, differences in clay mineral chemistry, crystallinity, particle size, and accessory minerals may alter phase transformations and firing behavior. Thus, kaolinite-illite-rich clays from other deposits may follow similar general mechanisms but exhibit slightly different optimum compositions. Nevertheless, insights from this work offer practical guidance for industrial brick manufacturing and provide a framework to ultimately explain the role of different components in determining key brick performance.

## Methods

### Materials

Three raw materials—Hp, Kln, and Ilt, were used. Hp derived from a commercial clay mix supplied by Austral Bricks Pty. Ltd. in Queensland, Australia. Kln and Ilt were supplied by Austral Bricks Pty. Ltd. and Guzhang Shan Lin Shi Yu Mineral Co., Ltd., respectively. Sodium hexametaphosphate (powder, purity of 96%) was purchased from Merck Pty. Ltd. and used as received.

### Fabrication process of fired bricks

The preparation of clay bricks could be primarily divided into three steps: molding, drying, and firing. First, the required amounts of Hp, Kln, and Ilt (totally 30 g) were homogenized in an electrical blender (KitchenAid Classic) for 30 min (Table 1). After adding 12% of water, the mixtures were stirred for 5 min and compacted in a stainless-steel die (Φ × 40 mm) at a constant load of 5 tons for 1 min (RK 80-ton press). The applied load was released immediately after the dwell period. Then, the collected green bodies were dried at 110 °C for 24 h and fired in static air (Nabertherm LHT 02/16) at 900, 1050, 1200, and 1350 °C (5 °C min^−1^; 2 h soak), followed by furnace cooling to 25 °C. A schematic illustration of the preparation procedure is provided in Supplementary Fig. 28. In addition, full-size bricks (230 × 110 × 75 mm) were prepared for CS comparison with brick buttons (Supplementary Note 5).

### Raw materials characteristics

The chemical compositions of Hp, Kln, and Ilt were analyzed using a Bruker S8 TIGER XRF spectrometer with a 4 kW Rh X-ray tube. Before the test, an aliquot of powder sample (0.74 g) was fused into a glass disc using a lithium metaborate/lithium tetraborate flux (50: 50 LiT: LiM, 0.5 wt% LiI) in a platinum crucible using an electric fusion furnace (Katanax, model X600). The particle size distributions of Hp, Kln, and Ilt were analyzed using a Mastersizer 3000 laser particle size analyzer with deionized water as the dispersion medium. The volume-weighted mean diameter was calculated using the following equation:

1 $${D}_{[4,3]}=\frac{\sum {n}_{i}{d}_{i}^{4}}{\sum {n}_{i}{d}_{i}^{3}}$$

where ${D}_{[4,3]}$, ${n}_{i}$, and ${d}_{i}$ are volume-weighted mean diameter, number diameter of particles in size class, and diameter of particles in size class, respectively. Thermogravimetry/derivative thermogravimetry analyses of Hp, Ilt, and Kln were performed on a Netzsch STA4493F from 25 to 1400 °C at a heating rate of 10 °C min^−1^ in a 50 mL min^−1^ airflow.

### Expansion/shrinkage of clay mix

The expansion/shrinkage of clay mixes during firing were investigated using two methods. Cylindrical specimens (Φ 6 × 25 mm) were prepared from green bodies, and their in-situ dimensional changes were measured using a Netzsch 402 C thermal dilatometer from 25 to 1350 °C at a ramping rate of 5 °C min^−1^ under static air. For the dilatometry tests, representative formulations were selected, including 35/0, 35/33, 35/50, 35/66, 35/100, 20/50, and 50/50. Additionally, firing shrinkage of brick buttons was calculated using the equation provided below:

2 $${{{\rm{Firing\; shrinkage}}}}=\frac{D-{D}_{0}}{{D}_{0}}\times 100\%$$

where ${D}_{0}$ and D represent the diameter of brick buttons before and after firing, respectively. Two specimens were tested for each formulation, and the reported values were the averages of the duplicates.

### In-situ phase evolution and Rietveld refinement

For in-situ phase evolution, high-temperature XRD measurement was conducted on a Rigaku SmartLab X-ray diffractometer (Cu Kα, 40 kV, 40 mA) equipped with an Anton Paar HTK-1200 N chamber. XRD patterns were collected from 7° to 45° 2θ at a step size of 0.02°. The temperature increased from 25 to 1200 °C with a ramp rate of 5 °C min^−1^ under 100 mL min^−1^ dry airflow. Data were collected with an interval of 25 °C. The sample height was aligned at each temperature to prevent expanded sample holder height compromising XRD reflections. As for the quantitative phase analysis, XRD patterns were acquired using a Bruker D8 Advance diffractometer (Co Kα, 35 kV, 40 mA) from 2° to 90° 2θ at a step size of 0.015° and a scan speed of 1.5° 2θ per min. A 10 wt.% internal standard (corundum) was used for quantification of amorphous content with details shown in Supplementary Note 6. Phase identification of the XRD pattern was conducted in DIFFRAC.EVA version 7.0 in ICDD PDF-5 + 2025 database. Quantitative phase analysis was conducted through Rietveld refinement in DIFFRAC.TOPAS version 7.0 with the Rwp (weighted profile residual factor) value from each refinement below 10%. In addition, to identify the swelling clay minerals in raw materials, sedimentation experiments were applied to prepare clay slides for XRD measurement with details shown in Supplementary Note 7.

### Microstructural characterizations

The micromorphology of fired bricks was imaged using a field emission SEM (JEOL 7001 F) using backscattered electron imaging at 20 kV and 12 mA. An X-Max 80 (Oxford, UK) energy-dispersive X-ray spectroscopy system was applied to determine elemental compositions. To examine temperature effects, we analyzed the 35/50 formulation fired at 900, 1050, 1200, and 1350 °C. To examine the effect of total clay mineral content, we analyzed 20/0, 35/0, and 50/0 fired at 1350 °C, and to examine the effect of kaolinite-to-clay mineral ratio, we analyzed 35/0, 35/50, and 35/100 fired at 1350 °C. Sample preparation procedures are shown in Supplementary Note 8. The open and closed pore structures of 50/0/900 and 50/0/1350 were characterized by micro-CT (μCT 50, Scanco Medical, Switzerland) at 70 kVp and 114 μA with a 10.0 μm isotropic voxel size. Nanoindentation testing procedures on 50/33/1350 and 50/100/1350 were provided in Supplementary Note 9.

### Physical and mechanical properties of fired bricks

The 24-h cold WA of fired bricks were measured as per the ASTMC67/C67M standard^62^. The AP of fired bricks are measured using an Anton Paar Ultrapyc helium pycnometer with details provided in Supplementary Note 10. The bulk density of fired bricks was calculated using the following equation:

3 $${{{\rm{B}}}}{{{\rm{ulk\; density}}}}=\frac{m}{{{{\rm{\pi }}}}{r}^{2}l}$$

where m, r, and l stand for the mass, radius, and thickness of fired bricks, respectively. Measurements were taken at five positions for each dimension, and the average value was used. For the WA, AP, and bulk density measurements, 60 formulations were prepared, and two specimens were tested for each formulation; the reported values were the averages of the duplicates. CS tests were conducted on fired brick buttons (Φ 40 × 12 mm) using the Instron 6800 universal testing machine, with the maximum bearing capacity of 300 kN and a loading rate 1 mm per min. In total, 144 compression tests were performed on 72 specimens (Fig. 2m). Tests were in duplicate, and the final CS values were reported as the mean results.

### Statistical analysis of relationships among brick properties

Two complementary statistical analyses (two-tailed Pearson’s correlation) were conducted to examine relationships among brick properties. For temperature-specific correlations (Fig. 8c), correlations of WA, AP, and bulk density with CS were calculated using all available formulations (*n* = 15, Table 1). For amorphous and mullite contents, correlations were calculated using the three Kln/(Kln+Ilt) compositions for which quantitative phase data were available (0, 50, and 100%, *n* = 9 at each firing temperature). These compositions represent the two endmembers and the midpoint of the investigated compositional range. In addition, pooled correlations were assessed across all temperatures (Fig. 8d) using *n* = 39 data points: the 0, 50%, and 100% at four temperatures (36 samples), plus three additional samples (20/33, 35/33, and 50/33) at 1350 °C, for which phase analysis was also available. All tests used a 95% confidence level, and the corresponding *p*-values were reported in Supplementary Table 5 and Supplementary Fig. 26.

## Supplementary information

Supplementary Information

Transparent Peer Review file

## Source data

Source Data ZIP

## Data Availability

The authors declare that the data supporting the findings of this study are available within the paper and its supplementary information files. Source data are provided with this paper.
