# Supplementary material for: From ancient practice to modern innovation solving the clay mineral puzzle in brickmaking
Source: Nat Commun. 2026 Oct 14;17:11596. doi: 10.1038/s41467-026-78652-0 (PMC13752296; doi:10.1038/s41467-026-78652-0)
Supplement: Supplementary file 1 [file 41467_2026_78652_MOESM1_ESM.pdf]

Supplementary Information

From Ancient Practice to Modern Innovation Solving the Clay Mineral Puzzle in Brickmaking

Sen Wang[1,2], Lloyd Gainey[3], Lihui Liu[4], Zijun Zeng[1,2], Shusheng Xiao[2], Runliang Zhu[1], Sara Couperthwaite[2], Yuantong Gu[2] & Yunfei Xi[1,2]*

[1]State Key Laboratory of Advanced Environmental Technology, Guangzhou Institute of Geochemistry, Chinese Academy of Sciences (CAS), Guangzhou 510640, China

[2]School of Mechanical, Medical and Process Engineering, Faculty of Engineering, Queensland University of Technology (QUT), Brisbane, Queensland 4001, Australia

[3]Austral Bricks, Rochedale, Queensland 4123, Australia

[4]School of Environmental and Municipal Engineering, Lanzhou Jiaotong University, Gansu 730070, China

Corresponding Author: xiyf@gig.ac.cn; y.xi@qut.edu.au (Y. Xi)

Supplementary Note 1. Preparation of Heavy Particle

Heavy particles (Hp) were derived from a commercial clay blend supplied by Austral Bricks Pty. Ltd., Queensland, Australia, originally used for brick manufacturing. Clay minerals were separated from the blend using a sedimentation technique referring Lopes, Morais [1]. Specifically, 500 g of the clay blend and 2 g of sodium hexametaphosphate were weighed and dispersed in a 4 L glass beaker filled with deionized water. Sodium hexametaphosphate works as a dispersant by enhancing electrostatic repulsion between clay mineral particles and preventing their agglomeration.[2] The obtained mixture was stirred and subjected to ultrasonic treatment for 30 minutes, and then left undisturbed in the beaker for 1 hours. Due to their fine particle size (typically < 2 μm) and negative surface charge, clay minerals remain suspended in water, as they tend to repel each other and stay evenly dispersed. In contrast, particles in the mix - such as quartz, hematite, mullite, and feldspar - exhibit larger particle sizes and higher settling velocities, causing them to settle at the bottom of the beaker. After sedimentation for 1 h, the supernatant containing suspended clay minerals was carefully decanted, leaving behind a slurry enriched in heavy particles. An additional 4 L of deionized water and 2 g of sodium hexametaphosphate powder were then added, and above process was repeated approximately 10 times until most of the clay minerals were removed. The final collected particles were vacuum dried at 105°C for 12 hours and referred to as Hp.

Supplementary Note 2. Purification of Kaolinite and Illite

Kaolinite (Kln) and illite (Ilt), obtained from separate source materials distinct from the brickmaking raw material, were independently purified using a sedimentation method similar to that employed for the preparation of heavy particles. However, unlike the heavy particle preparation - where the settled fraction was retained - the upper suspension enriched in clay minerals was collected, while the denser particles that settled at the bottom were discarded. The collected clay-rich suspension was transferred into 250 mL polyethylene bottles and centrifuged at 4025 × *g* for 10 minutes to remove excess water. The resulting clay slurry at the bottom of the bottles was carefully collected and dried in a vacuum oven at 105°C for 12 hours. The dried material was then ground using a ring mill for 30 seconds to obtain the desired powdered samples, referred to as Kln and Ilt.

Supplementary Note 3. Quartz Evolution among Different Bricks

Junior, Hotza [3] emphasized that quartz particles affect the mechanical properties of porcelain through both microstructural damage and reinforcement as skeleton. Unlike mullite and amorphous phase, quartz content remains relatively stable across varying Kln/(Kln+Ilt) ratios (Fig. 3g). The slight decrease in quartz content at 900°C as the Kln/(Kln+Ilt) ratio increases is due to the higher concentration of quartz impurity in Ilt than that in Kln (Supplementary Fig. 3). Quartz reacts with clay mineral-anhydrite or transforms into cristobalite at higher temperatures,[4] so its content decreases in the final bricks. The K^+ present in Ilt acts as catalysts to accelerate the transformation of quartz (Fig. 3g).[5] Additionally, a lower initial Hp content reduces quartz in the resulting bricks (Fig. 3h,i).

Supplementary Note 4. Effects of Total Clay Mineral Content on Water Absorption and Apparent Porosity

The effects of total clay mineral content on water absorption (WA) and apparent porosity (AP) depend on the relative proportions of Kln and Ilt. When Kln is the primary clay mineral, WA and AP increase as the total clay mineral content rises across 900-1350°C (Fig. 4a-d and 4e-h). This trend is attributed to the high initial porosity in the green body introduced by fine Kln particles. Due to limited vitrification capacity of Kln, these pores are not effectively reduced during firing, leading to higher WA. However, when Ilt is the dominant clay mineral, WA and AP increase with total clay mineral content at 900°C but decrease at 1050°C and 1200°C. The increase at 900°C mainly reflects the additional initial porosity introduced by added Ilt, whereas the decrease at 1050-1200°C is associated with Ilt softening and intense vitrification, which enhances particle bonding and reduces connected open pores. At 1350°C, Ilt vitrifies extensively, resulting in very low WA and AP (1.9% and 3.3%, respectively, for 50/0/1350), indicating near-complete densification. Besides, in mixtures where Kln and Ilt present in equal proportions (50: 50), WA and AP increase with total clay mineral content at 900°C and 1050°C, but decreases at 1200°C and 1350°C. This can be explained by the extensive vitrification of Ilt above 1200°C, which compensates for the increased initial porosity introduced by the additional clay minerals.

Supplementary Note 5. Preparation and Test of Full-size Bricks for Comparison of Compressive Strength with Brick Buttons

Size effects on measured compressive strength have been reported for clay bricks,[6] and this is relevant here because the strength requirements in ASTM C62 are specified for full-size units rather than laboratory-scale specimens. To quantify the size effect and enable comparison with ASTM C62, we prepared both full-size bricks (230 × 110 × 75 mm) and brick buttons (Φ 40 × 12 mm) from the same commercial brick clay supplied by Austral Bricks Pty. Ltd. Both specimen types were formed by uniaxial pressing under identical forming conditions in terms of applied pressure and dwell time - 0.622 MPa (equivalent to a load of 1 ton for the brick buttons and 20 tons for the full-size bricks, reflecting the different pressing areas) and 1 min, respectively - so that any strength difference could be attributed primarily to specimen size rather than processing variations. After demolding, all specimens were dried and fired under the same schedule: 110°C for 24 h, followed by firing at 900, 1050, 1200, and 1350°C with a 2 h soak at the peak temperature. Compressive strength was then determined following ASTM C67. As summarized in Table S3, the compressive strength of full-size bricks is consistently lower than that of the corresponding brick buttons, averaging ~0.85 of the button strength under the same forming and firing conditions.

Supplementary Note 6. Sample Preparation for Quantitative X-ray diffraction (XRD)

Approximately 1.80 grams of crushed bricks and 0.20 grams of corundum (used as an internal standard) were accurately weighed, mixed, and micronized for 6 minutes in absolute ethanol using a McCrone mill equipped with zirconia beads. The treated material was air-dried at 40°C overnight and subsequently utilized for back-pressing into XRD sample holders to minimize the preferred orientation.

In the raw TOPAS outputs, all identified crystalline phases, including corundum, were normalized to 100% without accounting for the amorphous phase. Therefore, the refined corundum content could exceed its known value of 10 wt%. To obtain the final phase compositions, all crystalline-phase contents were corrected against the known 10 wt% corundum content. After correction, the amorphous/glassy content was calculated as the difference between 100% and the sum of all corrected crystalline phases, including corundum. Corundum was then excluded, and the remaining crystalline and amorphous contents were converted to the original brick-sample basis and normalized to 100%. The corrected values were used for the quantitative phase compositions reported in the manuscript, whereas Supplementary Figs. 16-25 present the raw TOPAS refinement outputs.

Supplementary Note 7. Sample Preparation for Clay Fraction Identification

This clay-fraction preparation was an analytical procedure independent of the purification described in Supplementary Note 2. It was performed solely to identify the clay mineral species in the Hp, Kln, and Ilt samples and to determine whether any expandable clay minerals were present by XRD. A fraction of the crushed powder was mixed with water and concentrated ammonia in 25 mL glass beaker. The obtained mixture was hand shaken for 30 seconds, followed by sonication for 5 minutes and settling for another 5 minutes. This process aimed to isolate the fine fraction (clay minerals), typically less than 5 μm when suspended. Approximately 1 mL of the uppermost suspension, containing the finest particles and most enriched in clay minerals, was transferred using a pipette to a low-diffraction Si plate, where it was allowed to settle and dry overnight. These dried samples were labeled as "air" in this study. This method concentrates the clay mineral-dominant fraction, aiding in the identification of present clay minerals, particularly expandable ones. However, not all clay mineral particles entered the uppermost suspension because they were fractionated by particle size, with relatively coarser clay particles remaining lower in the suspension or settling to the bottom. Therefore, the ratios of clay minerals and other phases in this extract may differ from those in the bulk sample, and the results from the fine fraction are only qualitative. Furthermore, the air-dried slides were treated in an ethylene glycol atmosphere at 60°C for 12 hours and re-examined for XRD immediately. These treated samples were labeled as "glycol" in the study. Finally, the glycol treated samples were heated at 550°C for 1 hour for another batch of XRD test which labelled as "550°C" as can be seen in Supplementary Fig. 2.

Supplementary Note 8. Sample Preparation for Scanning Electron Microscopy Imaging

Fired brick pieces with dimensions of Φ20 × 5 mm (in diameter and height) were cut from initial specimens. After placing in a 30 mm diameter plastic mold, samples were embedded with beam stable epoxy and cured overnight under vacuum. Extensive milling and polishing procedures were applied to receive plane-parallelism of sample surfaces. The polish approach was as follows: Step 1 - Grind the sample face flat with a 220 grit diamond disc for 1 minute; Step 2 - Change the diamond disc to a 1200 grit and grind for 2 minutes; Step 3 - Change the disc to a 9 micron polishing pad and polish for 6 minutes; Step 4 - Change the disc to a 3 micron polishing pad and polish for 5 minutes; Step 5 - Change the disc to a 1 micron polishing pad and polish for 2 minutes. Between each step, water and ethanol were also used to clean samples thoroughly.

Supplementary Note 9. Nanoindentation Testing Procedure

The nanoindentation tests were performed using a Hysitron TI 950 TriboIndentor equipped with a Berkovich diamond pyramid tip. The instrument has a load resolution of 1 nN and a z-direction displacement resolution of 0.04 nm. Prior to testing, finely polished brick button specimens (Ø 40 × 12 mm) - 35/33/1350 and 35/100/1350, were firmly mounted on the nanoindenter stage, and the top surface was aligned parallel to the indenter to ensure accurate measurements.

All indentations were performed in displacement-controlled mode using the same 35 s loading-holding-unloading profile. During the loading segment, the indentation displacement was increased from 0 to the maximum displacement of 200 nm over 10 s. The maximum displacement was then maintained for 15 s to allow the time-dependent deformation to stabilize and reduce the influence of creep on the initial unloading response. During the final 10 s, the indenter was unloaded from the peak condition to zero load. The load-displacement curve was recorded continuously throughout the entire cycle, and the contact stiffness was determined from the initial slope of the unloading curve. Displacement control was selected instead of peak-load control to maintain a consistent indentation depth across microstructural constituents with different mechanical properties.

For the 35/33/1350 and 35/100/1350 samples, two regions were examined: 1) the amorphous matrix without obvious mullite crystals and 2) the amorphous matrix containing embedded mullite crystals. In each region, a 7 × 7 indentation array was conducted, yielding 49 measurements for calculating the hardness H according to the following equation:

$$H = \frac{F_{max}}{A_c} \tag{1}$$

where F_{max} is the maximum load in mN; A_c is the projected contact area, in nm^2. The mean hardness for each region was calculated by averaging the 49 measurements. The corresponding error bars in Supplementary Fig. 27 represent the sample standard deviation (SD) of 49 hardness measurements ($n = 49$), calculated by dividing the sum of squared deviations from the mean by $n - 1 = 48$ and taking the square root.

Supplementary Note 10. Apparent Porosity Test of Fired Bricks Using a Helium Pycnometer

Apparent porosity of the fired bricks was quantified by helium gas-displacement pycnometry, which measures the skeletal volume (i.e., the solid volume excluding helium-accessible void space). Owing to inert, monatomic nature of helium and small kinetic diameter, it readily penetrates fine, interconnected pores (down to ~2 nm in this study) that are difficult to access using many liquid-immersion methods, enabling reliable determination of skeletal volume/density. Prior to testing, specimens were oven-dried at 110°C overnight and cooled to room temperature in a desiccator. The geometric volume $V_{gxrdeom}$ was determined from caliper measurements of specimen diameter and thickness; measurements were taken at five positions for each dimension and averaged. The helium-accessible skeletal volume V_{He} was measured using a calibrated Anton Paar Ultrapyc 5000 helium pycnometer by pressurizing the sample chamber to a fixed target pressure of 18 psig and recording the equilibrium pressure after expansion into a reference volume, from which V_{He} was computed. Apparent porosity was calculated from the difference between geometric and skeletal volumes as:

$$Apparent\ porosity = \frac{V_{geom} - V_{He}}{V_{geom}} \times 100\% \tag{2}$$

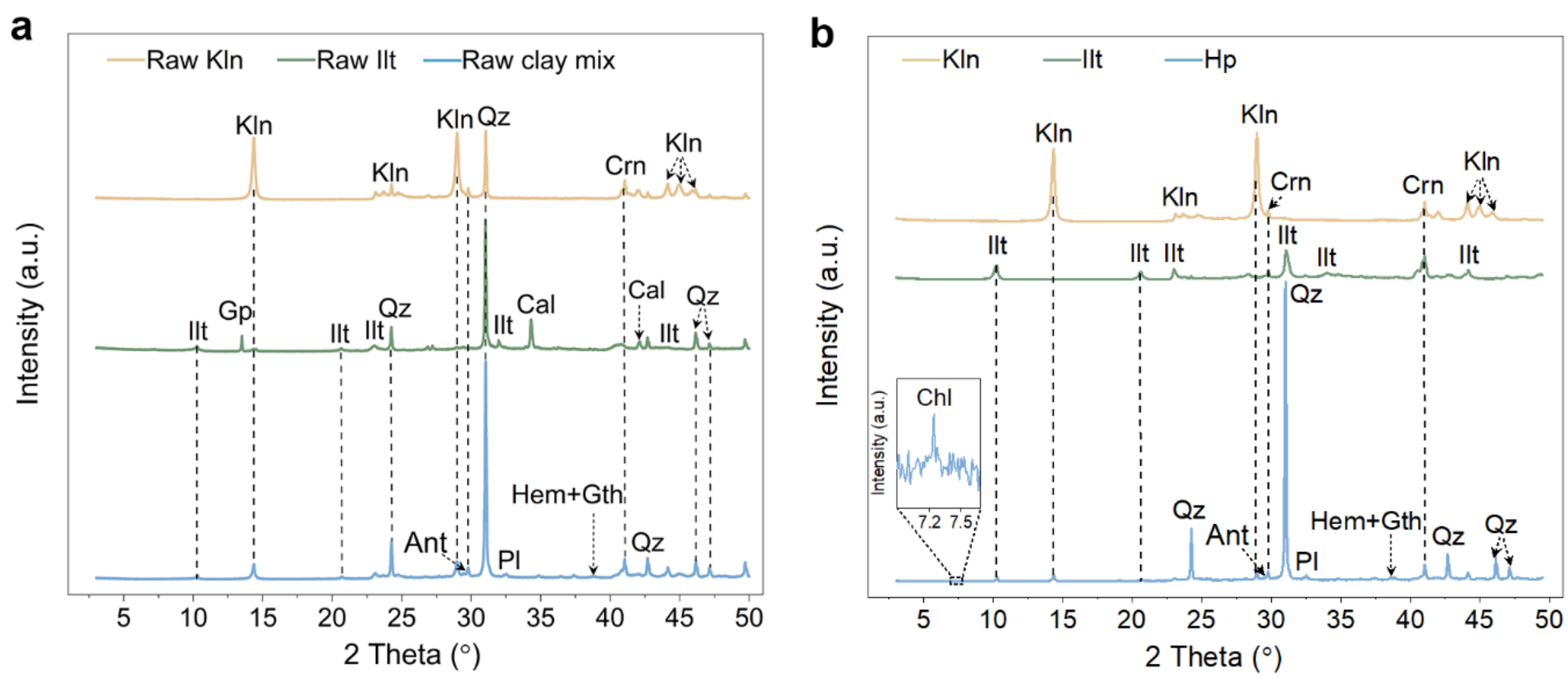

Supplementary Fig. 1 | X-ray diffraction (XRD) patterns of raw and purified starting materials. a XRD patterns of raw Kln, raw Ilt and the raw clay mixture. **b** XRD patterns of Kln, Ilt and the clay mixture (Hp) after purification. The inset in **b** shows magnified XRD patterns highlighting the characteristic reflections of chlorite. Qz, quartz; Hem, hematite; Ant, anatase; Crn, corundum (internal standard); Gp, gypsum; Cal, calcite; Gth, goethite; Pl, plagioclase; Chl, chlorite; Ilt, illite; Kln, kaolinite; a.u., arbitrary unit. Source data are provided as a Source Data file.

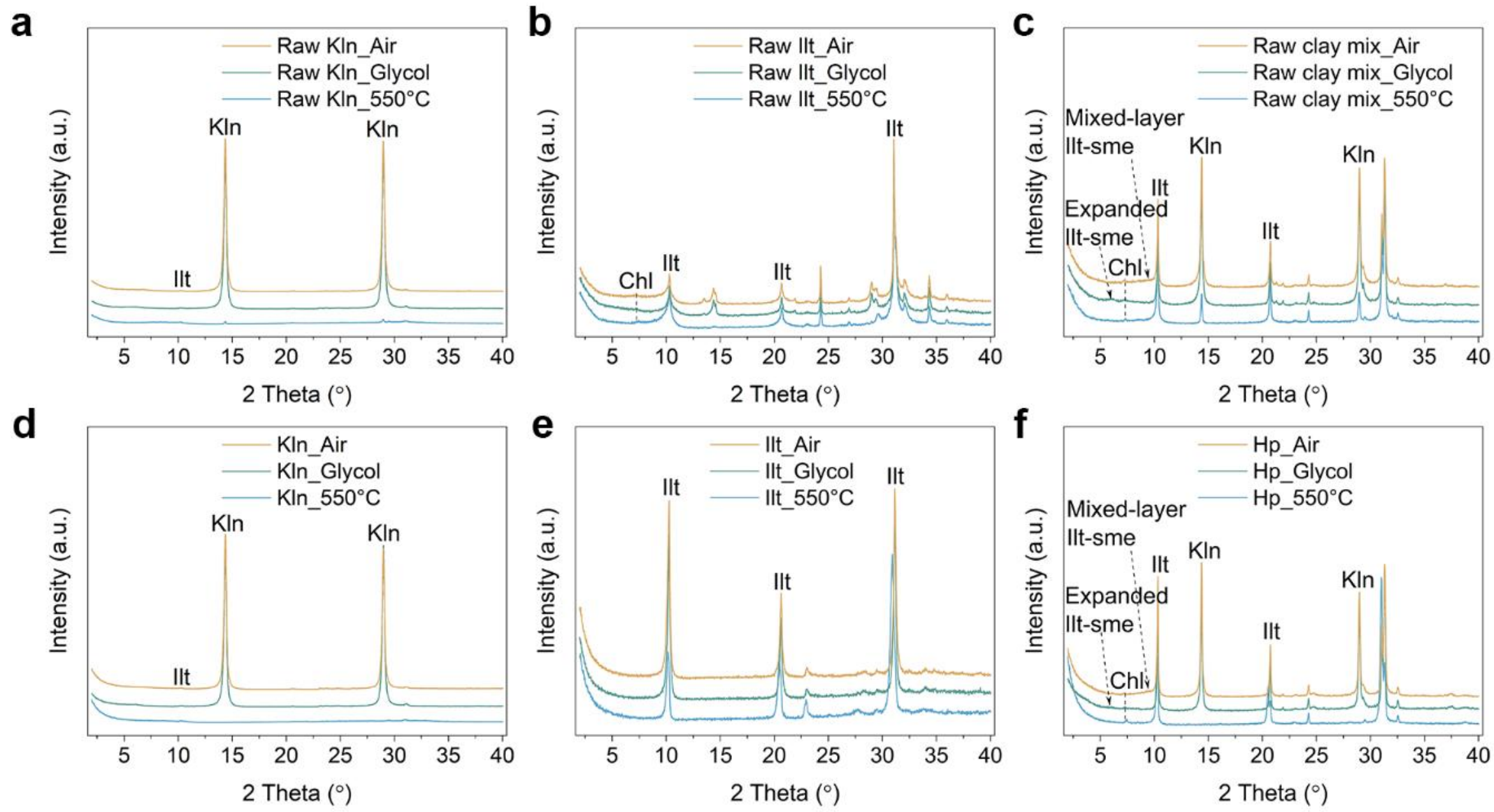

Supplementary Fig. 2 | X-ray diffraction (XRD) patterns of clay slides prepared from the fine fractions of raw and purified materials. **a** Raw Kln. **b** Raw Ilt. **c** Raw clay mixture. **d** Purified Kln. **e** Purified Ilt. **f** Purified clay mixture (Hp). Ilt, illite; Kln, kaolinite; Ilt-Sme, mixed-layer illite-smectite; Chl, chlorite; Hp, heavy particles. a.u., arbitrary unit. Source data are provided as a Source Data file.

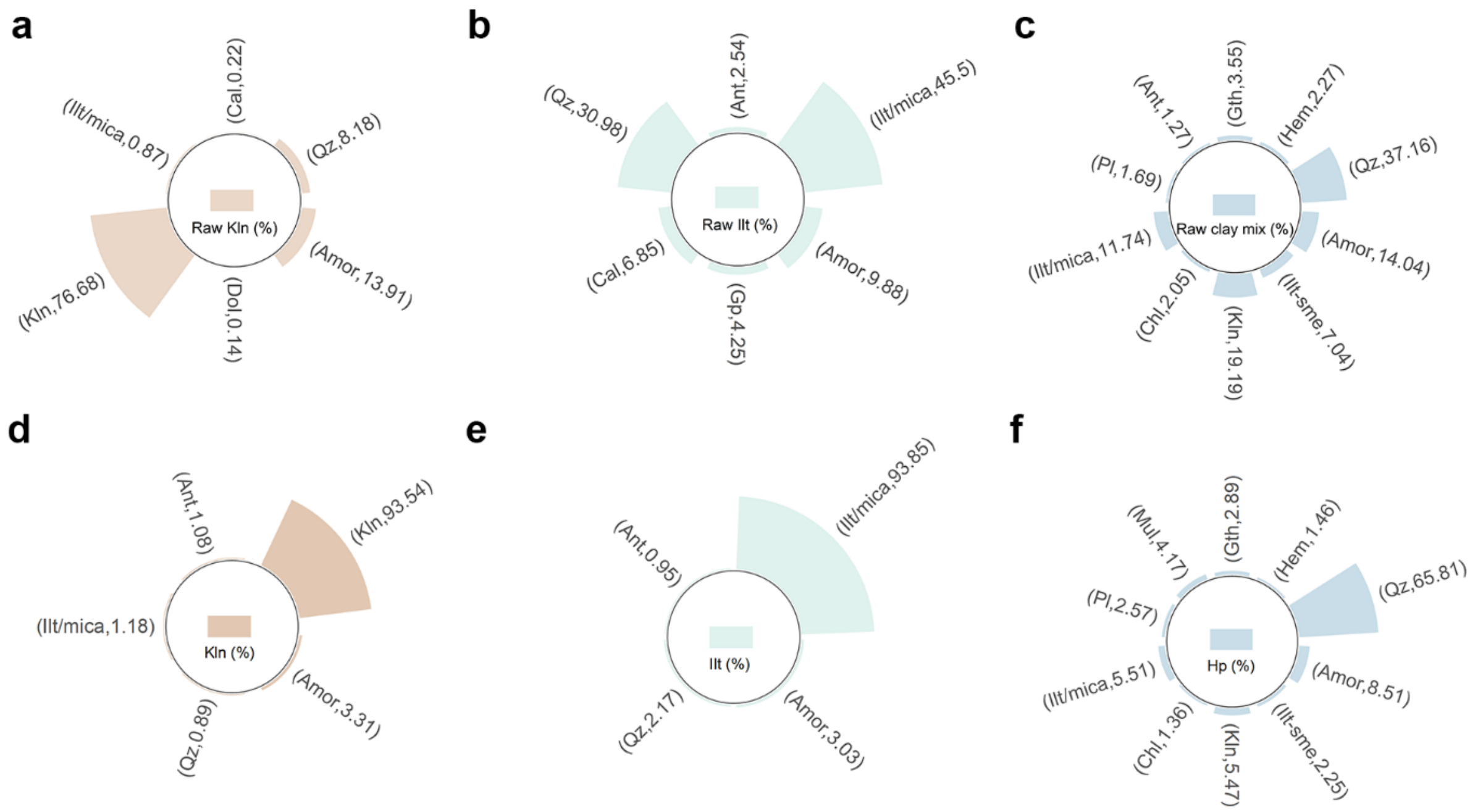

Supplementary Fig. 3 | Quantitative phase compositions of raw and purified starting materials. **a** Raw Kln. **b** Raw Ilt. **c** Raw clay mixture. **d** Purified Kln. **e** Purified Ilt. **f** Purified clay mixture. Qz, quartz; Hem, hematite; Ant, anatase; Mul, mullite; Gth, goethite; Gp, gypsum; Cal, calcite; Pl, plagioclase; Chl, chlorite; Ilt, illite; Kln, kaolinite; Sme, smectite; Amor, amorphous phase; Hp, heavy particles. Source data are provided as a Source Data file.

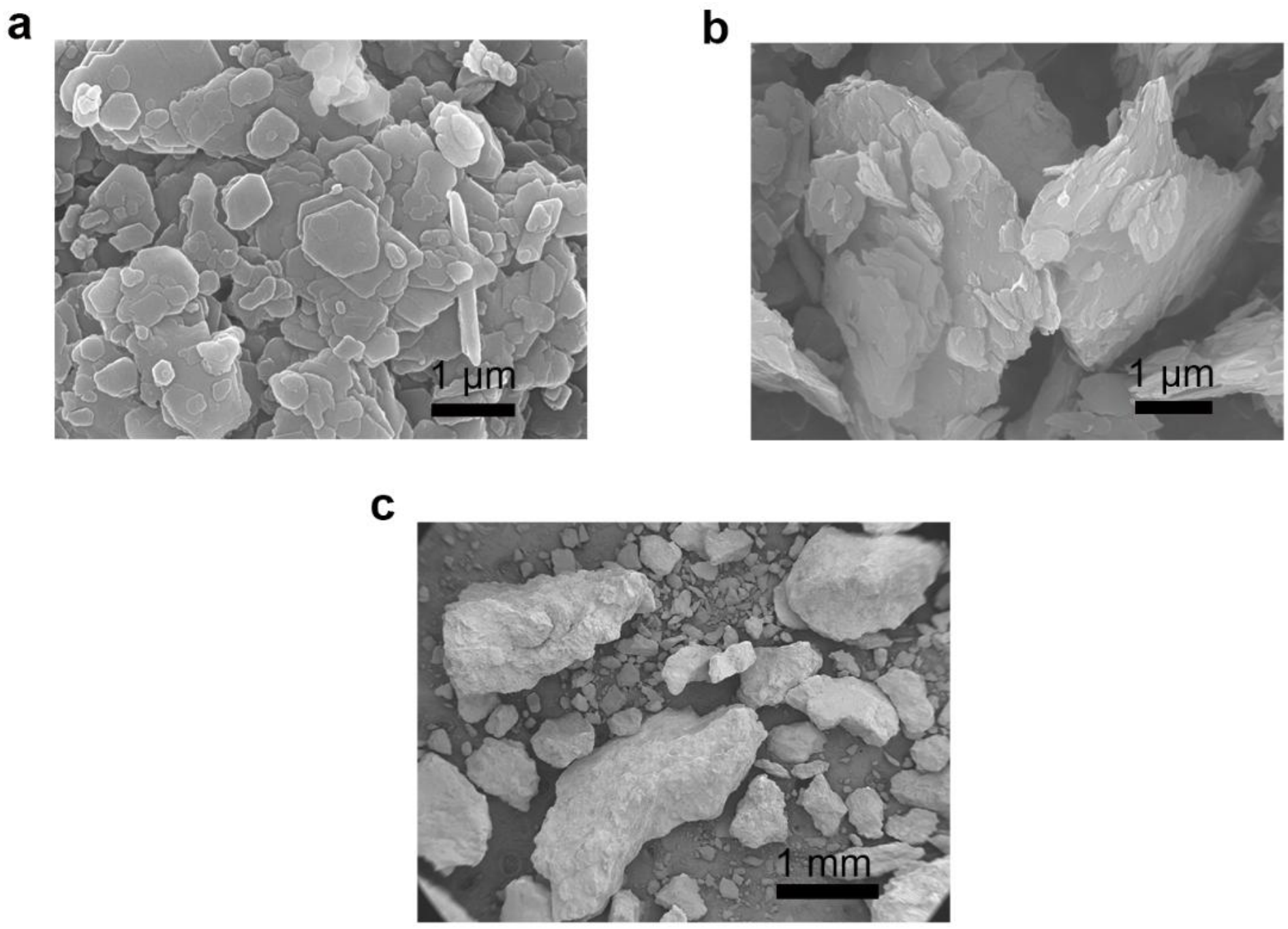

Supplementary Fig. 4 | Morphologies of the purified starting materials. Scanning electron microscopy images of **a** Kln, **b** Ilt and **c** Hp. Kln, kaolinite; Ilt, illite; Hp, heavy particles.

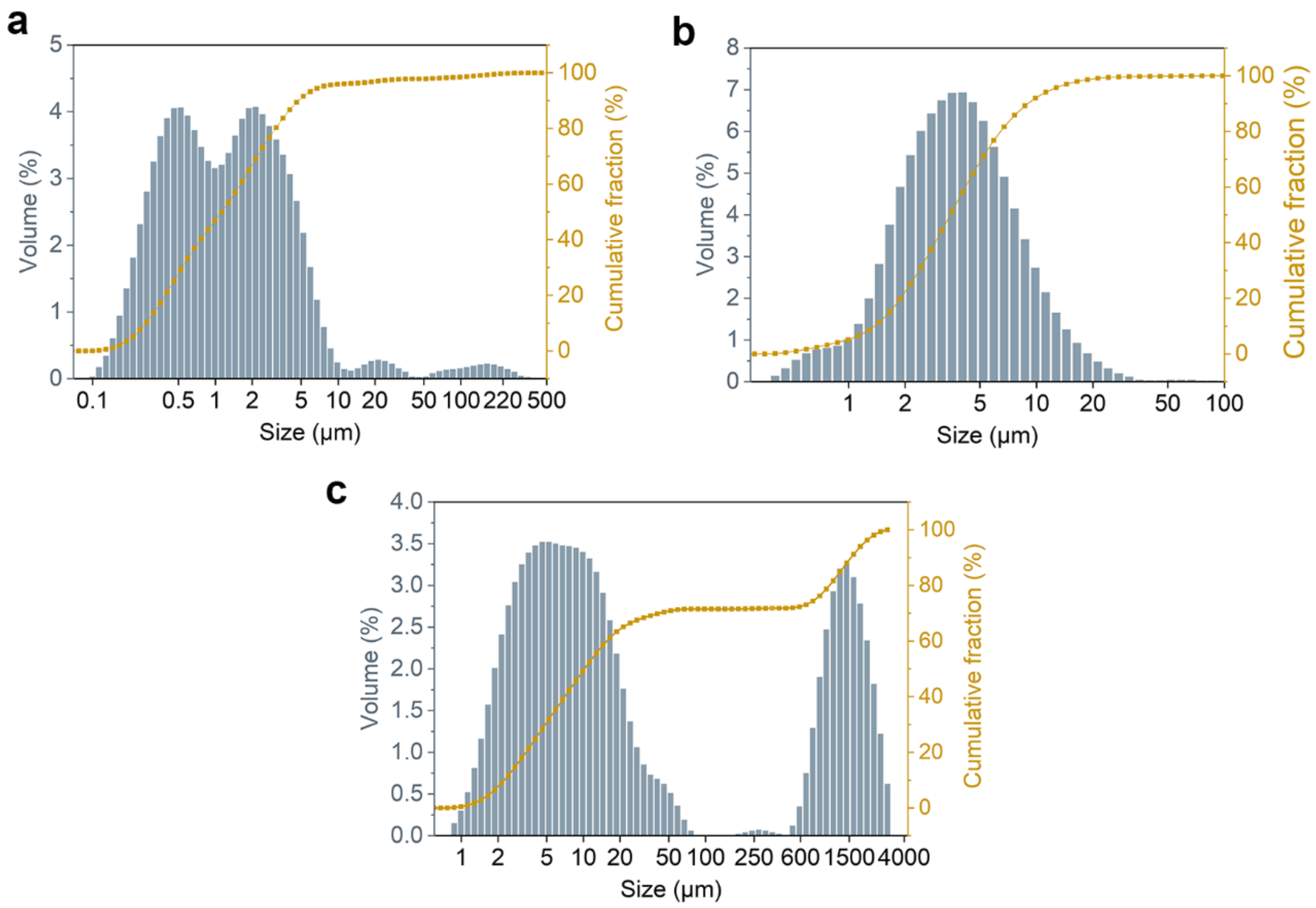

Supplementary Fig. 5 | Particle size distributions of the purified starting materials. Laser particle size distributions of **a** Kln, **b** Ilt and **c** Hp. Kln, kaolinite; Ilt, illite; Hp, heavy particles. Source data are provided as a Source Data file.

Supplementary Table 1. Particle-size distribution parameters of Kln, Ilt, and Hp.

Raw material	D_{10} (μm)	D_{50} (μm)	D_{90} (μm)	$D_{[4,3]}$ (μm)
Kln	0.309	1.28	5.39	5.75
Ilt	1.56	3.94	10.2	5.28
Hp	2.54	11.5	1760	460

Note: D_{10}, D_{50}, and D_{90} are the particle diameters below which 10%, 50%, and 90% of the total particle volume is contained, respectively; $D_{[4,3]}$ is the volume-weighted mean diameter.

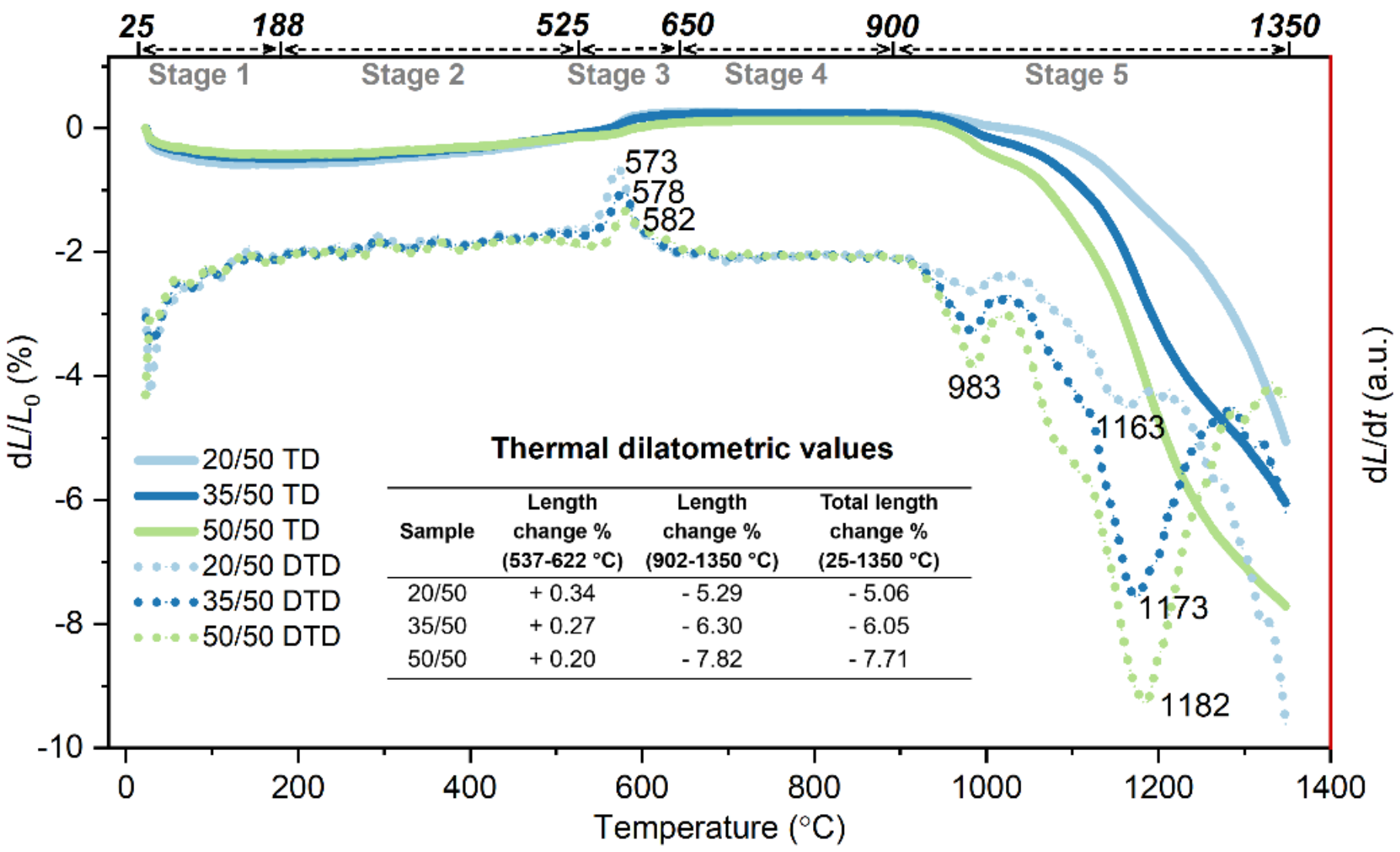

Sample	Length change % (537-622 °C)	Length change % (902-1350 °C)	Total length change % (25-1350 °C)
20/50	+ 0.34	- 5.29	- 5.06
35/50	+ 0.27	- 6.30	- 6.05
50/50	+ 0.20	- 7.82	- 7.71

Supplementary Fig. 6 | Thermal dilatometry and the first derivative (d*L*/d*t*) curves of 20/50, 35/50, and 50/50 clay mixes. Arrows above the curves indicate the five thermal stages between 25 and 1350°C. Inset table shows the length change values in different temperature ranges. Source data are provided as a Source Data file.

Supplementary Table 2. Elemental compositions of raw materials (wt.%).

	SiO_2	Al_2O_3	Fe_2O_3	TiO_2	K_2O	CaO	Na_2O	MgO	Mn_2O_3	P_2O_5	LOI[a)]
Kln	45.01	37.27	0.97	0.76	0.18	0.14	0.08	0.25	0.04	0.08	14.81
Ilt	48.88	32.95	0.43	0.52	9.58	0	0.10	1.11	0.04	0.17	5.78
Hp	75.38	10.03	6.78	0.60	1.34	0.31	0.33	0.51	0.12	0.07	3.79

[a)] LOI- Loss on ignition

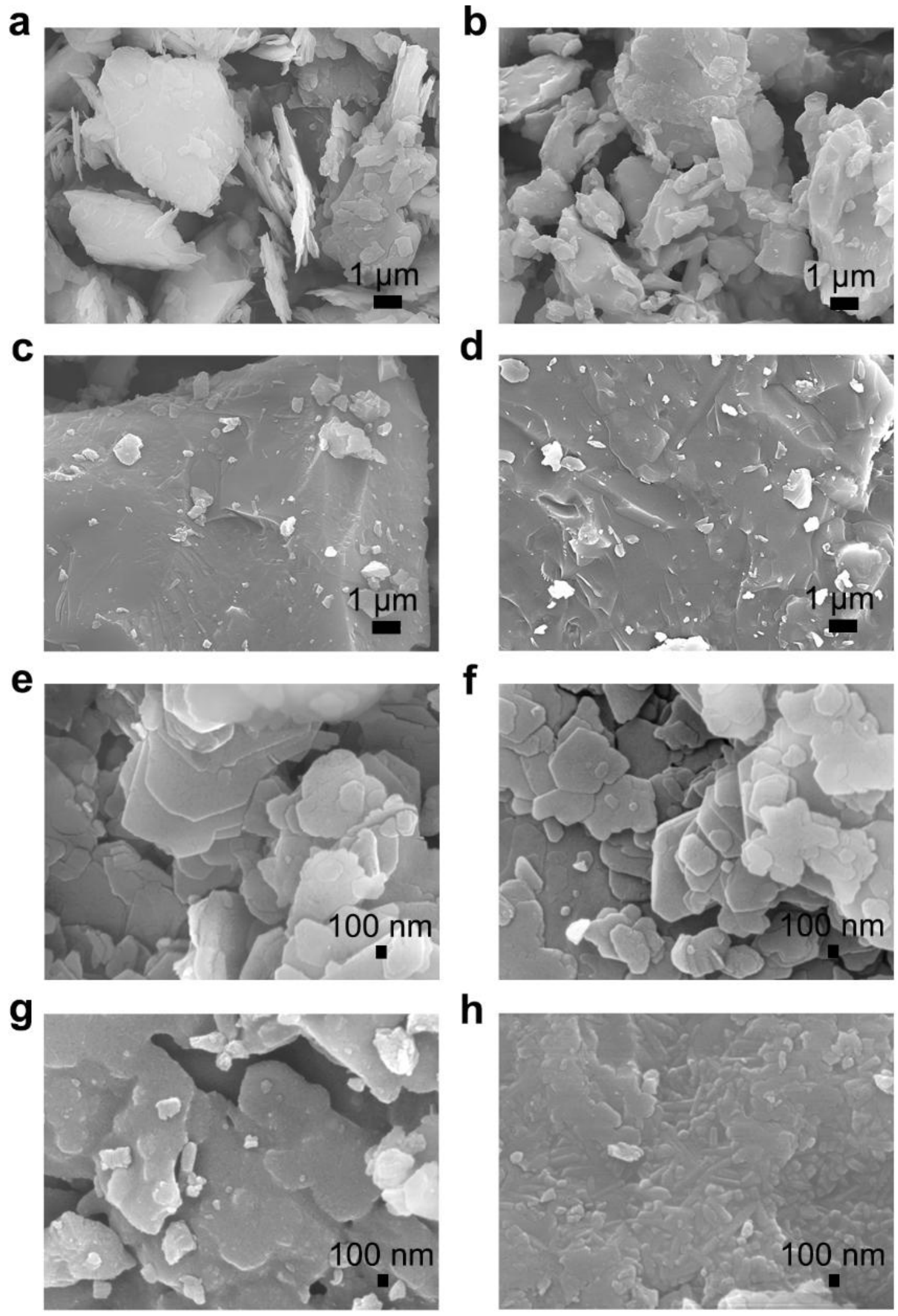

Supplementary Fig. 7 | Morphological evolution of Ilt and Kln during firing. Scanning electron microscopy images of Ilt fired at **a** 900°C, **b** 1050°C, **c** 1200°C and **d** 1350°C and Kln fired at **e** 900°C, **f** 1050°C, **g** 1200°C and **h** 1350°C. Kln, kaolinite; Ilt, illite.

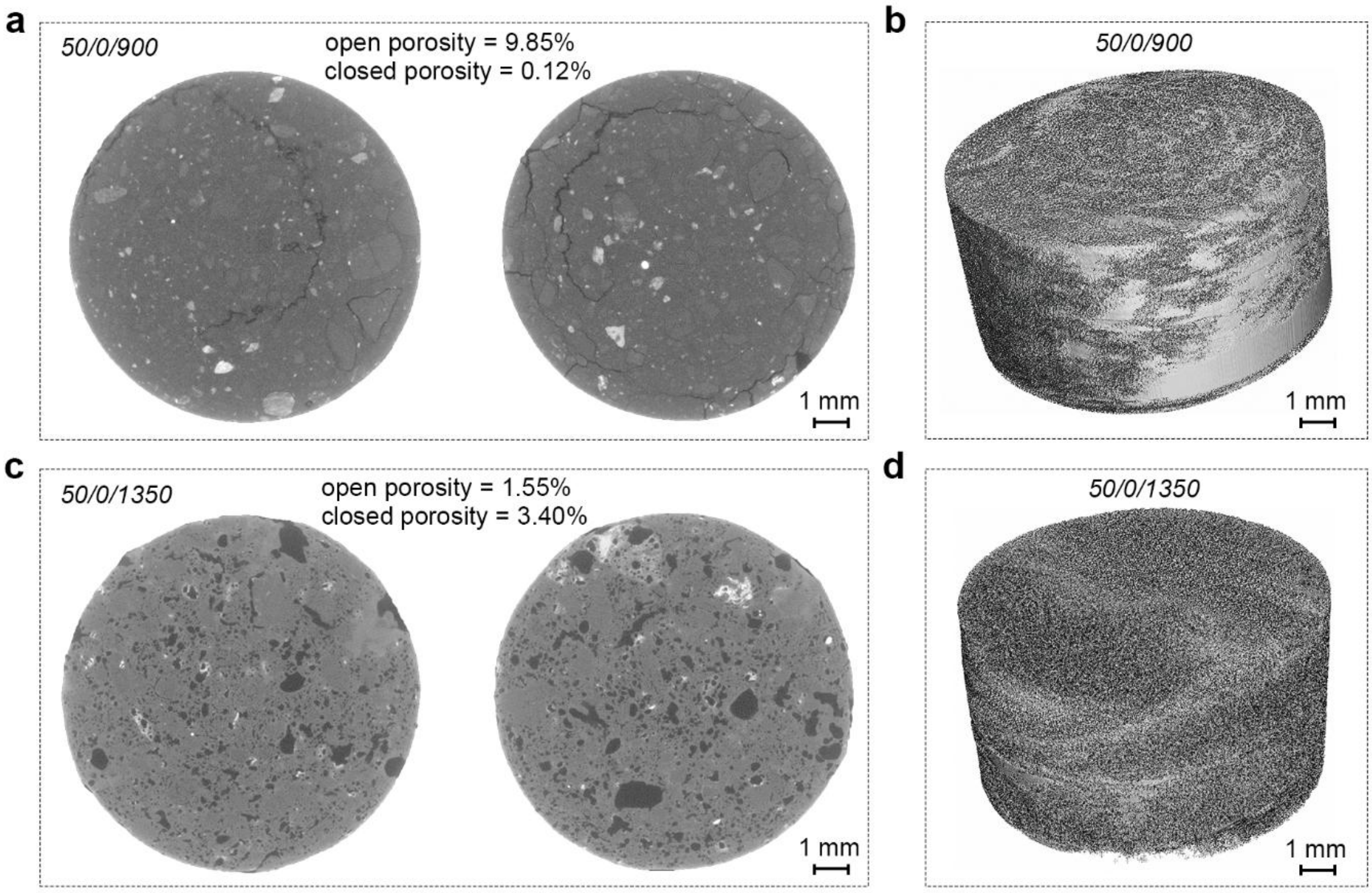

Supplementary Fig. 8 | Micro-CT characterization of pore structures in the 50/0/900 and 50/0/1350 bricks. a, b Representative two-dimensional slice **a** and complete three-dimensional reconstruction **b** of the 50/0/900 brick. **c, d** Representative 2D slice **c** and complete 3D reconstruction **d** of the 50/0/1350 brick. In total, 780 and 730 slices were acquired for the 50/0/900 and 50/0/1350 bricks, respectively. Dark regions represent pores, white regions represent mineral grains and grey regions represent the matrix. Closed porosity increases from 50/0/900 to 50/0/1350.

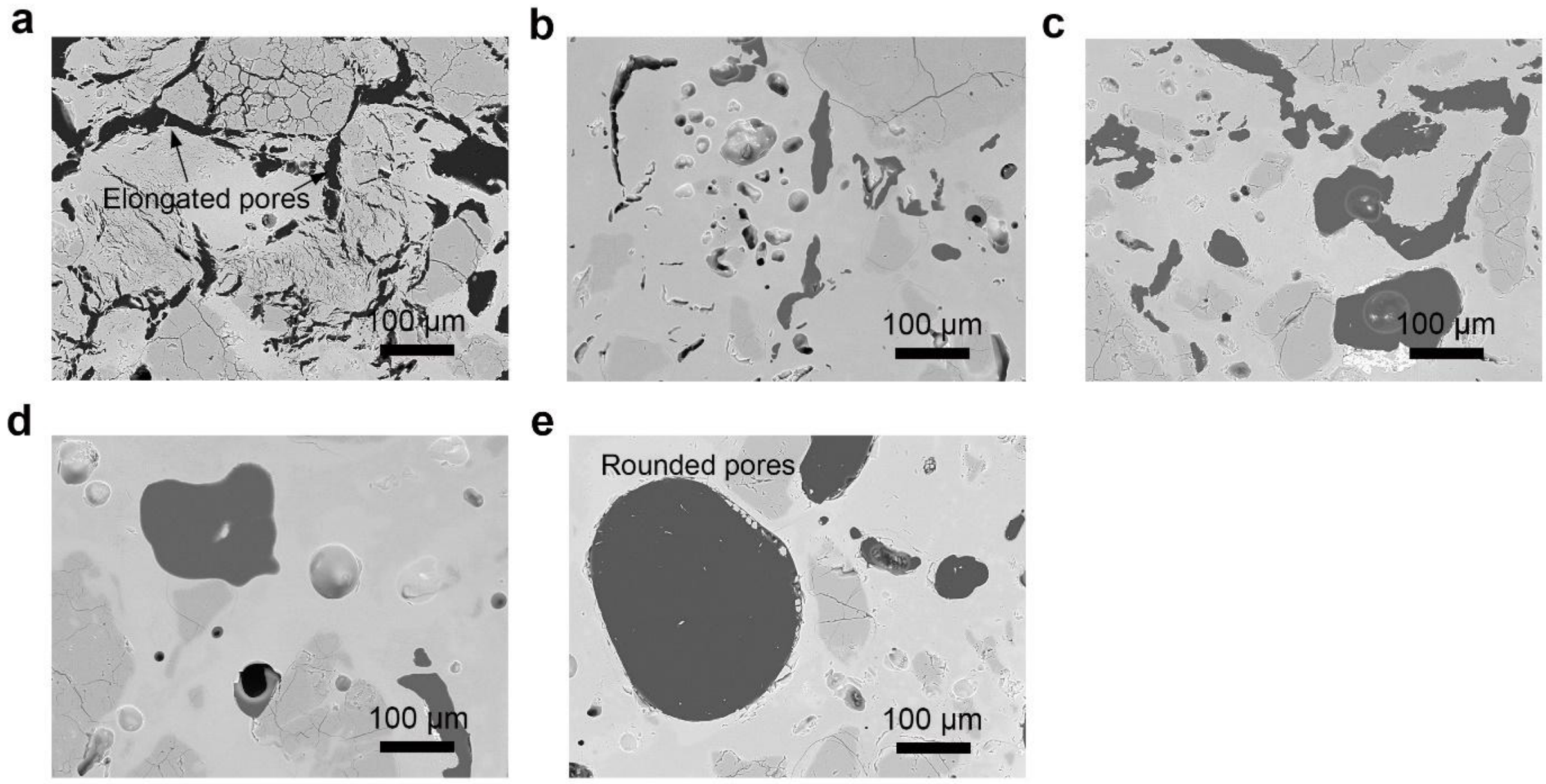

Supplementary Fig. 9 | Microstructures of different clay formulations fired at 1350°C. a Backscattered electron (BSE) images of the 35/100 brick. **b** BSE images of the 35/66 brick. **c** BSE images of the 35/50 brick. **d** BSE images of the 35/33 brick. **e** BSE images of the 35/0 brick.

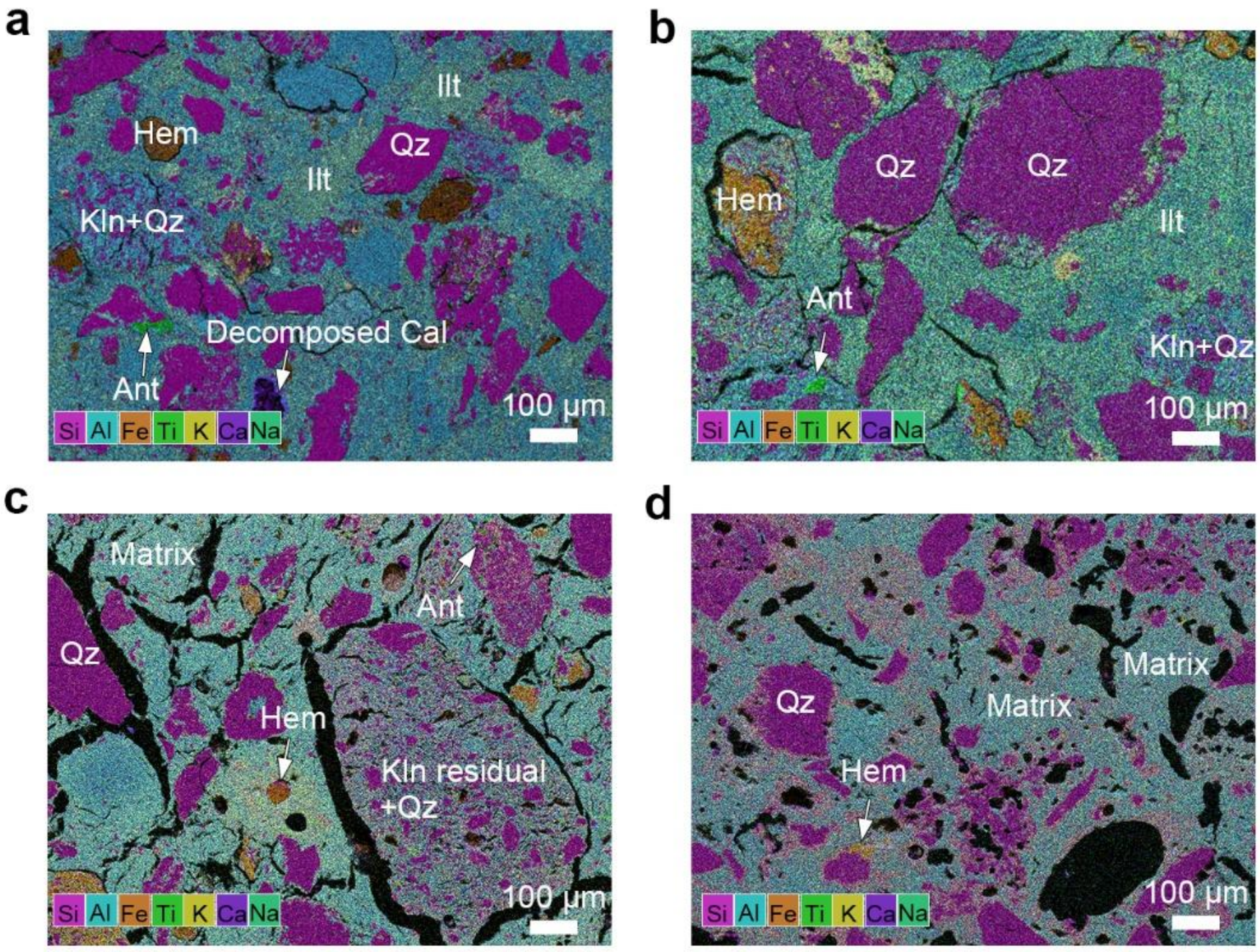

Supplementary Fig. 10 | Elemental distributions and microstructural evolution of the 35/50 formulation after firing. a-d Energy-dispersive X-ray spectroscopy elemental maps of the 35/50 formulation fired at **a** 900°C, **b** 1050°C, **c** 1200°C and **d** 1350°C. Unvitrified Ilt- and Kln-derived particles are present at 900°C and 1050°C. At 1200°C, the blurred particle boundaries indicate extensive vitrification of the Ilt-derived regions, associated with the fluxing elements in Ilt, whereas the Kln-derived regions remain largely unvitrified. At 1350°C, a homogeneous matrix forms, accompanied by uniform distributions of Si, Al and K in **d**. Kln, kaolinite; Ilt, illite; Hem, hematite; Qz, quartz; Ant, anatase; Cal, calcite.

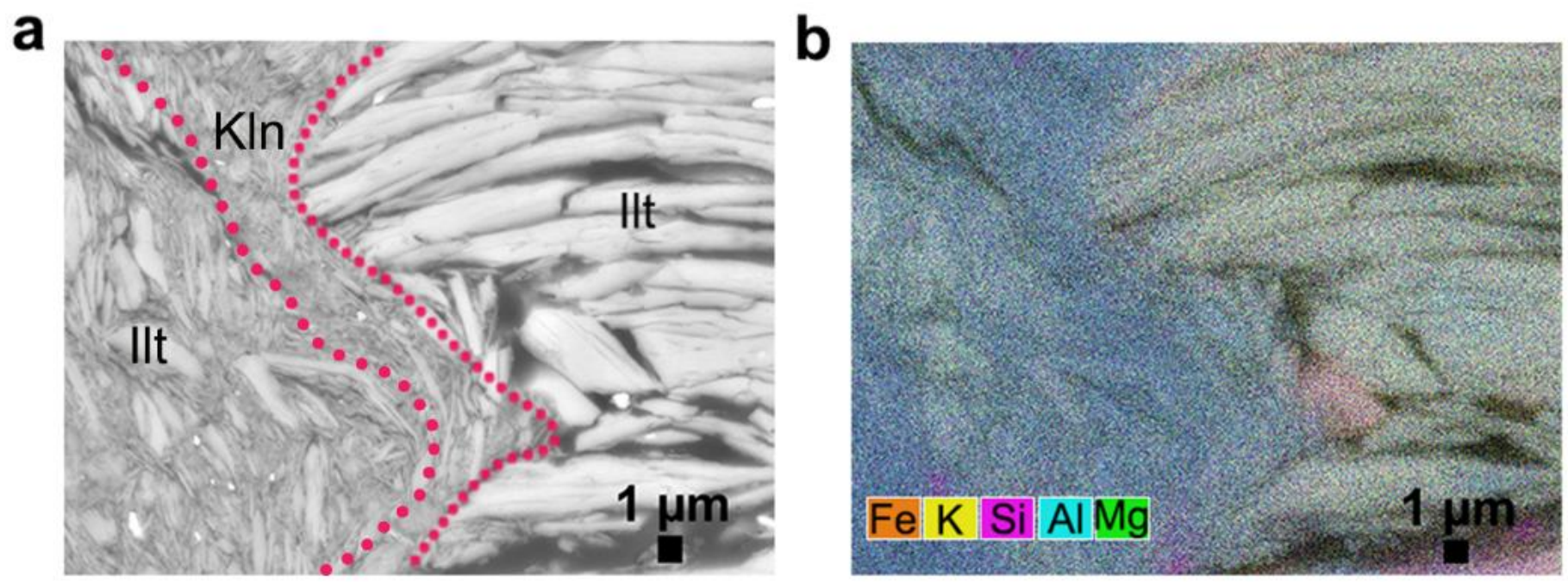

Supplementary Fig. 11 | Contact relationship between Kln- and Ilt- -derived regions after firing at 900°C. a BSE image showing the contact between Kln- and Ilt-derived particles. Dashed lines indicate the interface between the two regions. **b** Corresponding energy-dispersive X-ray spectroscopy elemental maps. Kln, kaolinite; Ilt, illite.

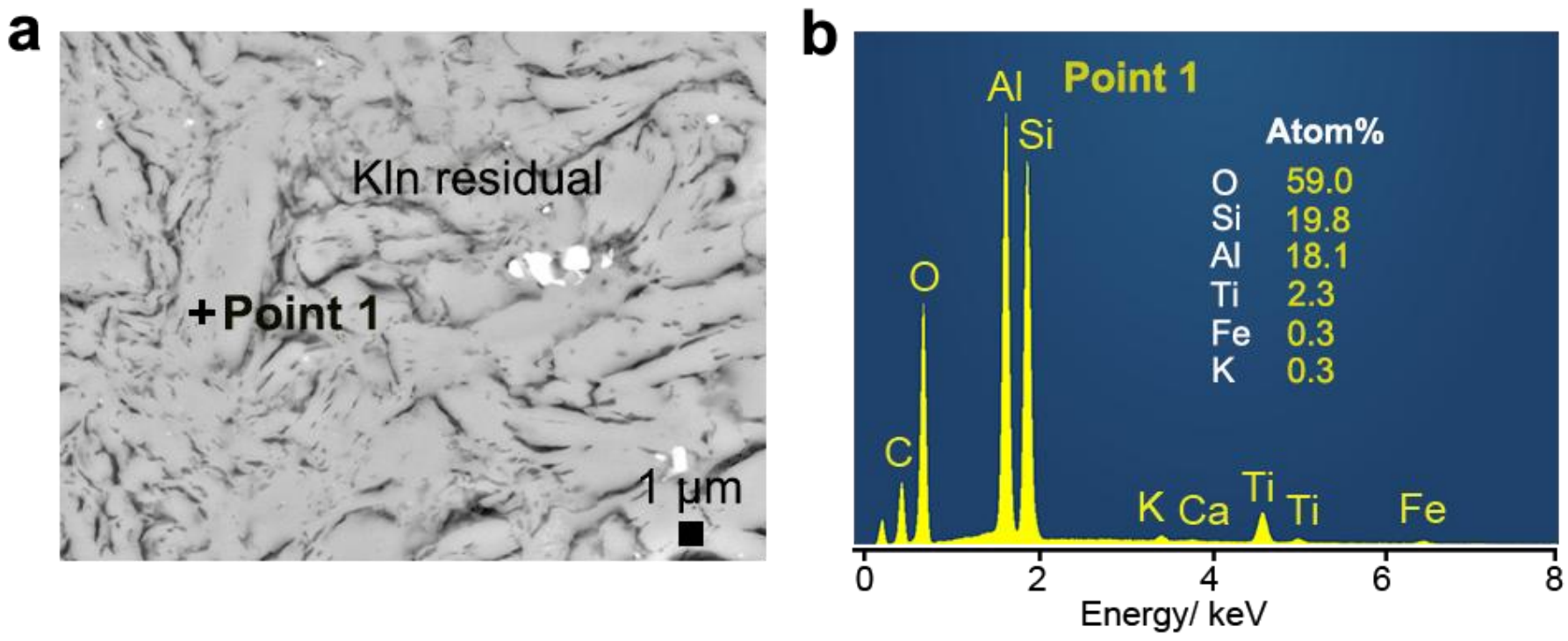

Supplementary Fig. 12 | Partial vitrification of Kln after firing at 1200 °C. a BSE image showing the partial vitrification of Kln-derived particles. **b** Corresponding energy-dispersive X-ray spectroscopy analysis. Kln, kaolinite.

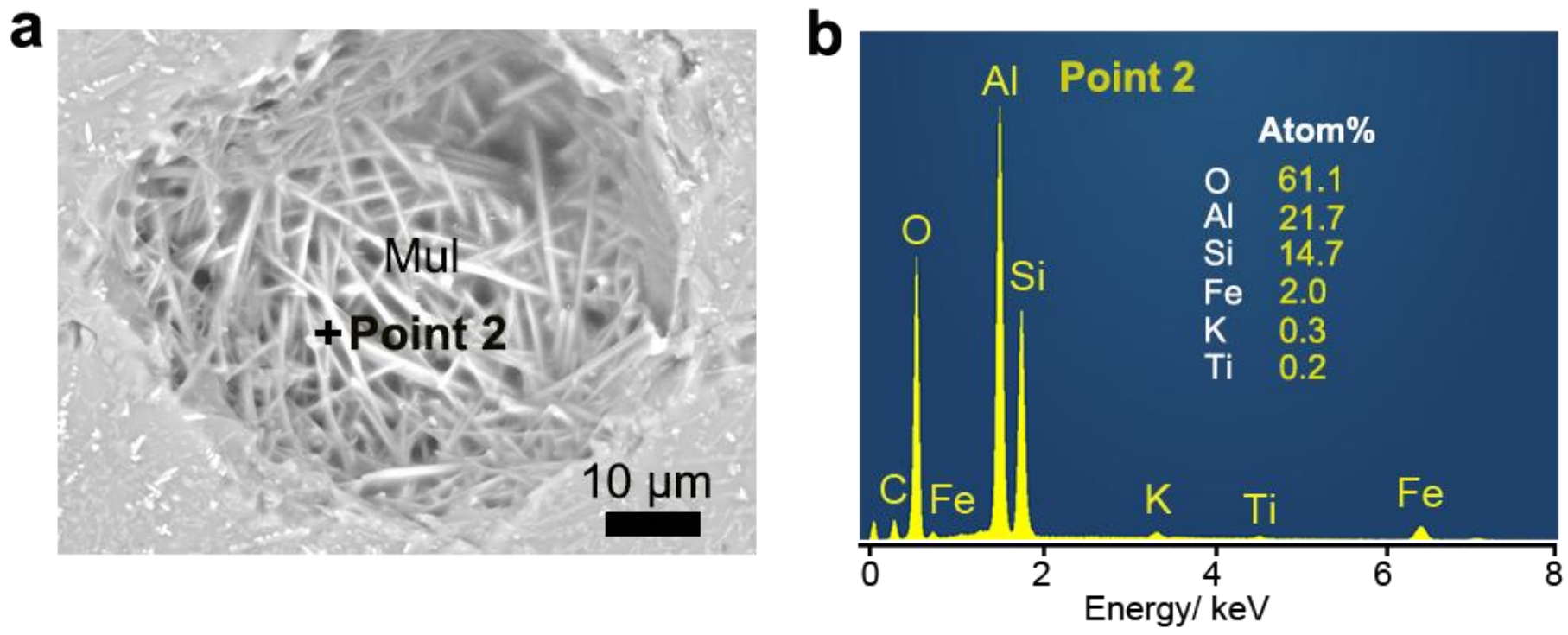

Supplementary Fig. 13 | Formation of mullite needles after firing at 1350 °C. a BSE image showing mullite (Mul) needles. **b** Corresponding energy-dispersive X-ray spectroscopy analysis.

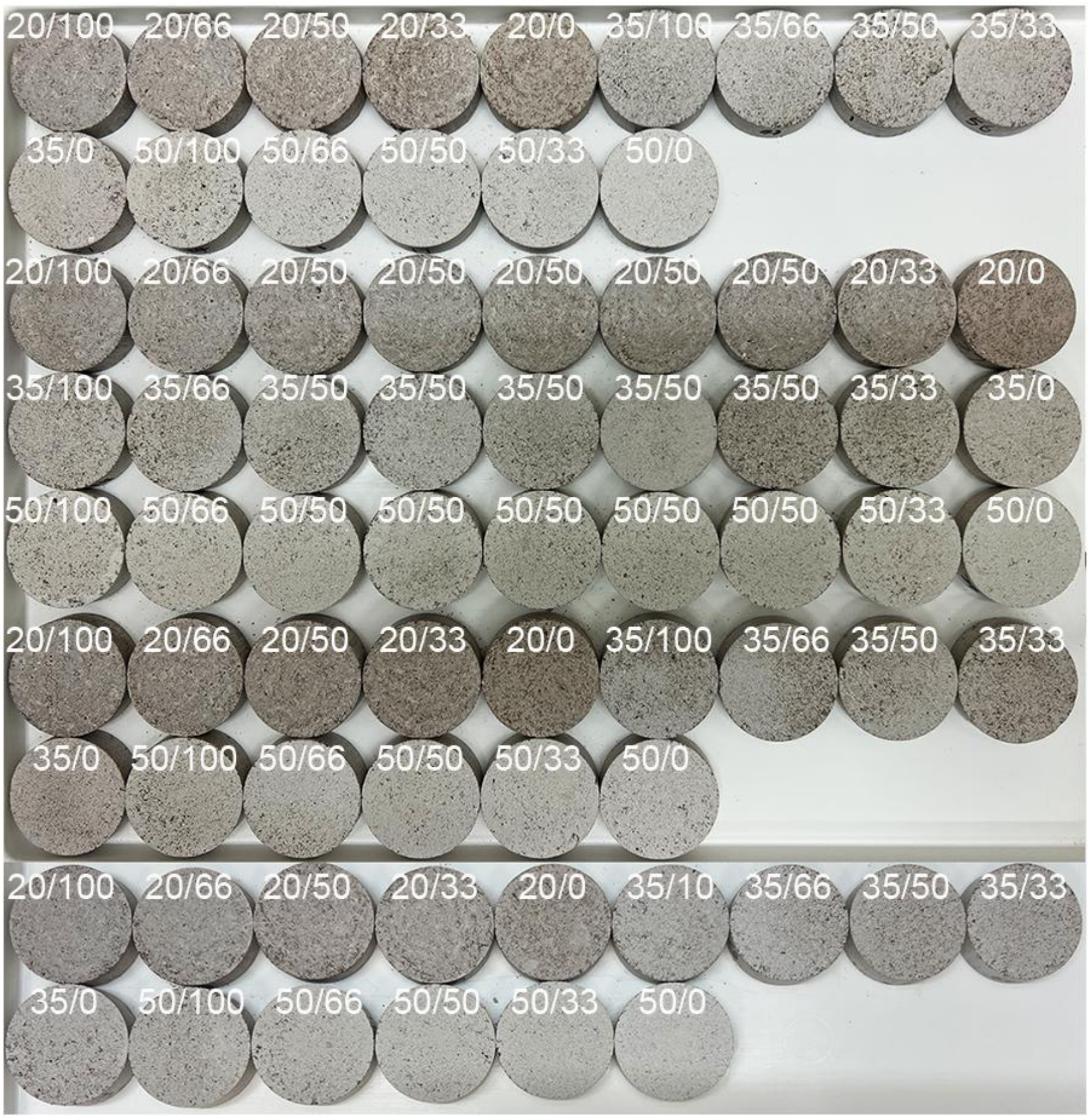

Supplementary Fig. 14 | Photos of 72 dried brick buttons.

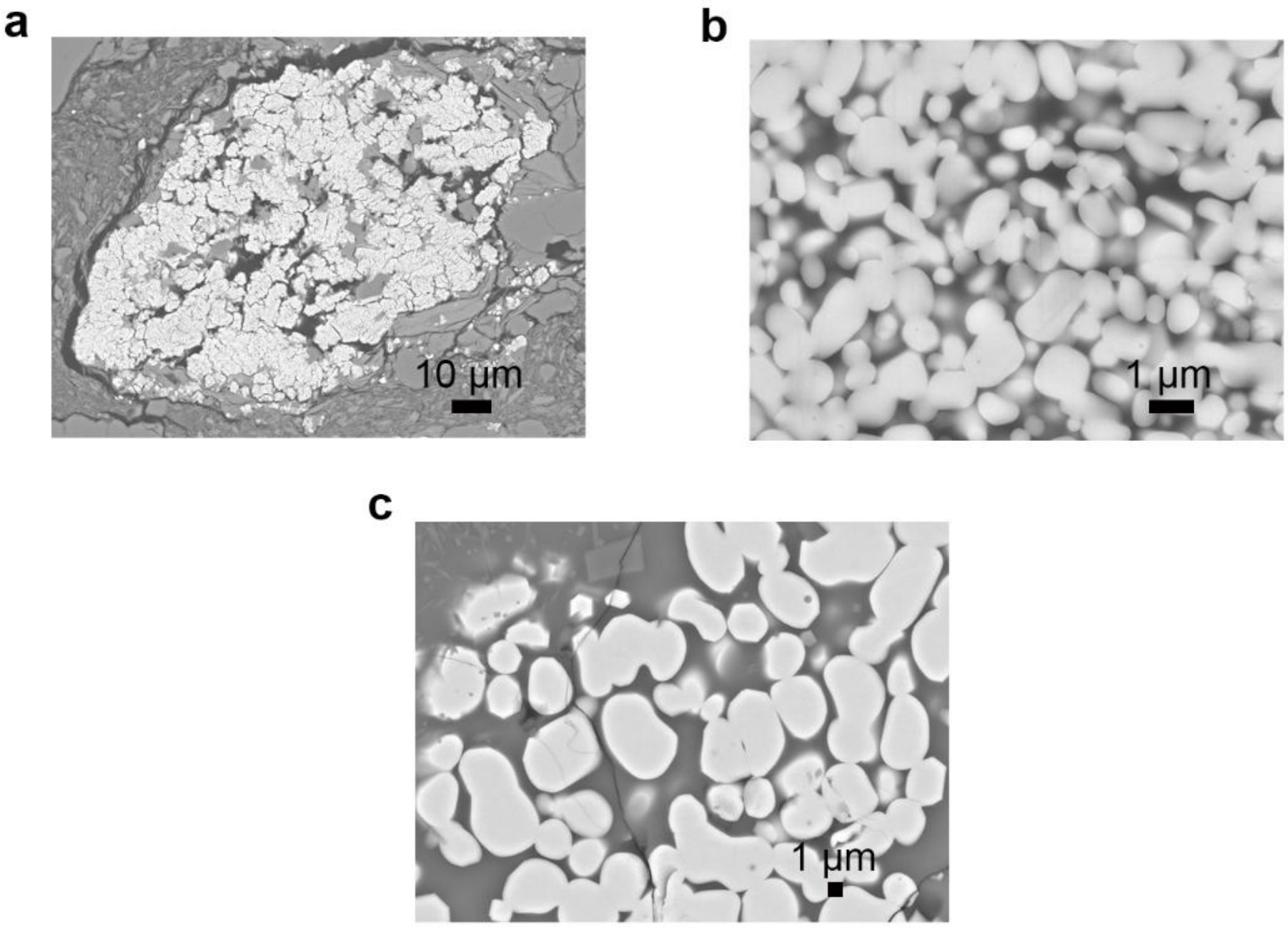

Supplementary Fig. 15 | Growth of hematite crystals during firing. BSE images showing hematite crystals after firing at **a** 900°C, **b** 1200°C and **c** 1350°C.

20/0/900

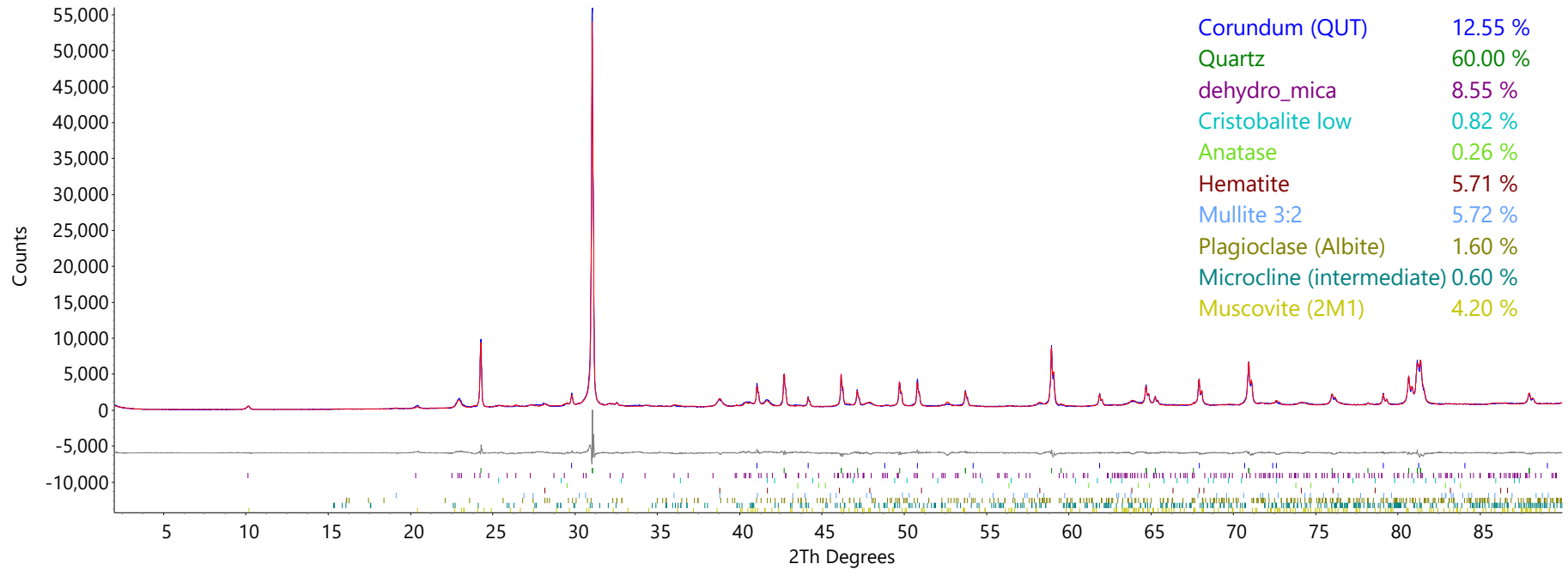

20/0/1050

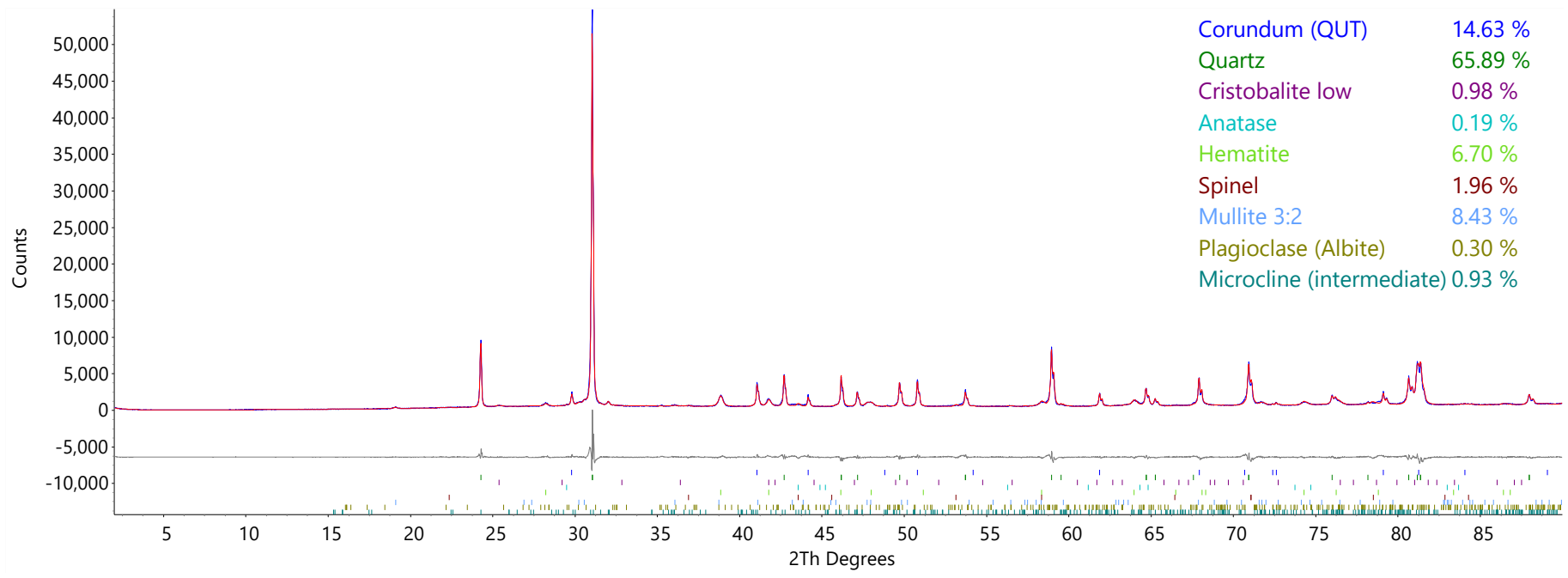

20/0/1200

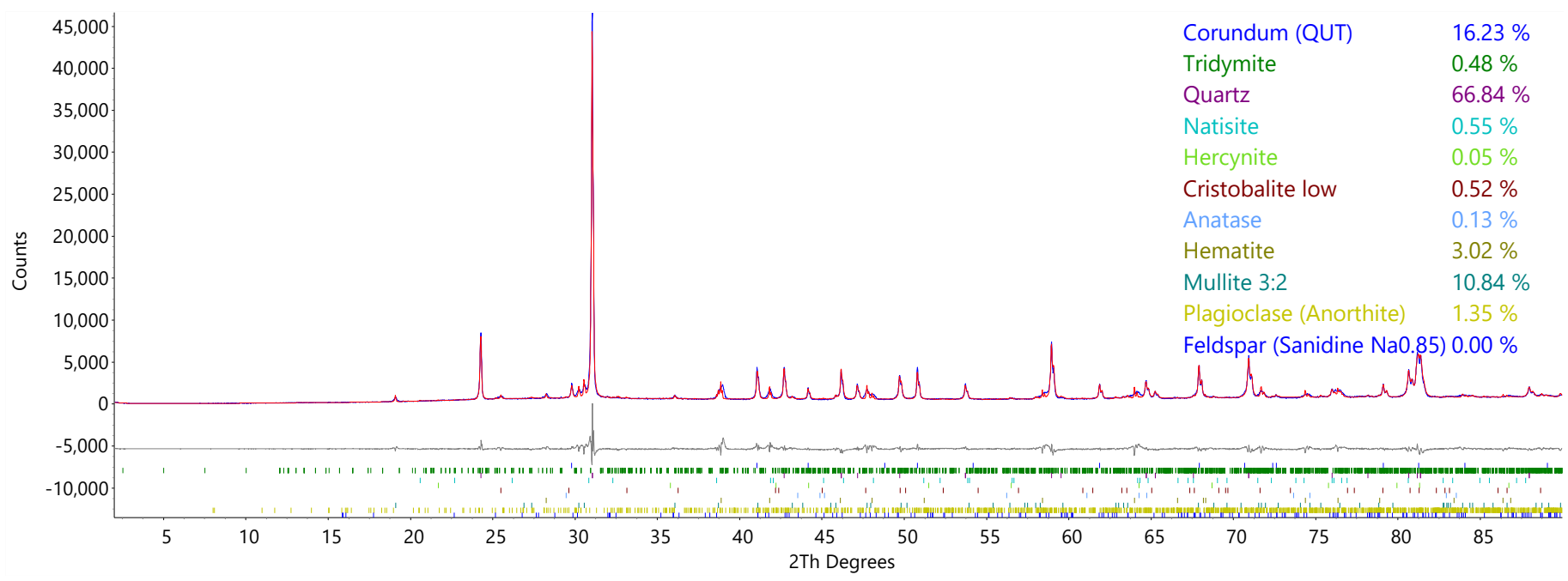

20/0/1350

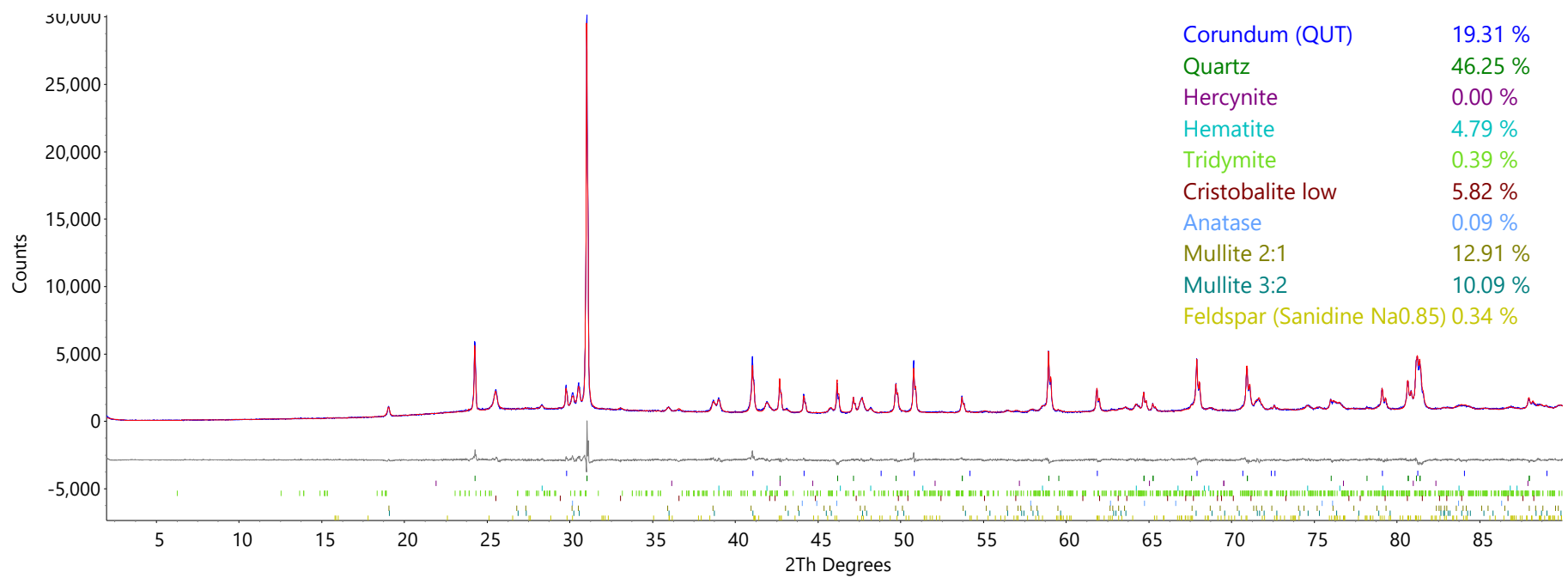

Supplementary Fig. 16 | Raw Rietveld refinement results of X-ray diffraction (XRD) patterns for the 20/0 formulation fired at 900°C, 1050°C, 1200°C, and 1350°C. The red line is the Rietveld refinement model; the blue line is the collected data, and the grey line is the difference. Tick marks correspond to different phases used in the refinement and expected peak positions for those phases. Approximately 10 wt.% corundum was added as an internal standard to enable quantitative determination of the amorphous content in the fired bricks.

20/50/900

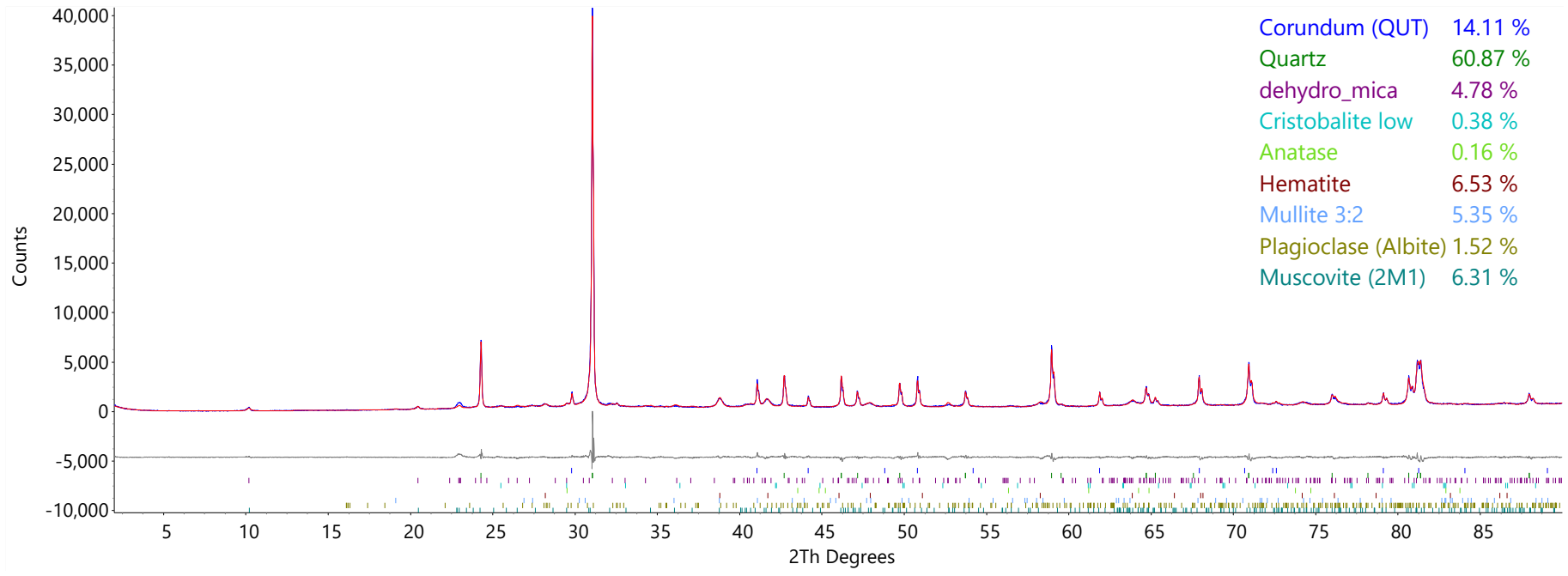

20/50/1050

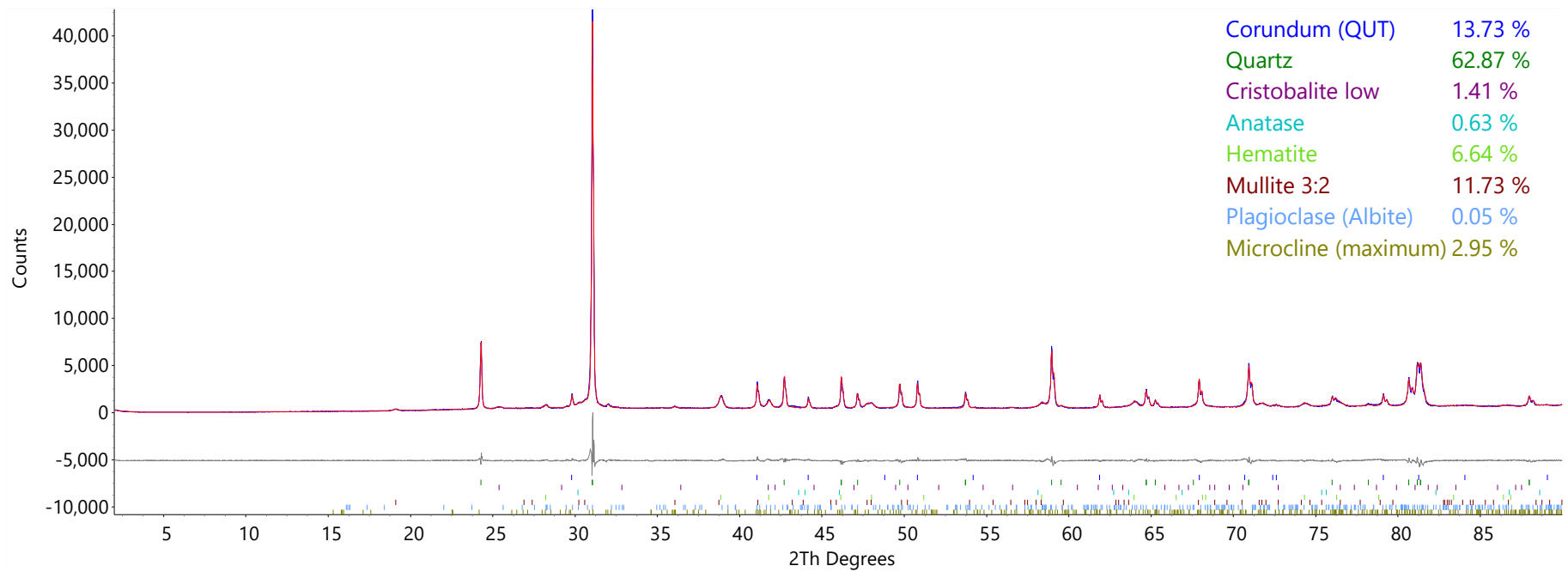

20/50/1200

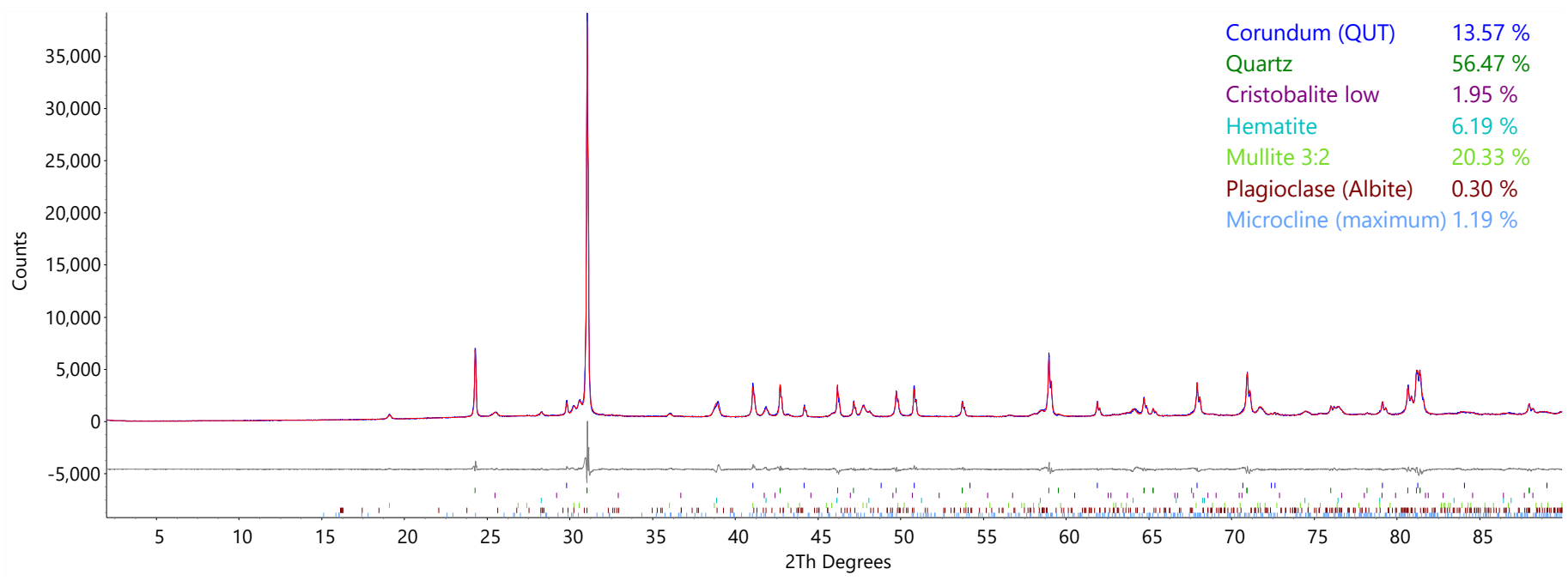

20/50/1350

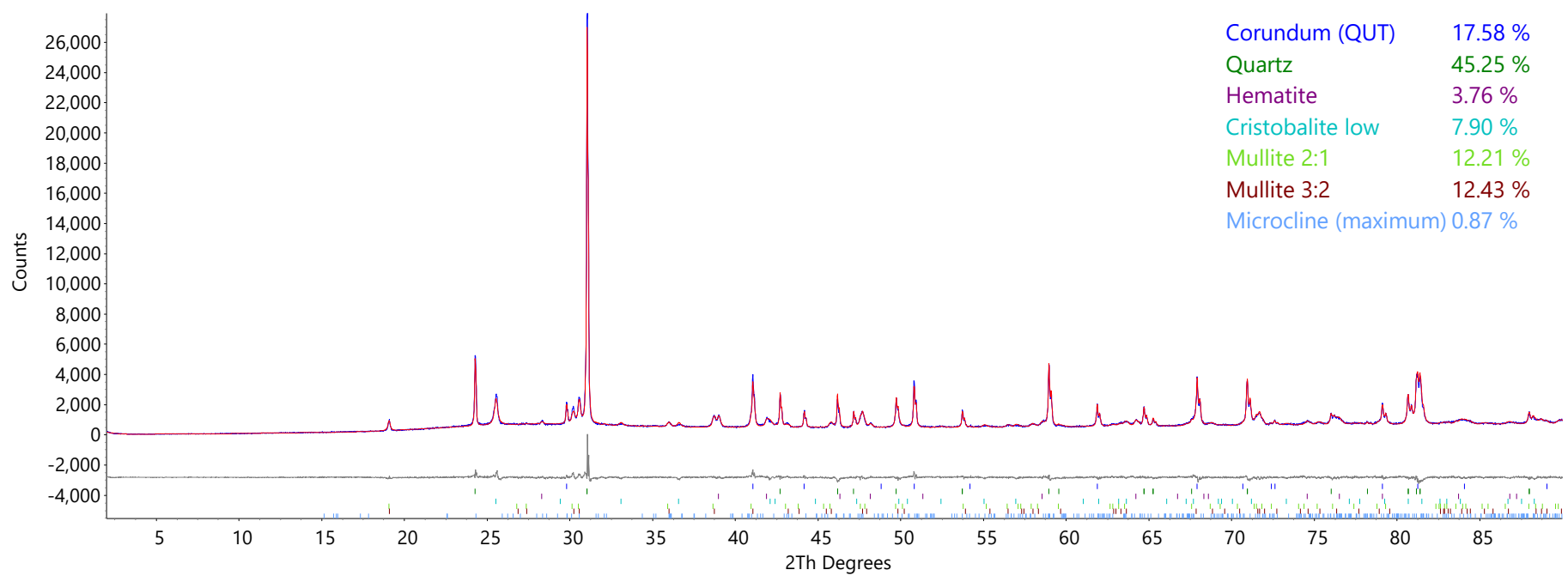

Supplementary Fig. 17 | Raw Rietveld refinement results of X-ray diffraction (XRD) patterns for the 20/50 formulation fired at 900°C, 1050°C, 1200°C, and 1350°C. The red line is the Rietveld refinement model; the blue line is the collected data, and the grey line is the difference. Tick marks correspond to different phases used in the refinement and expected peak positions for those phases. Approximately 10 wt.% corundum was added as an internal standard to enable quantitative determination of the amorphous content in the fired bricks.

20/100/900

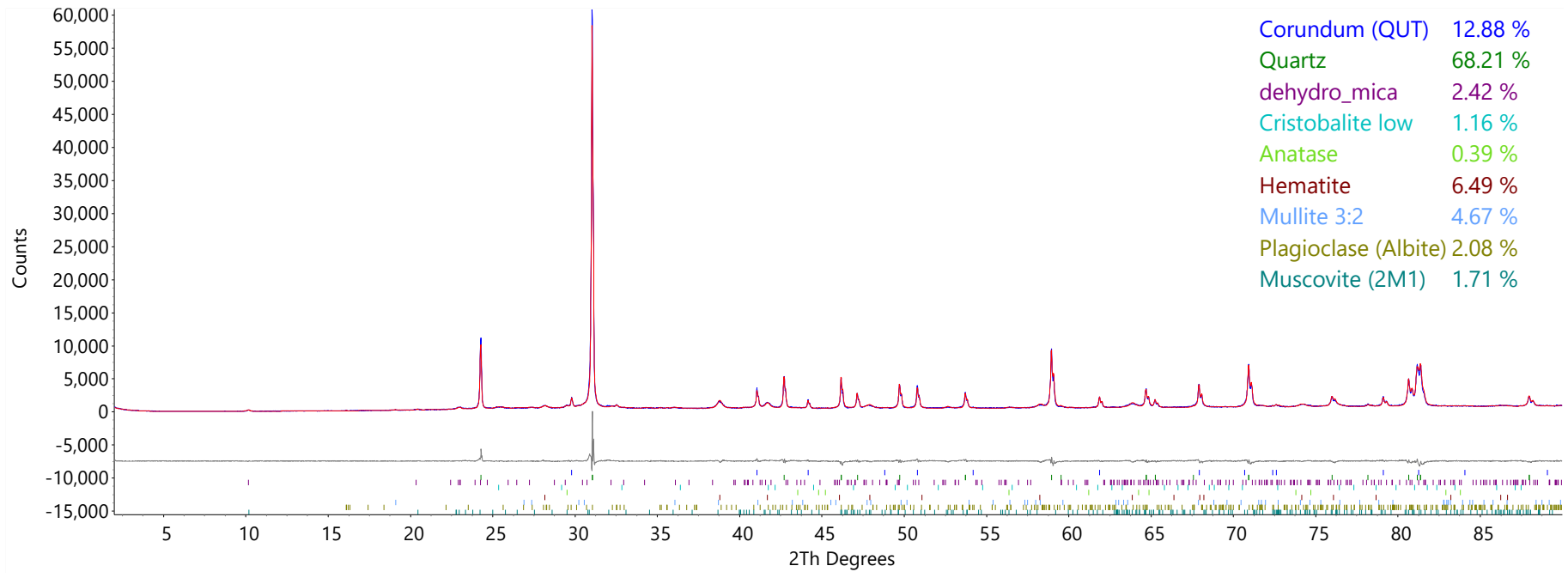

20/100/1050

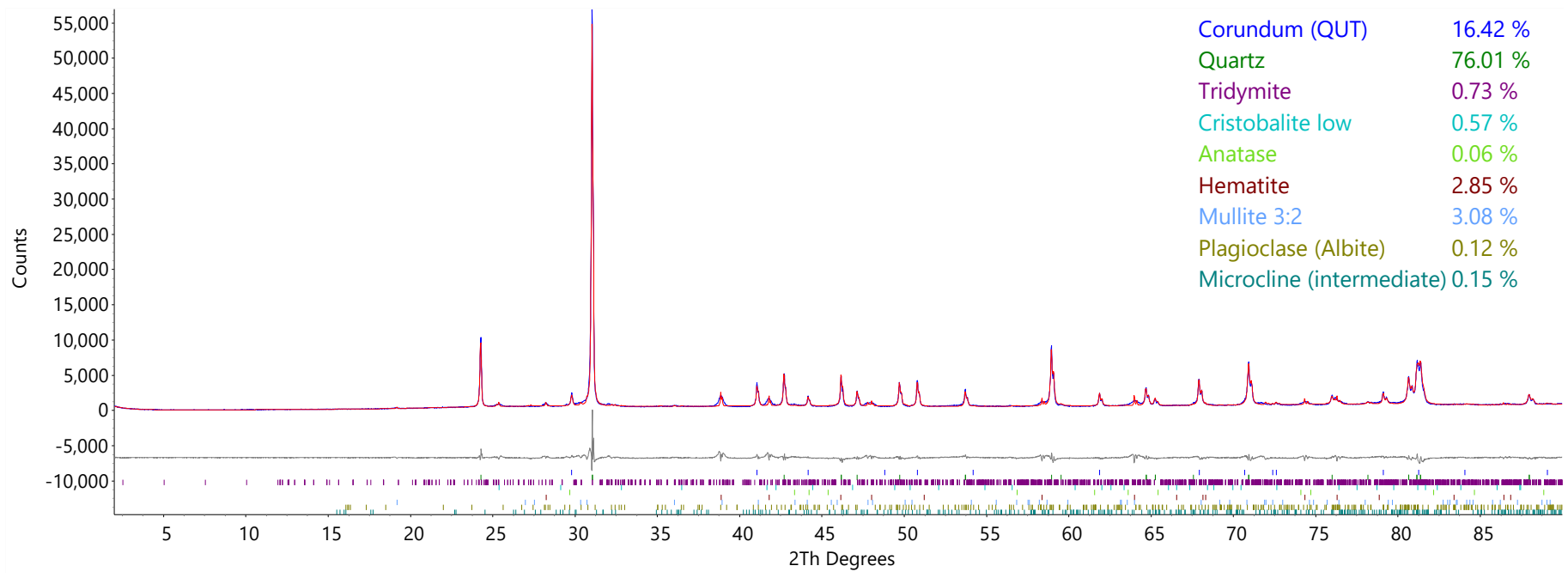

20/100/1200

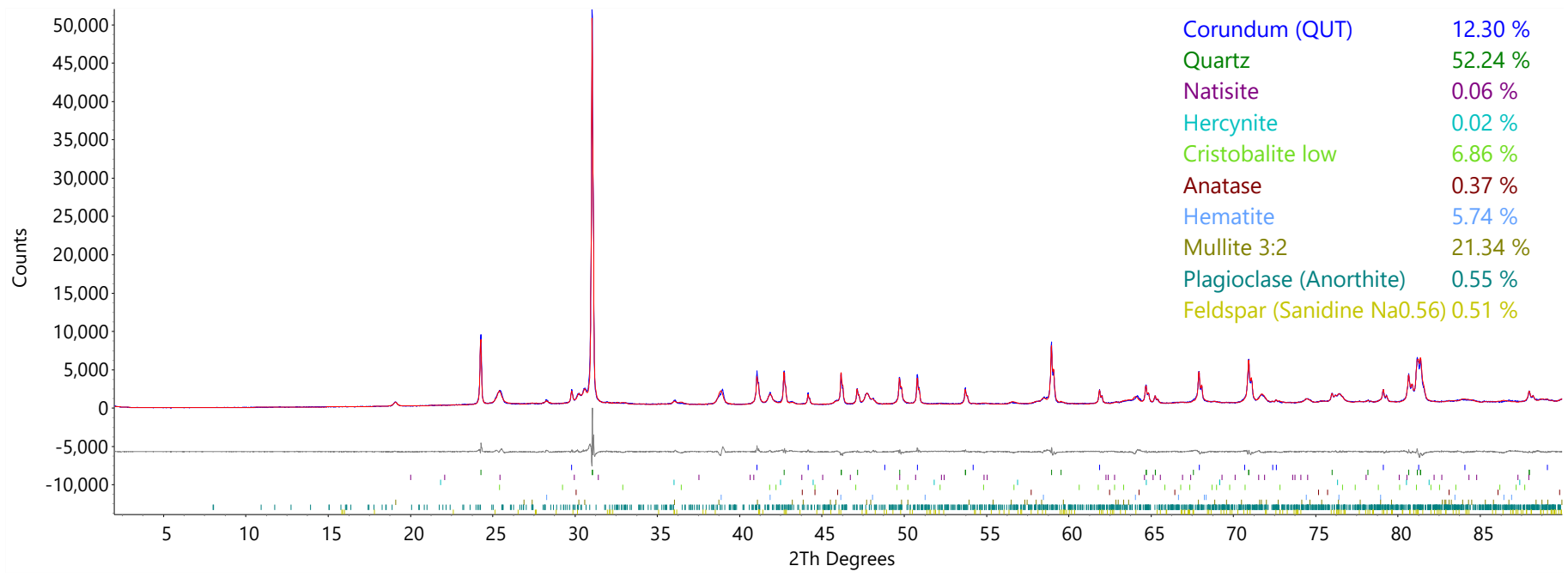

20/100/1350

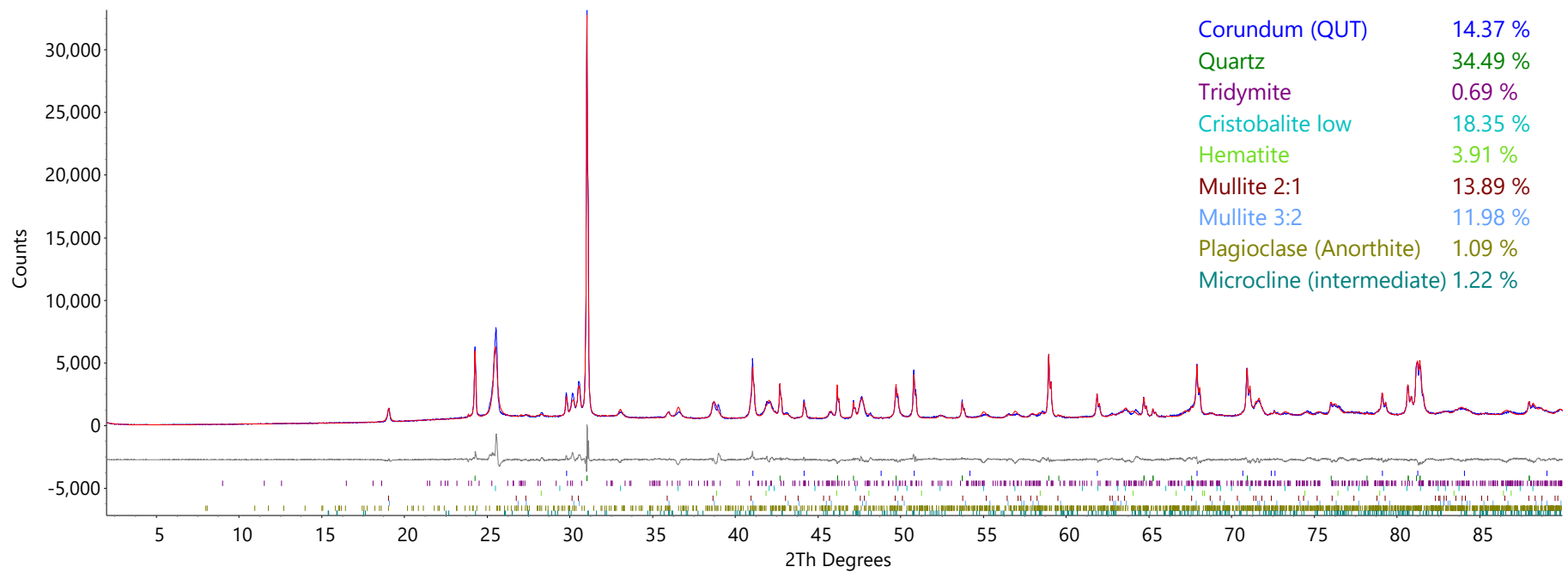

Supplementary Fig. 18 | Raw Rietveld refinement results of X-ray diffraction (XRD) patterns for the 20/100 formulation fired at 900°C, 1050°C, 1200°C, and 1350°C. The red line is the Rietveld refinement model; the blue line is the collected data, and the grey line is the difference. Tick marks correspond to different phases used in the refinement and expected peak positions for those phases. Approximately 10 wt.% corundum was added as an internal standard to enable quantitative determination of the amorphous content in the fired bricks.

35/0/900

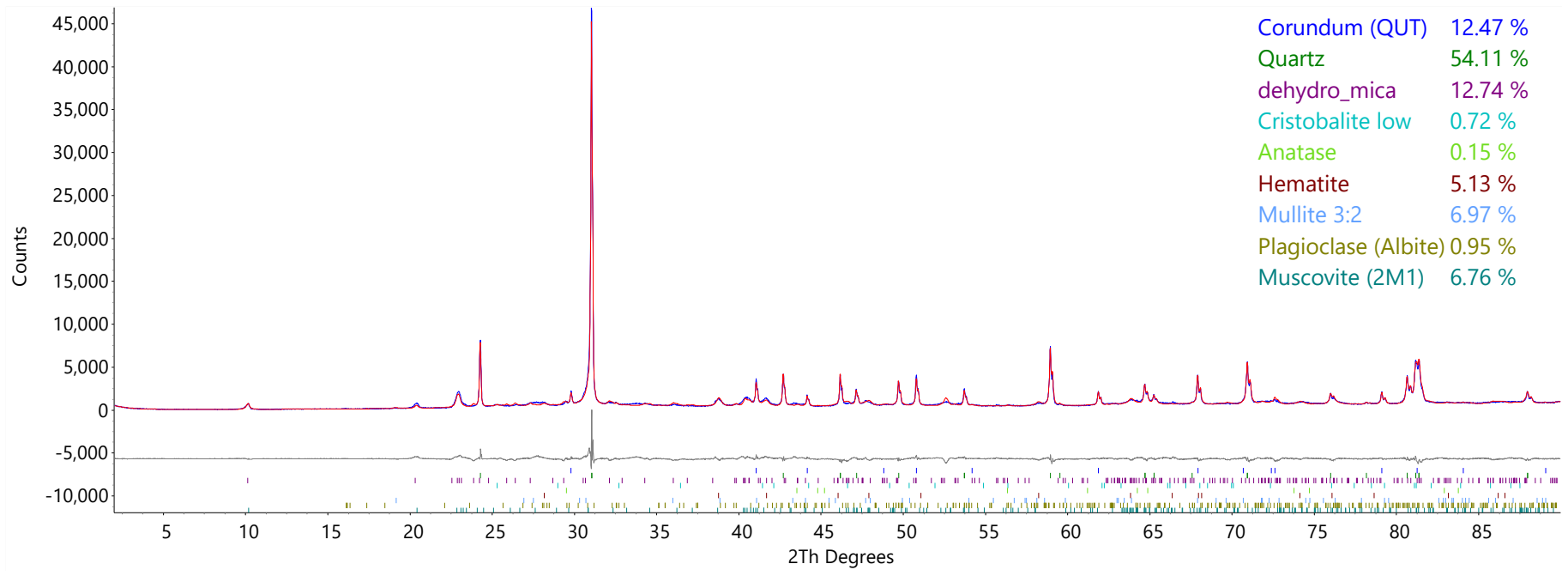

35/0/1050

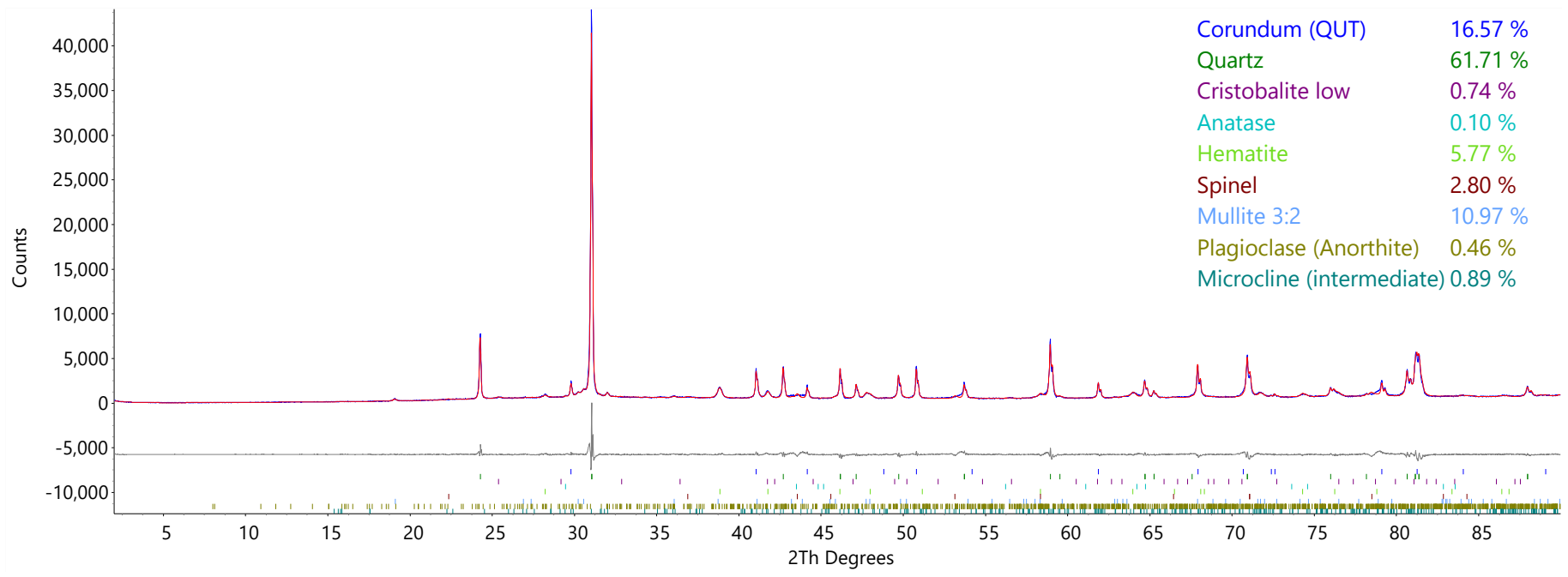

35/0/1200

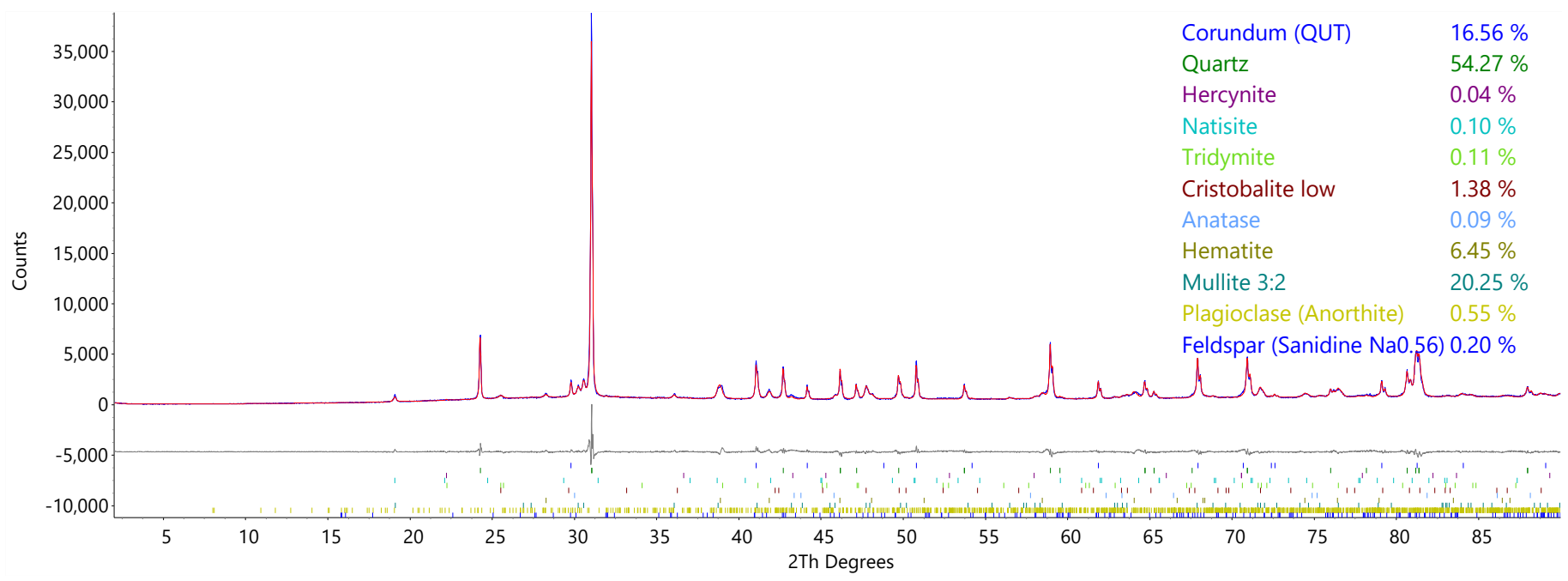

35/0/1350

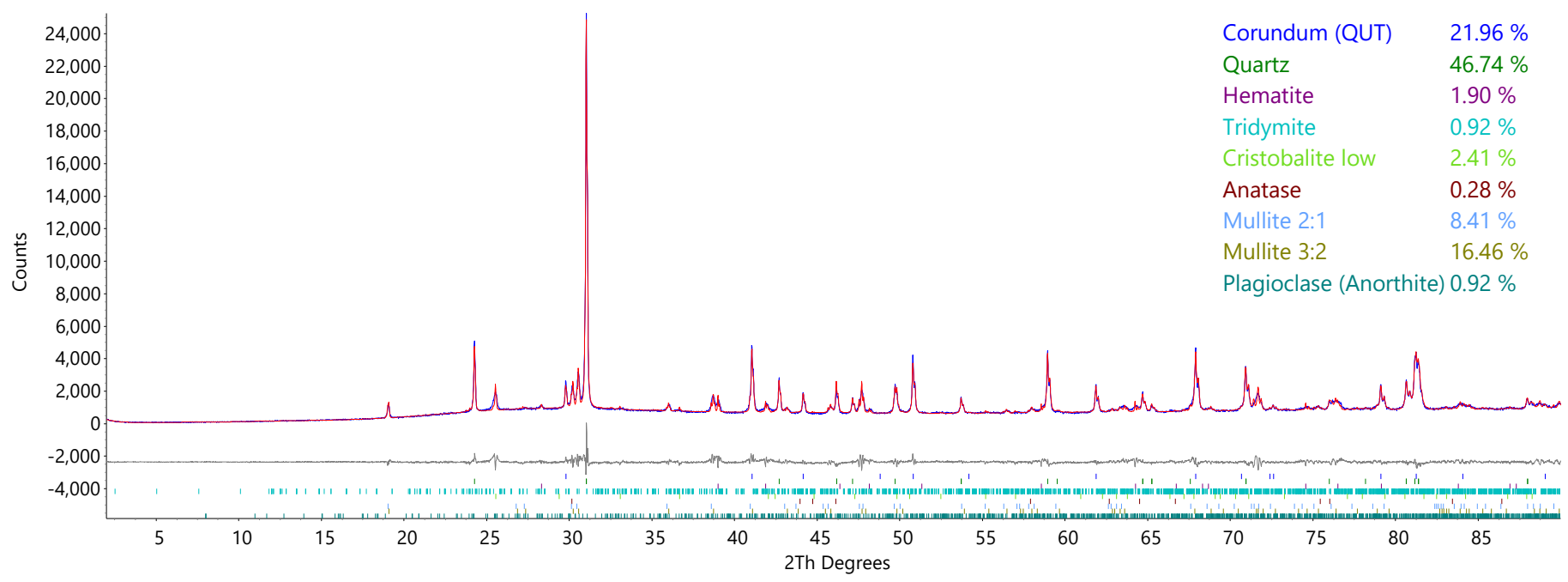

Supplementary Fig. 19 | Raw Rietveld refinement results of X-ray diffraction (XRD) patterns for the 35/0 formulation fired at 900°C, 1050°C, 1200°C, and 1350°C. The red line is the Rietveld refinement model; the blue line is the collected data, and the grey line is the difference. Tick marks correspond to different phases used in the refinement and expected peak positions for those phases. Approximately 10 wt.% corundum was added as an internal standard to enable quantitative determination of the amorphous content in the fired bricks.

35/50/900

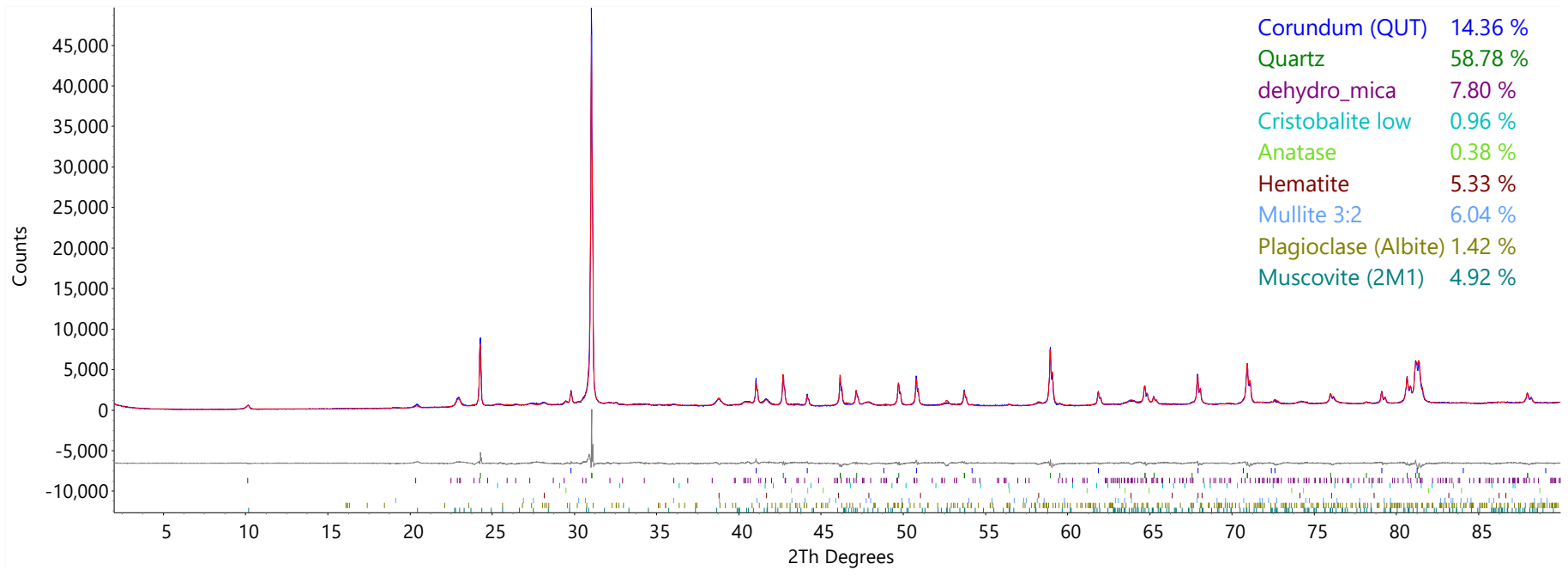

35/50/1050

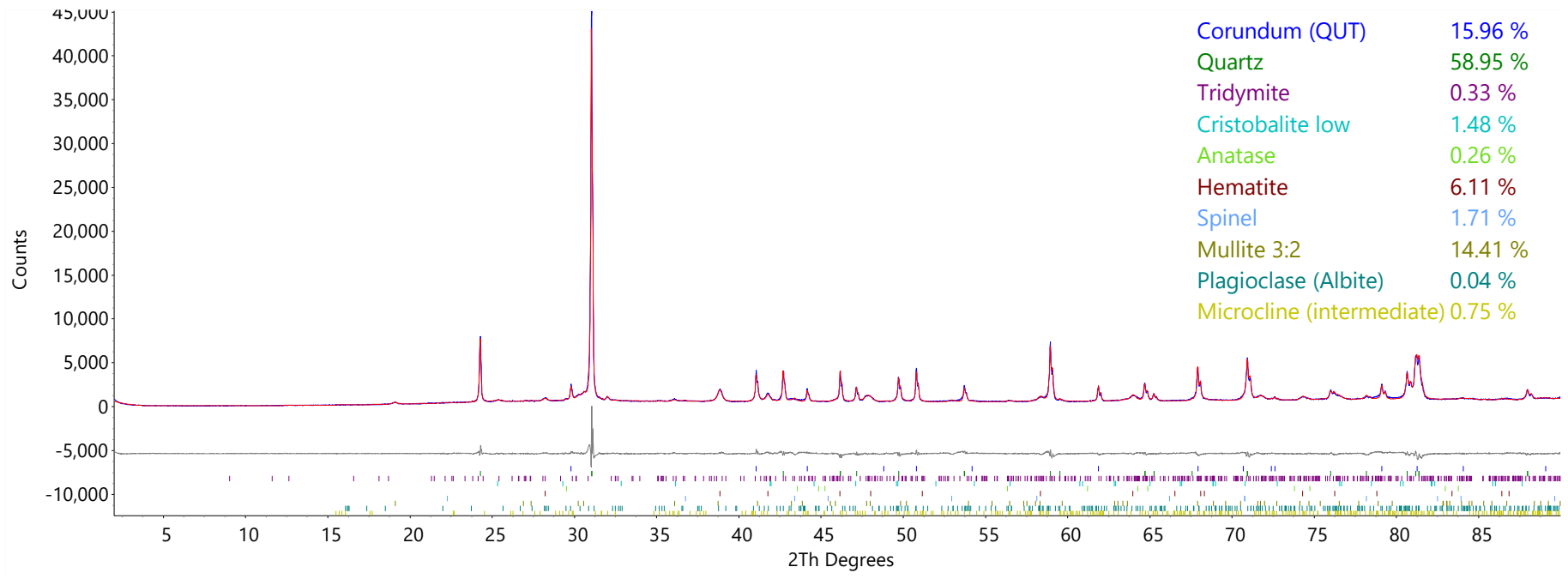

35/50/1200

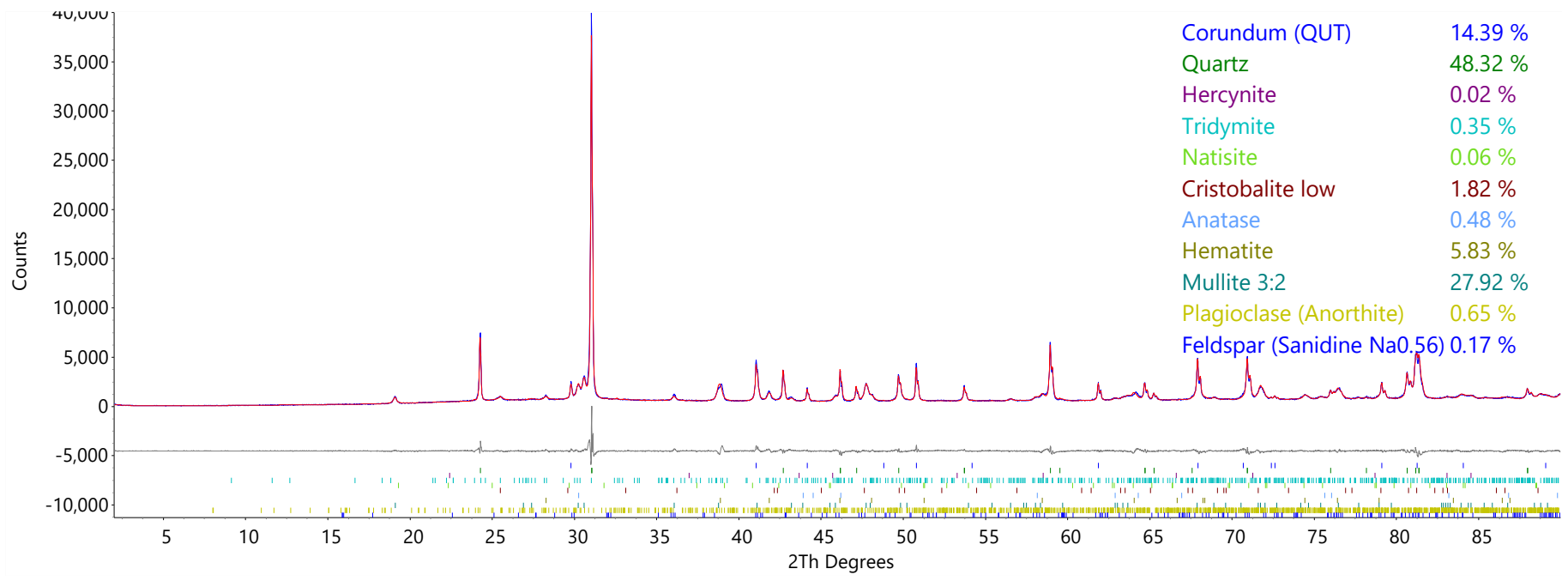

35/50/1350

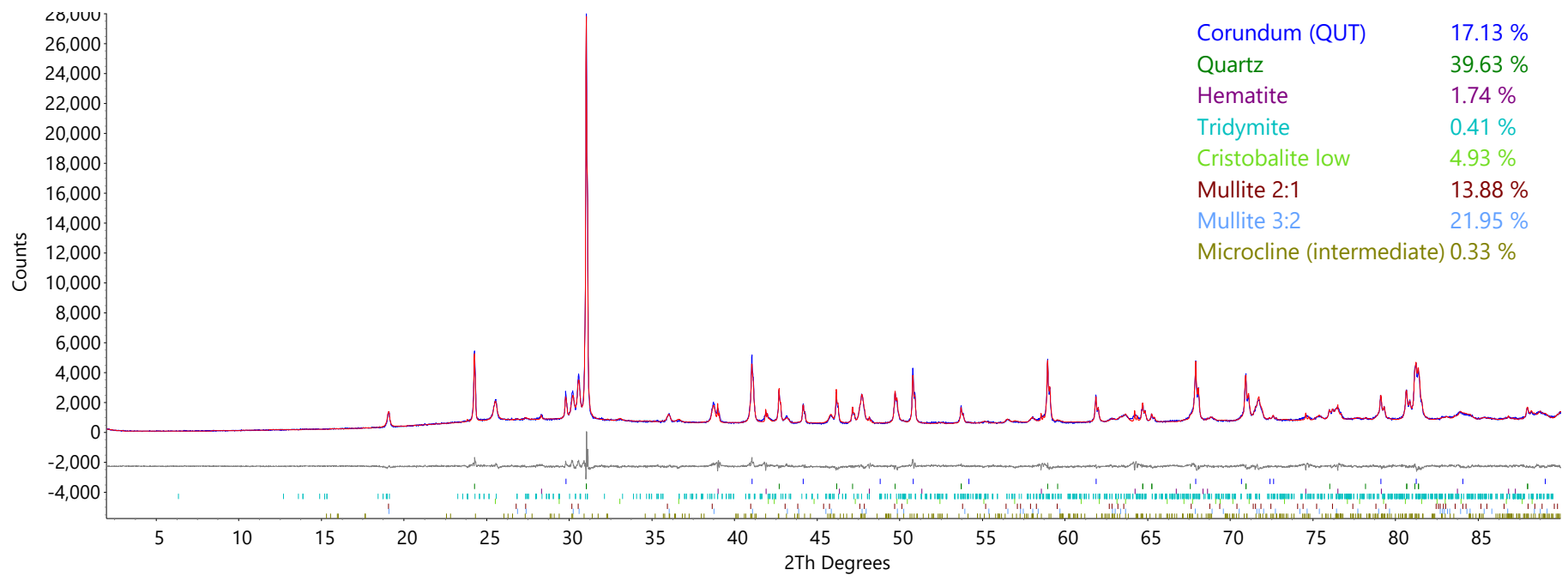

Supplementary Fig. 20 | Raw Rietveld refinement results of X-ray diffraction (XRD) patterns for the 35/50 formulation fired at 900°C, 1050°C, 1200°C, and 1350°C. The red line is the Rietveld refinement model; the blue line is the collected data, and the grey line is the difference. Tick marks correspond to different phases used in the refinement and expected peak positions for those phases. Approximately 10 wt.% corundum was added as an internal standard to enable quantitative determination of the amorphous content in the fired bricks.

35/100/900

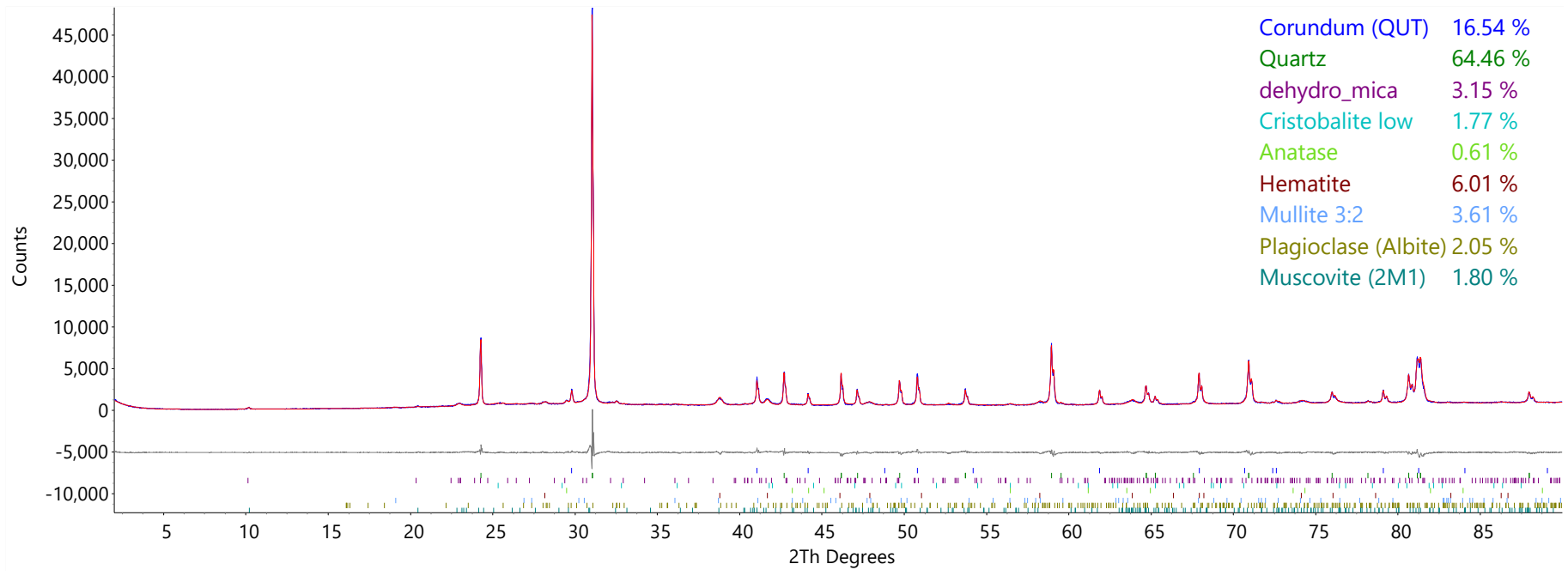

35/100/1050

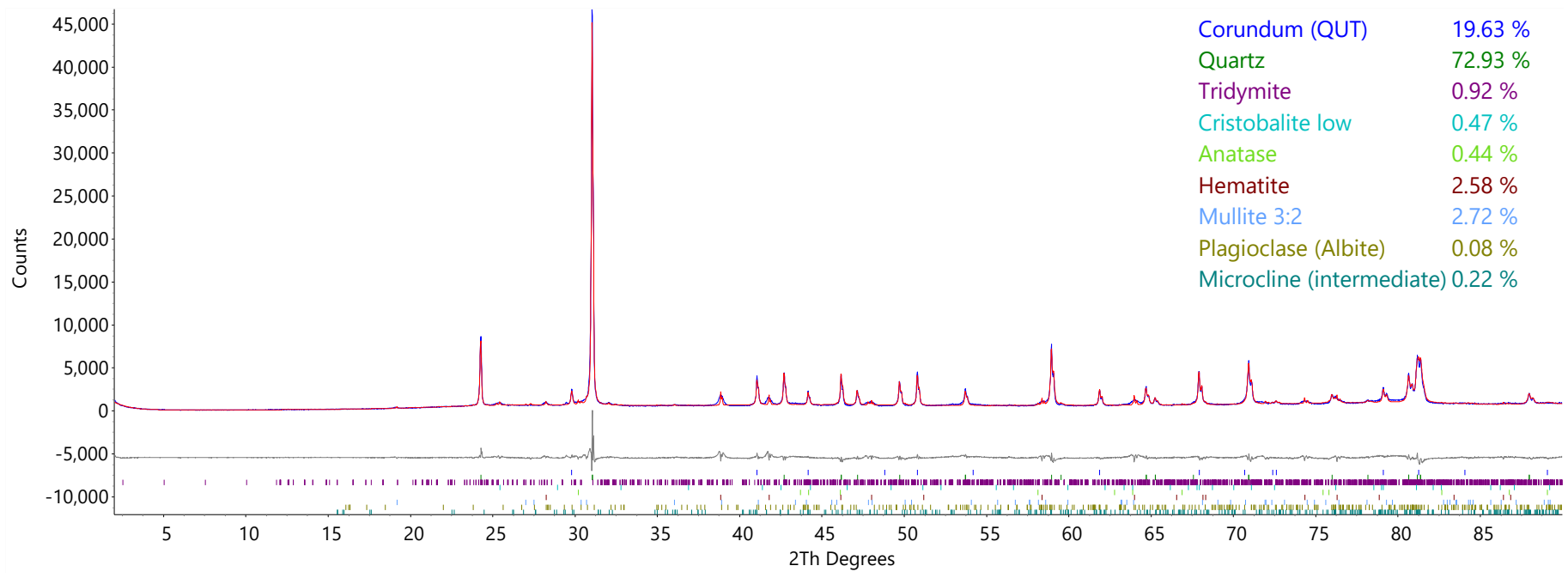

35/100/1200

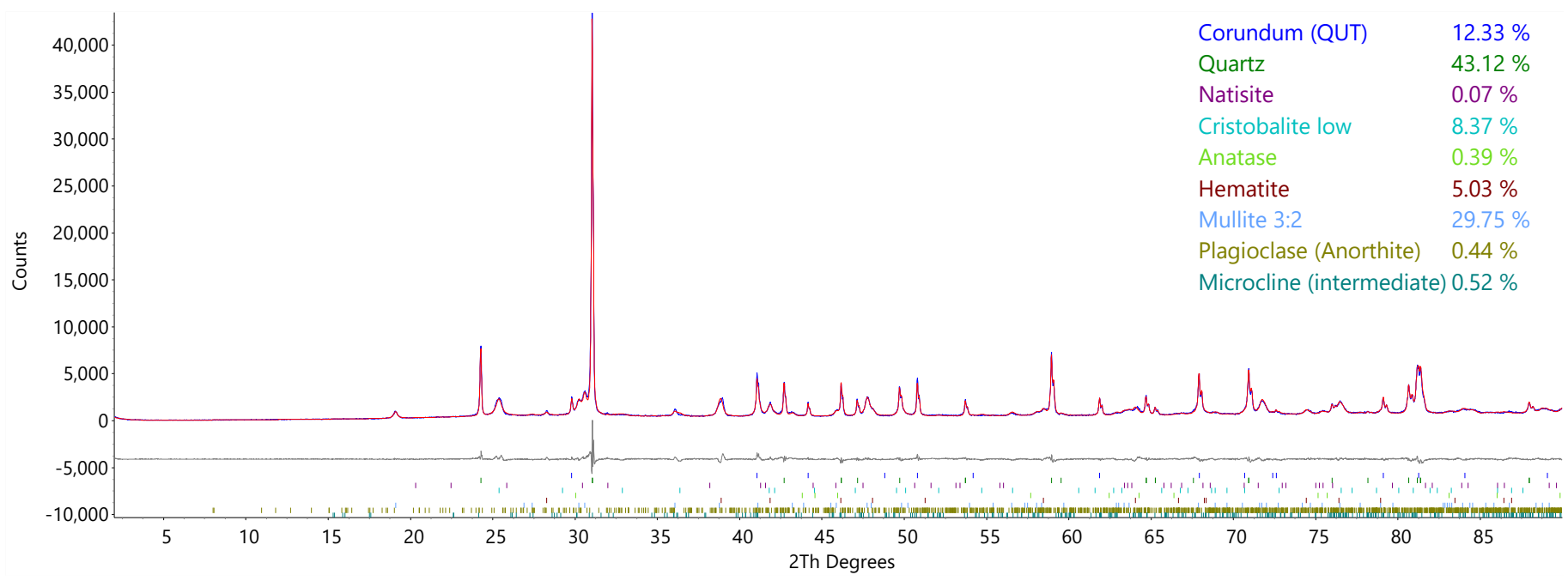

35/100/1350

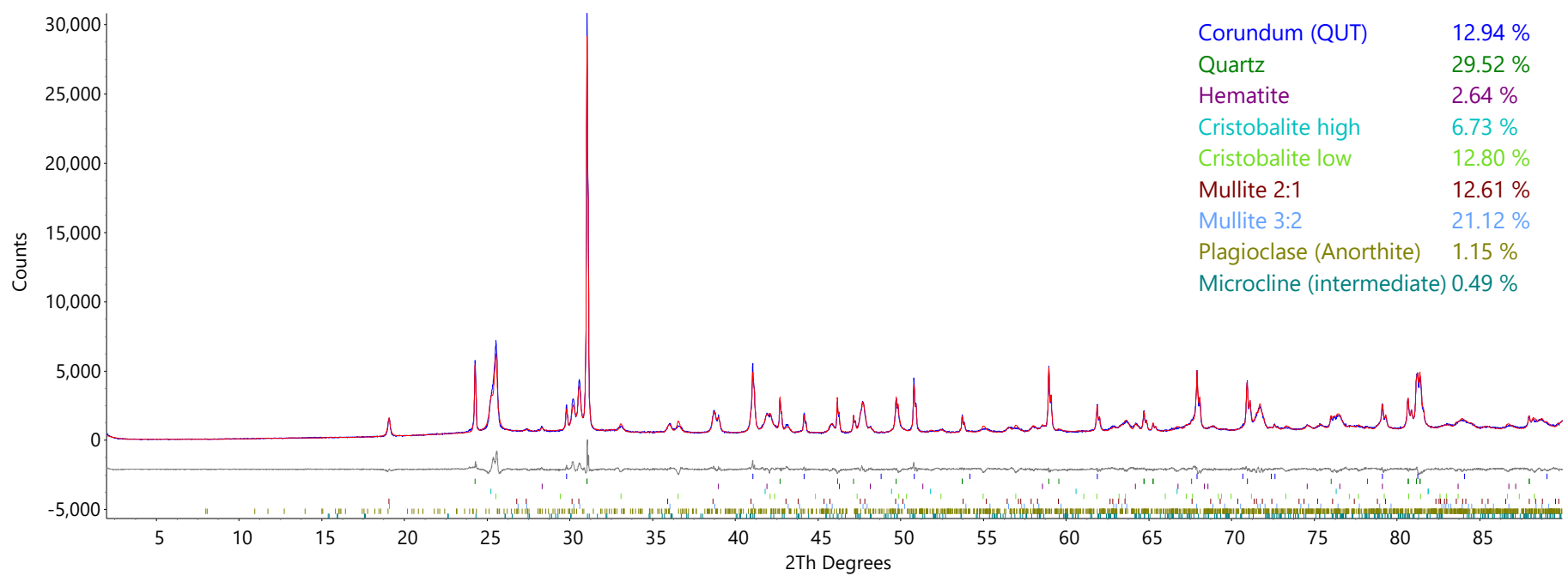

Supplementary Fig. 21 | Raw Rietveld refinement results of X-ray diffraction (XRD) patterns for the 35/100 formulation fired at 900°C, 1050°C, 1200°C, and 1350°C. The red line is the Rietveld refinement model; the blue line is the collected data, and the grey line is the difference. Tick marks correspond to different phases used in the refinement and expected peak positions for those phases. Approximately 10 wt.% corundum was added as an internal standard to enable quantitative determination of the amorphous content in the fired bricks.

50/0/900

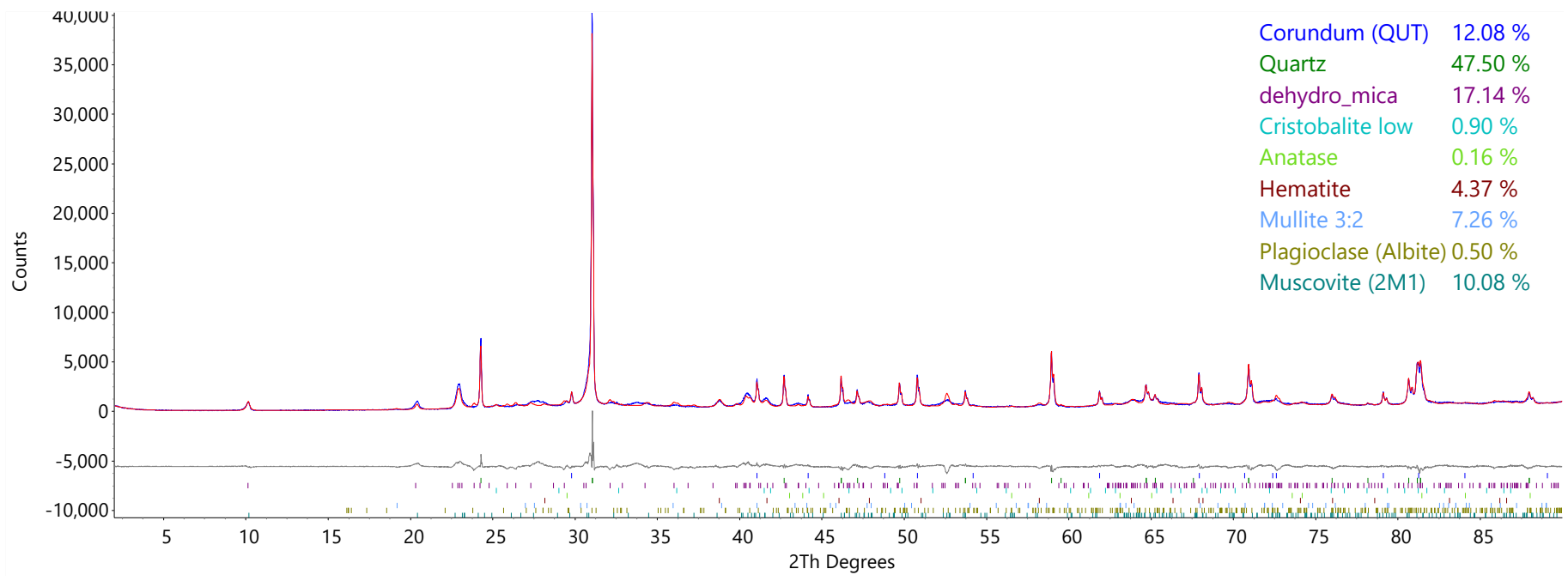

50/0/1050

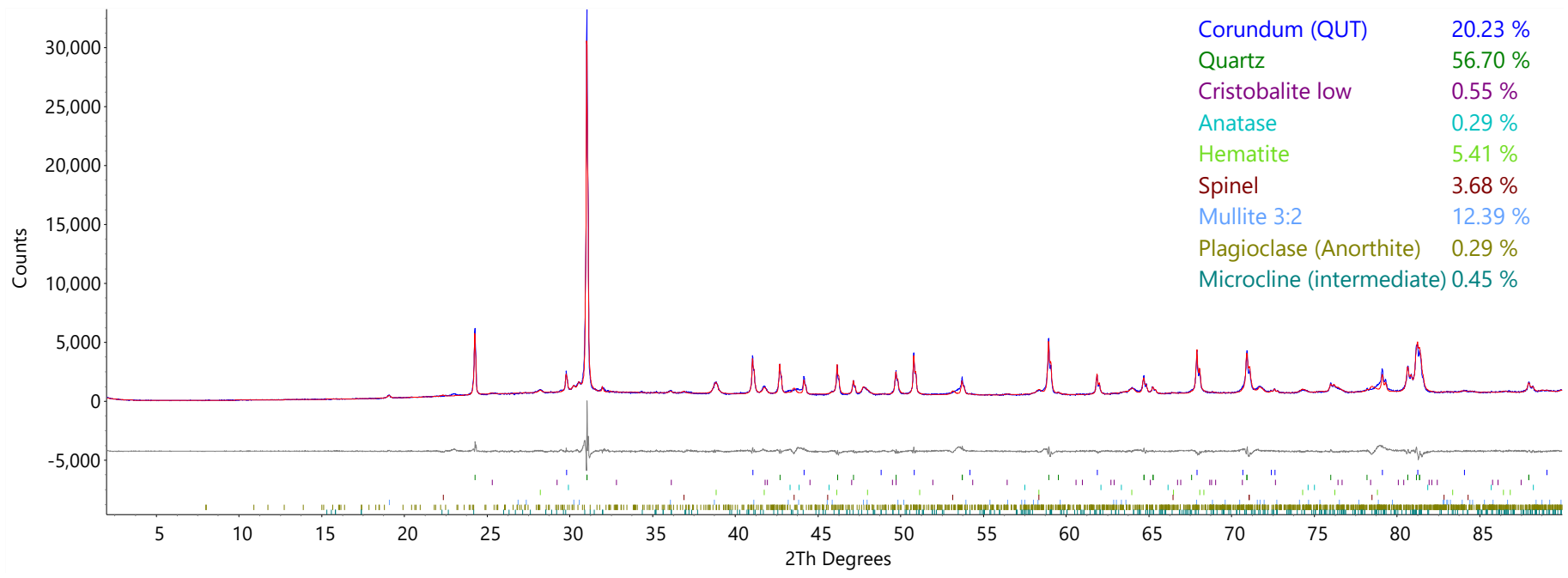

50/0/1200

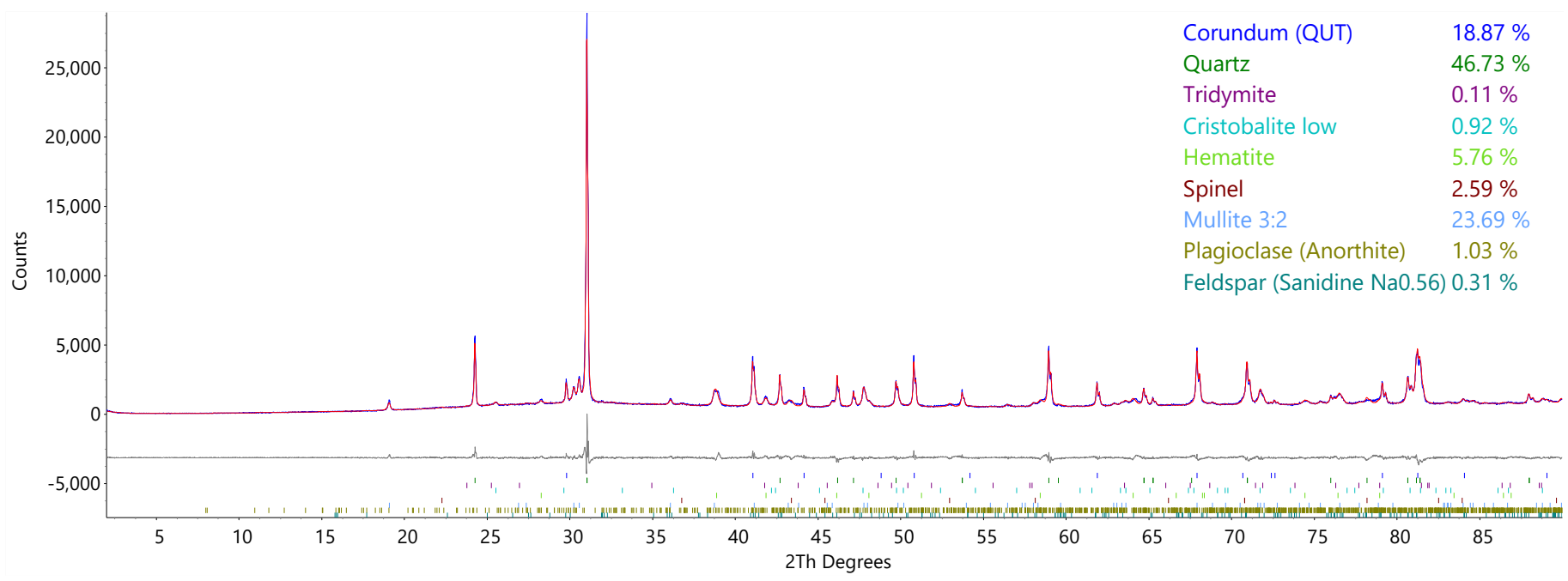

50/0/1350

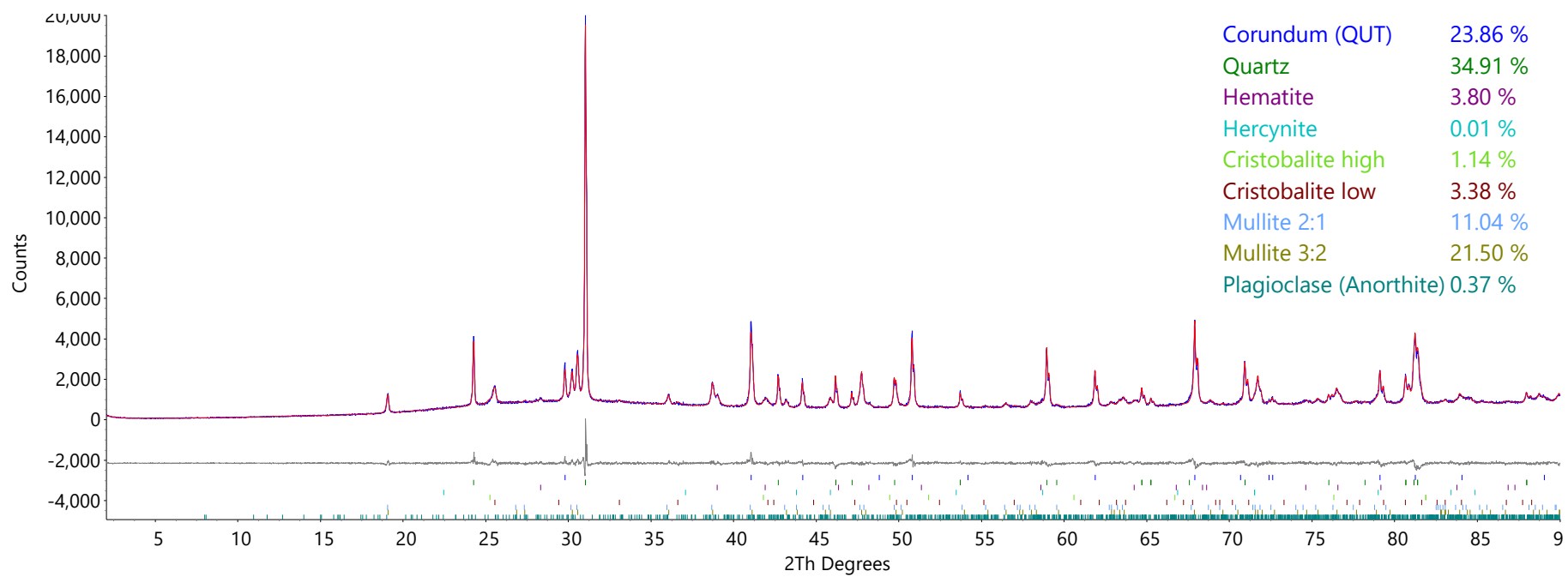

Supplementary Fig. 22 | Raw Rietveld refinement results of X-ray diffraction (XRD) patterns for the 50/0 formulation fired at 900°C, 1050°C, 1200°C, and 1350°C. The red line is the Rietveld refinement model; the blue line is the collected data, and the grey line is the difference. Tick marks correspond to different phases used in the refinement and expected peak positions for those phases. Approximately 10 wt.% corundum was added as an internal standard to enable quantitative determination of the amorphous content in the fired bricks.

50/50/900

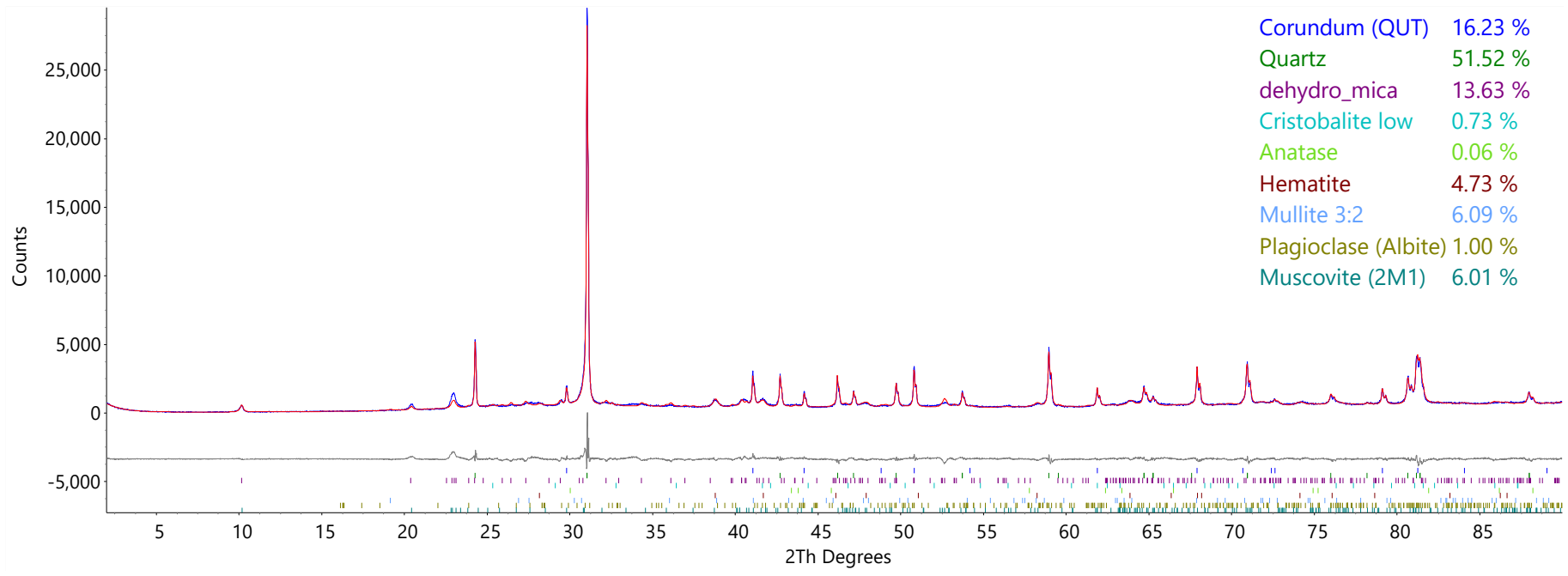

50/50/1050

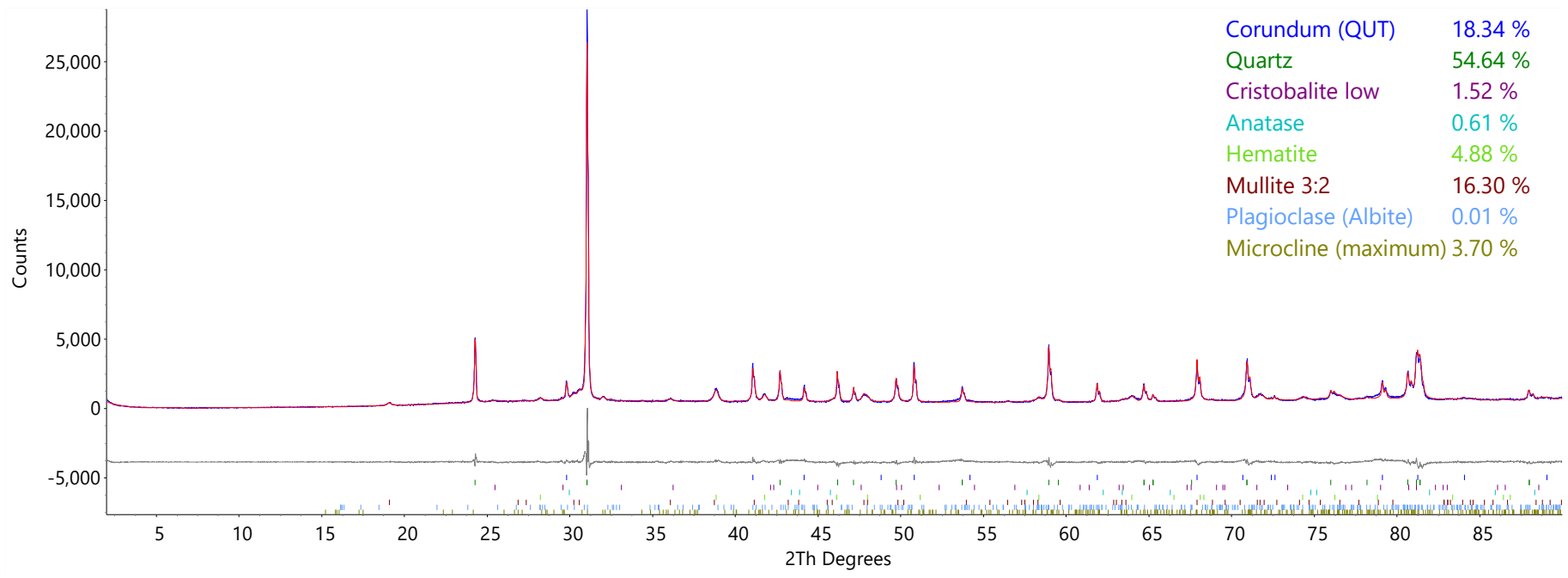

50/50/1200

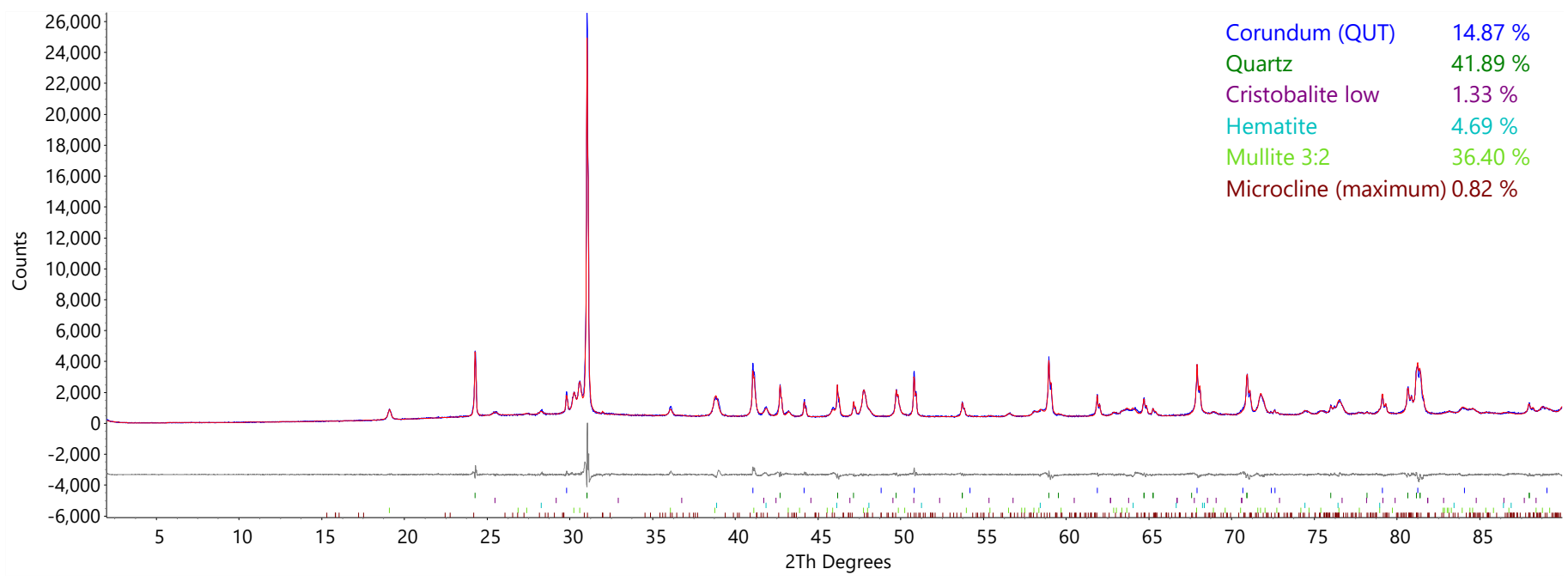

50/50/1350

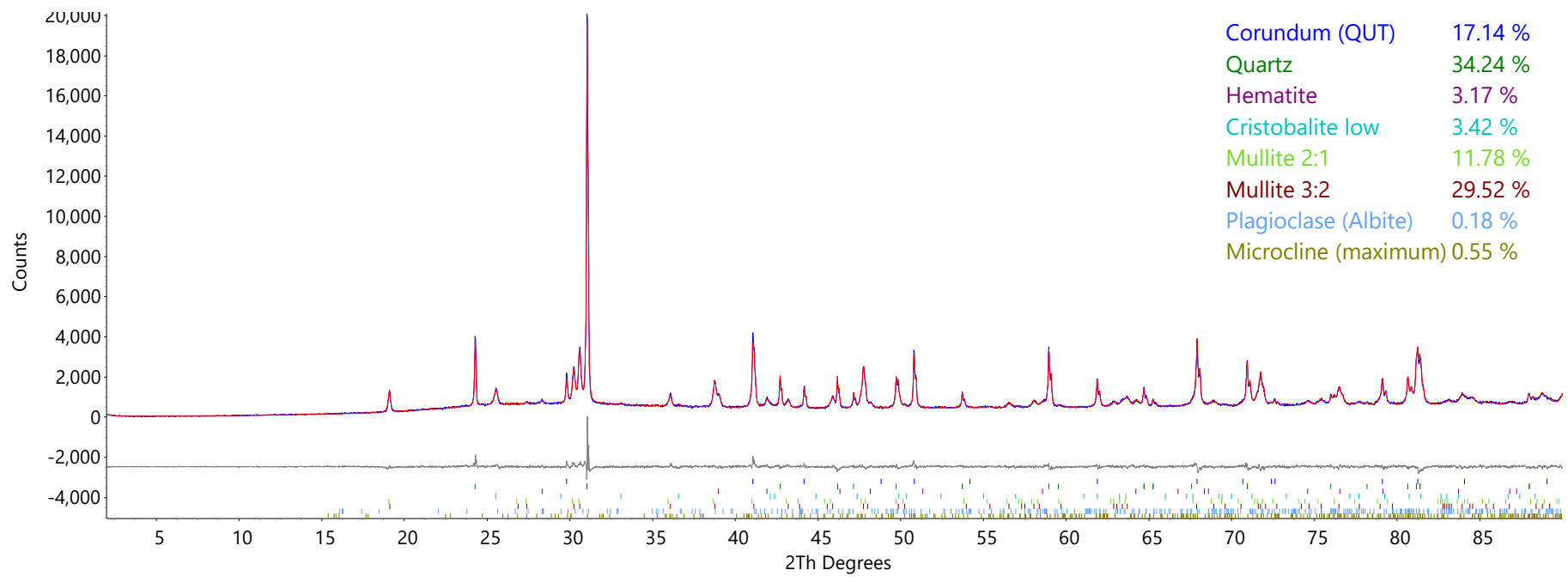

Supplementary Fig. 23 | Raw Rietveld refinement results of X-ray diffraction (XRD) patterns for the 50/50 formulation fired at 900°C, 1050°C, 1200°C, and 1350°C. The red line is the Rietveld refinement model; the blue line is the collected data, and the grey line is the difference. Tick marks correspond to different phases used in the refinement and expected peak positions for those phases. Approximately 10 wt.% corundum was added as an internal standard to enable quantitative determination of the amorphous content in the fired bricks.

50/100/900

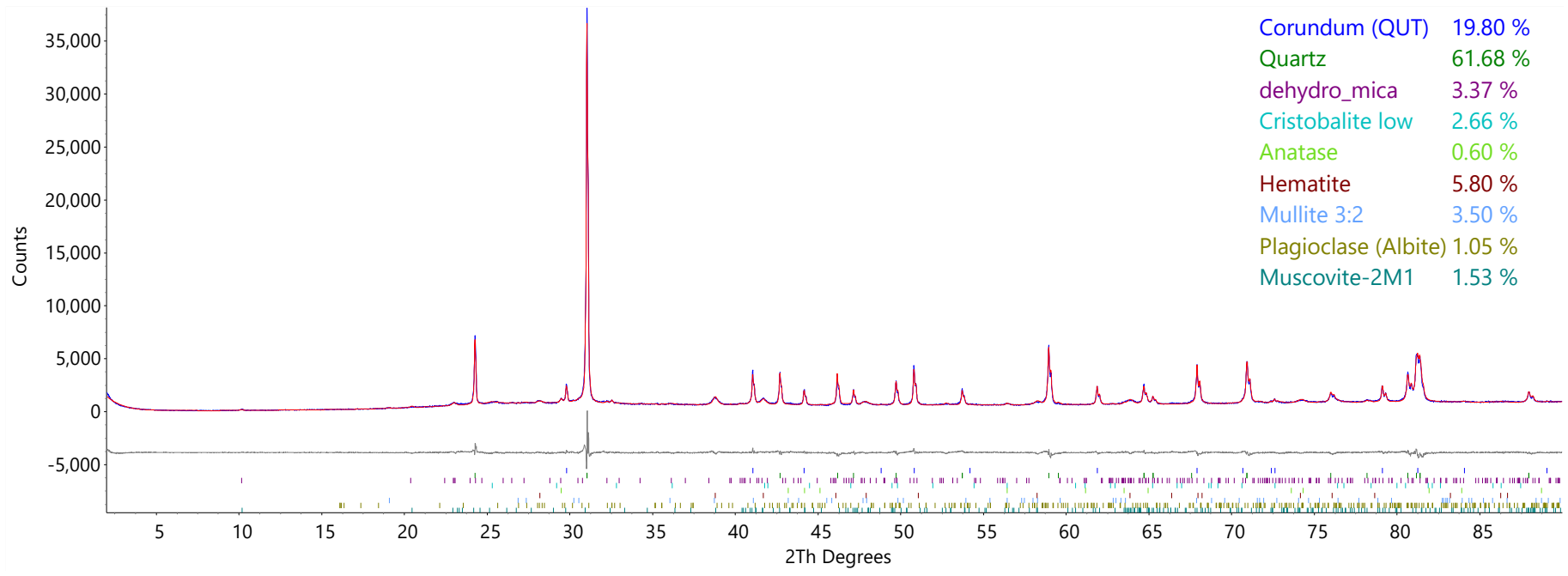

50/100/1050

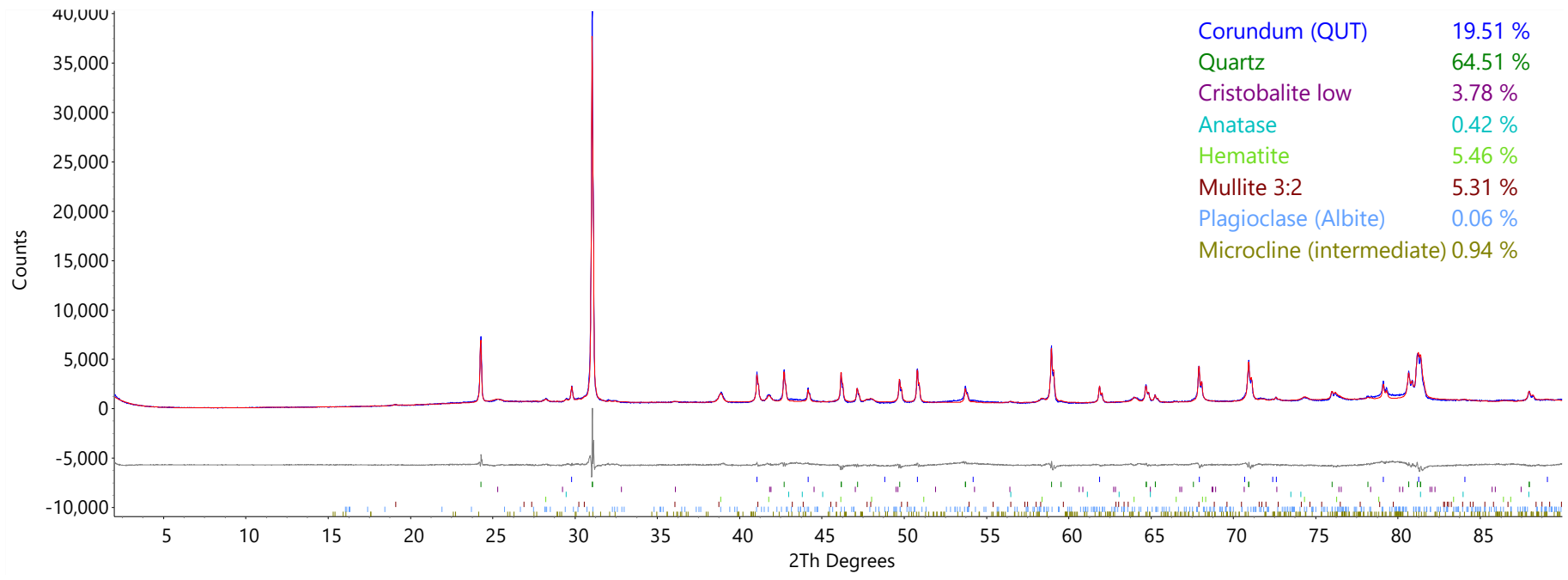

50/100/1200

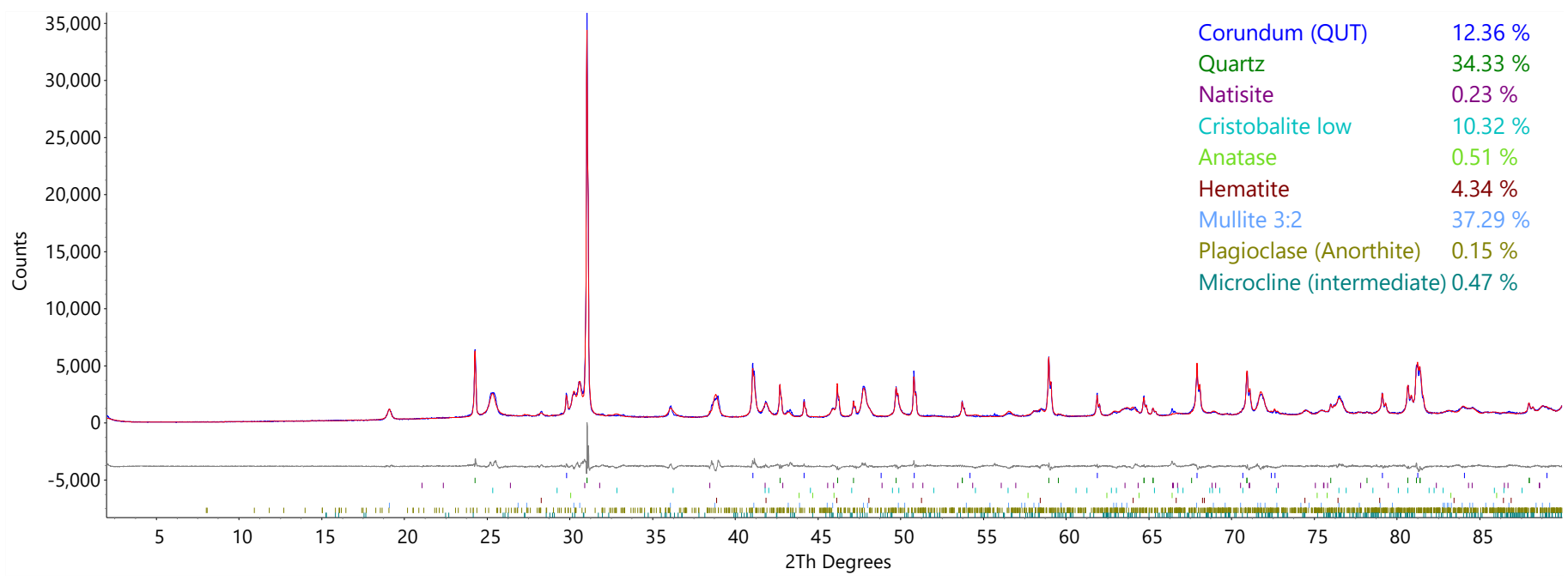

50/100/1350

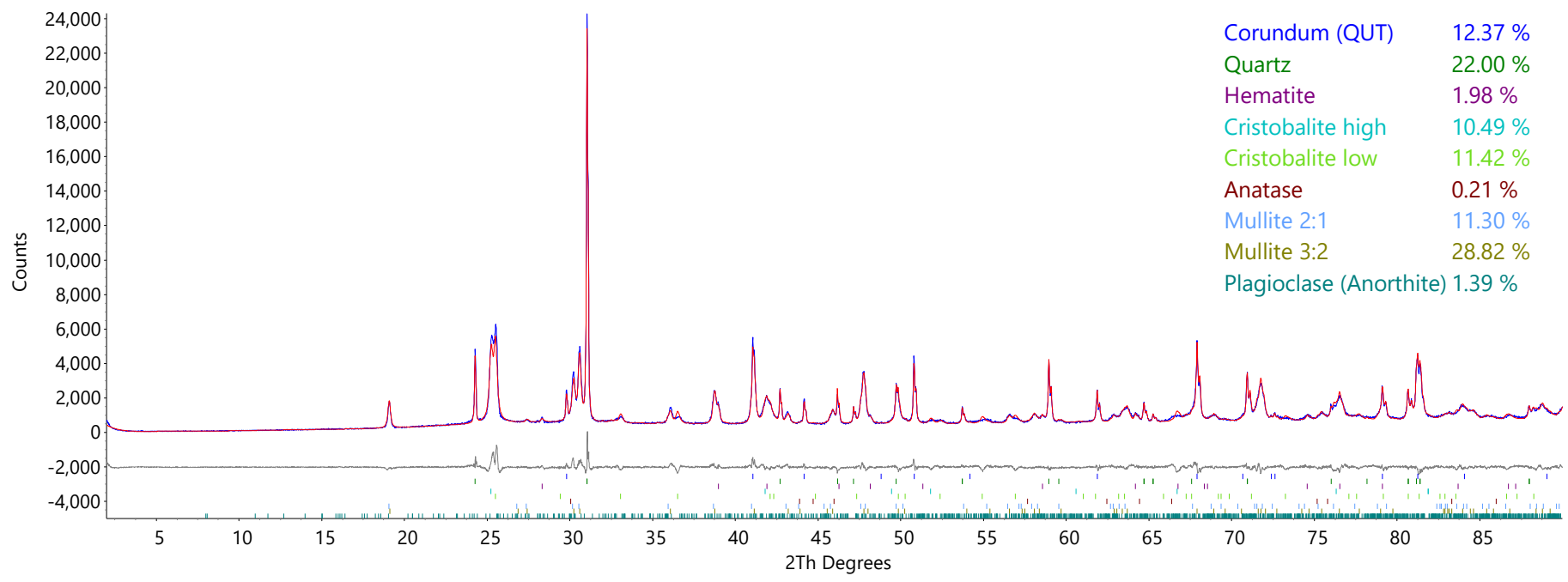

Supplementary Fig. 24 | Raw Rietveld refinement results of X-ray diffraction (XRD) patterns for the 50/100 formulation fired at 900°C, 1050°C, 1200°C, and 1350°C. The red line is the Rietveld refinement model; the blue line is the collected data, and the grey line is the difference. Tick marks correspond to different phases used in the refinement and expected peak positions for those phases. Approximately 10 wt.% corundum was added as an internal standard to enable quantitative determination of the amorphous content in the fired bricks.

20/33/1350

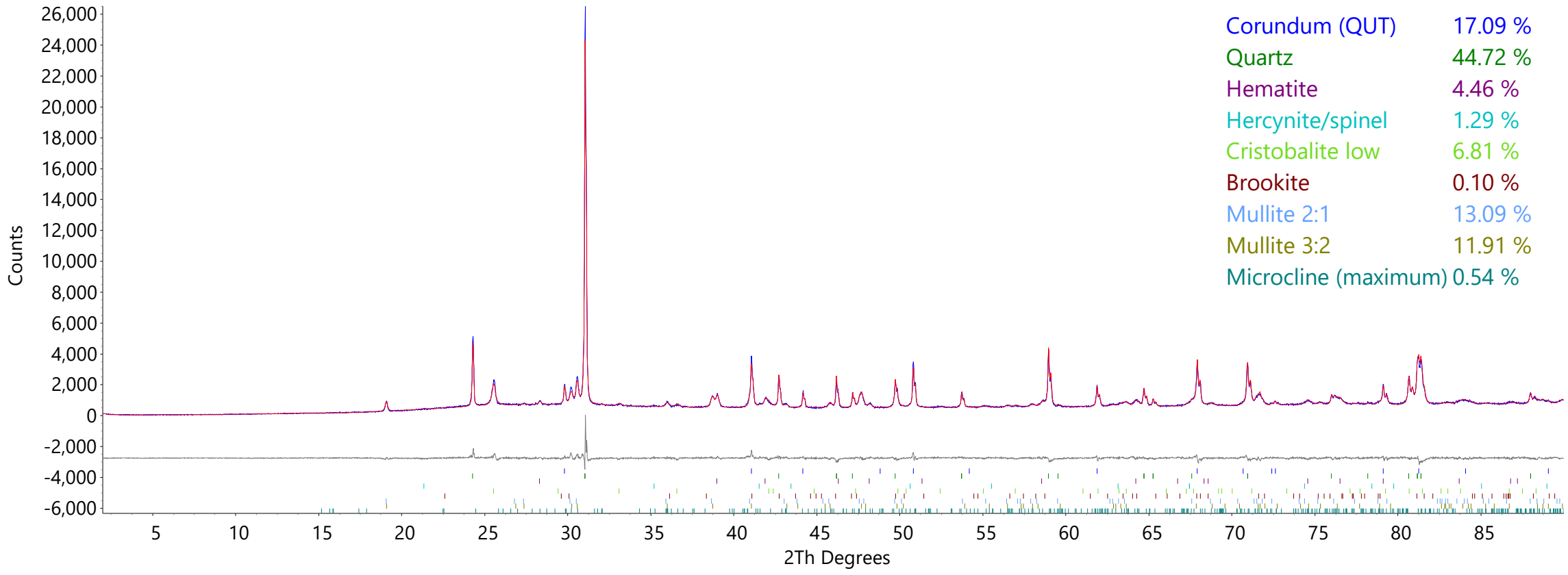

35/33/1350

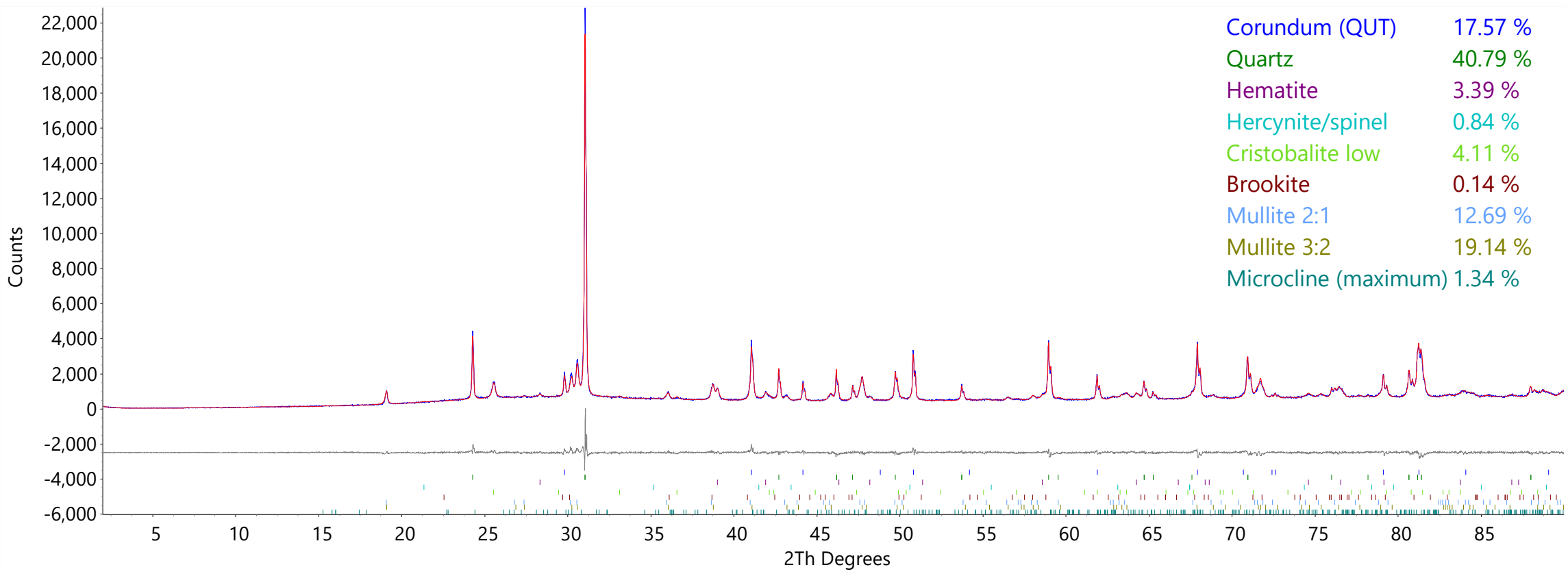

50/33/1350

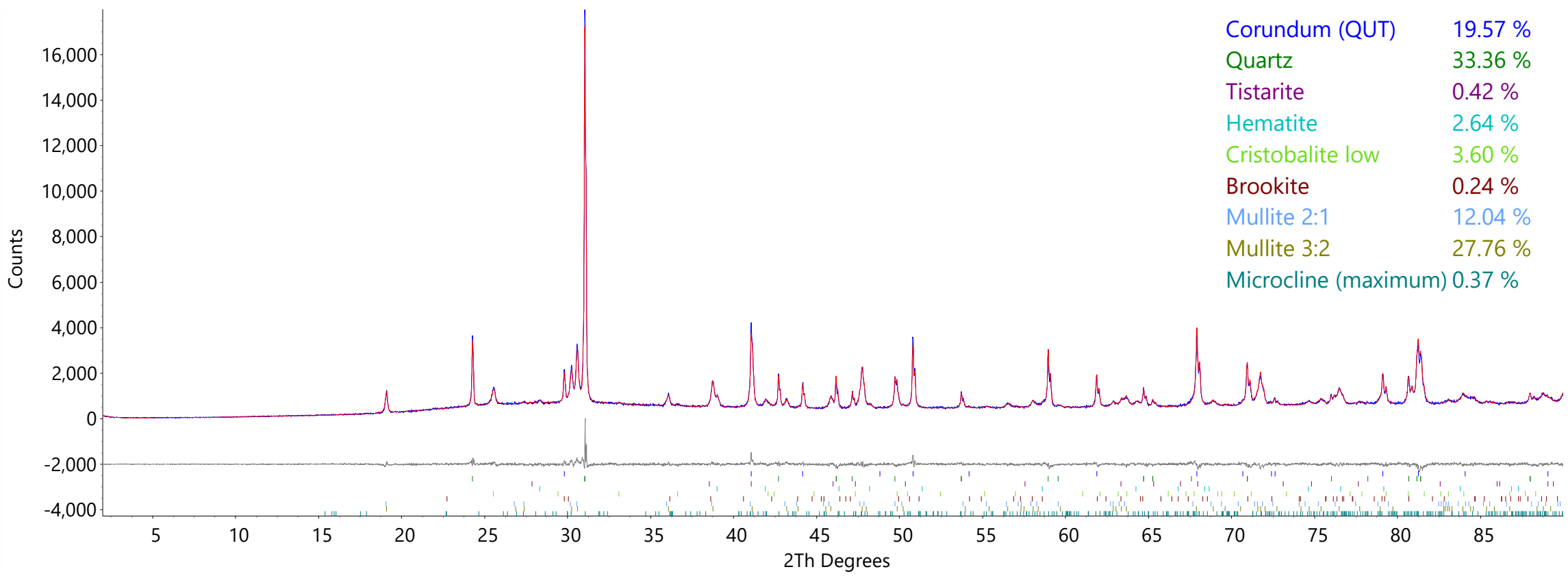

Supplementary Fig. 25 | Raw Rietveld refinement results of X-ray diffraction (XRD) patterns for the 20/33, 35/33, and 50/33 formulations fired at 1350°C. The red line is the Rietveld refinement model; the blue line is the collected data, and the grey line is the difference. Tick marks correspond to different phases used in the refinement and expected peak positions for those phases. Approximately 10 wt.% corundum was added as an internal standard to enable quantitative determination of the amorphous content in the fired bricks.

Supplementary Table 3. Comparison of compressive strength between brick buttons and full-size bricks.

Brick type	Firing temperature (°C)	Appearance	Compressive strength (MPa)	Conversion factor	Average conversion factor
Button	900	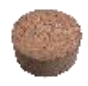	15.30	0.83	0.85
Full size		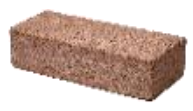	12.71		
Button	1050	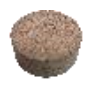	64.52	0.85	
Full size		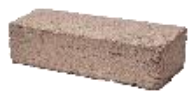	54.84		
Button	1200	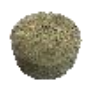	85.44	0.88	
Full size		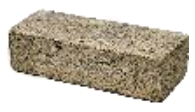	75.39		
Button	1350	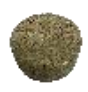	101.25	0.85	
Full size		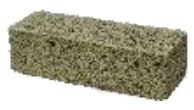	86.09		

Supplementary Table 4. Comparison of brick properties from the optimal formulation at different firing temperatures between this study and the literature.

Firing temperature (°C)	Clay origin	Brick dimension (mm)	Compressive strength (MPa)	Water absorption (%)	Bulk density (g cm^{-3})	Reference
900	KIQF	Φ 40 × 12	53.57	11.38	1.97	This study
900	Local brick clay, Thailand	140 × 65 × 40	20.18	~ 18	~ 1.7	[7]
900	Industrial brick clay, Turkey	20 × 60 × 100	31.7	24.3	1.76	[8]
900	Local brick clay, India	225 × 110 × 75	5.68	21.08	1.76	[9]
900	Local brick clay, Turkey	40 × 70 × 50	~ 16.9	~ 16.75	~ 1.85	[10]
1050	KIQF	Φ 40 × 12	126.21	7.14	2.11	This study
1050	Boral Bricks Pty Ltd, Australia	100 × 100 × 100, 300 × 100 × 50	25.65	5	2.12	[11]
1050	Cerámica Malpesa, S.A., Spain	130 × 30 × 18	n/a	7	2.01	[12]
1050	Industrial brick clay, Turkey	Φ 20 × 10	34.2	10.9	2.05	[13]
1050	Local clay quarry, Australia	100 × 100 × 100	~ 17	~ 18	n/a	[14]
900-1050	Industrial brick clay, Nepal	n/a	3.35-10.53	5-30	1.1-2.15	[15]
1200	KIQF	Φ 40 × 12	142.37	2.10	2.23	This study
1200	Cerámica de Alhabia S.L., Spain	60 × 30 × 11	113.75	9.98	~ 1.87	[16]
1200	n/a	60.25 × 15.20 × 8.15	55	22.26	1.58	[17]
1200	Local brick factory, Malaysia	67 × 33 × 20	90	6.75	n/a	[18]
1350	KIQF	Φ 40 × 12	172.43	1.84	1.95	This study
1350	n/a	Φ 20	75	n/a	1.95	[19]
1350	n/a	60 × 15 × 10	90	n/a	1.92	[20]
1350	Local clay, India	230 × 114 × 64	20-37	4-9	1.91-1.95	[21]

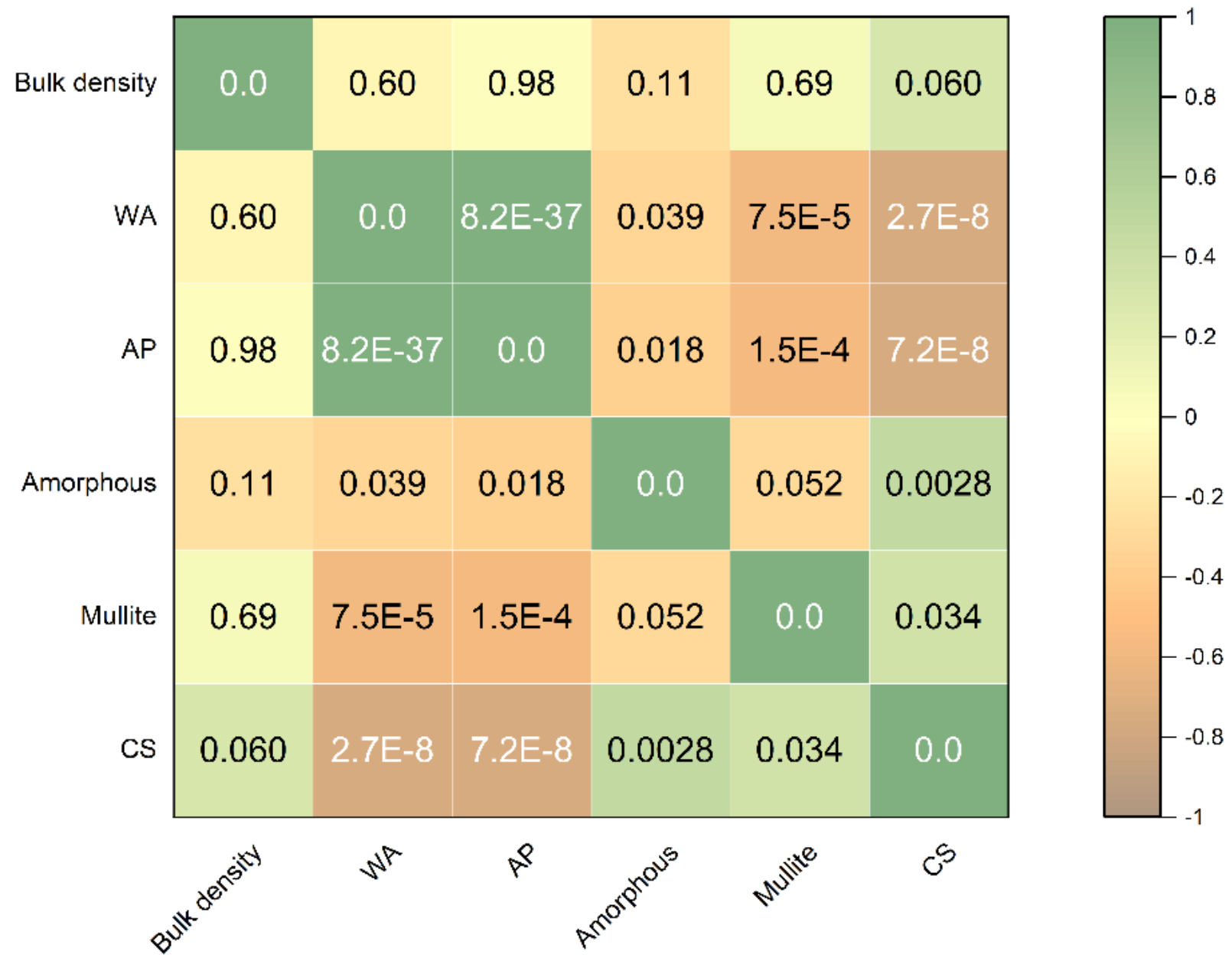

Supplementary Fig. 26 | *P*-values for correlation tests among brick properties across 900-1350°C. WA, water absorption; AP, apparent porosity; CS, compressive strength. Source data are provided as a Source Data file.

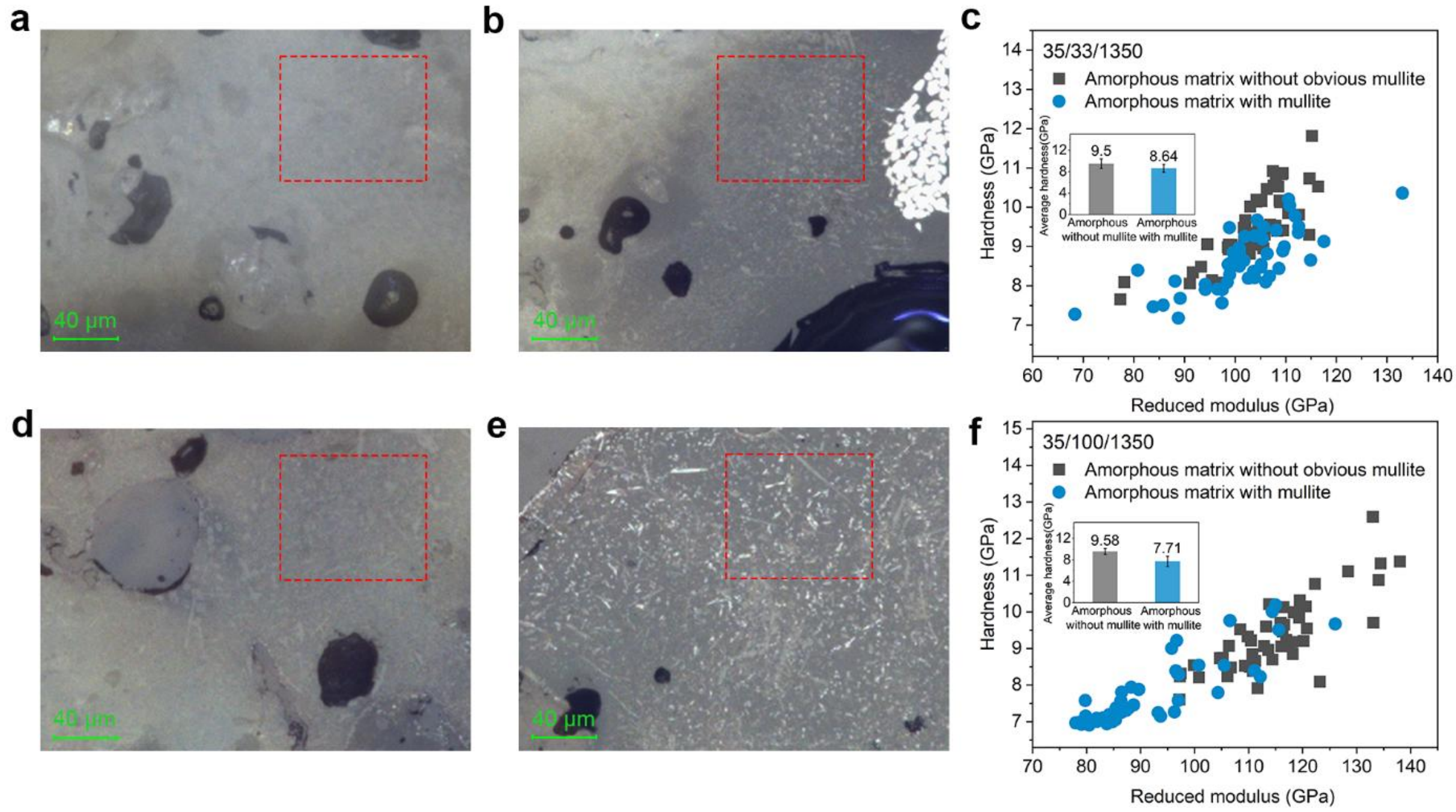

Supplementary Fig. 27 | Mechanical properties of amorphous matrix regions with and without embedded mullite crystals. a-c Amorphous matrix regions in the 35/33/1350 formulation **a** without discernible mullite crystals and **b** containing embedded mullite crystals, with **c** the corresponding reduced modulus and hardness measurements. **d-f** Amorphous matrix regions in the 35/100/1350 formulation **d** without discernible mullite crystals and **e** containing embedded mullite crystals, with **f** the corresponding reduced modulus and hardness measurements. Red dashed boxes indicate the tested regions. Insets in **c** and **f** show the mean hardness values obtained from 49 nanoindentation measurements ($n = 49$). Error bars represent the sample standard deviation (SD), calculated by dividing the sum of squared deviations from the mean by n - 1 and taking the square root. Source data are provided as a Source Data file.

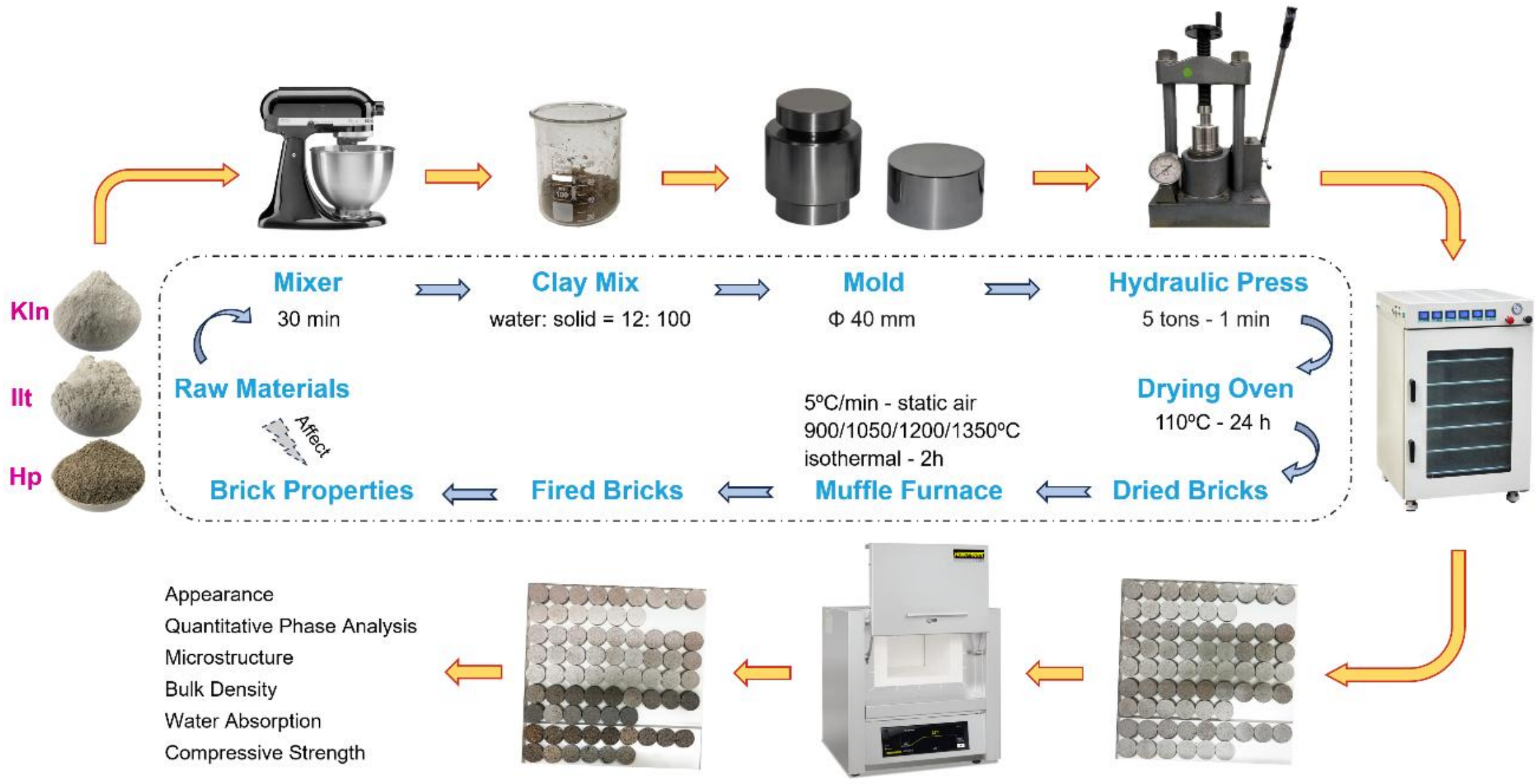

Supplementary Fig. 28 | Schematic illustration of the preparation of brick buttons.

Supplementary Table 5. *P*-values for correlation tests between compressive strength (CS) and other brick properties at each firing temperature.

Firing temperature (°C)	Bulk density-CS	Water absorption-CS	Apparent porosity-CS	Amorphous content-CS	Mullite content-CS
900	0.59	0.039	0.054	0.058	0.0014
1050	0.013	0.002	4.1×10^{-4}	0.23	0.22
1200	2.5×10^{-7}	3.4×10^{-5}	4.3×10^{-5}	0.0024	0.041
1350	0.018	0.027	0.15	0.032	0.047

Supplementary References

1. Lopes JS, Morais ML, Morais AIS, Bezerra RDS, Furtini JAO, Silva EC. A new method for reducing impurities in clay minerals. *Appl Clay Sci* **260**, (2024).

2. Castellini E, Berthold C, Malferrari D, Bemini F. Sodium hexametaphosphate interaction with 2:1 clay minerals illite and montmorillonite. *Appl Clay Sci* **83-84**, 162-170 (2013).

3. Junior ADN, Hotza D, Soler VC, Vilches ES. Analysis of the development of microscopic residual stresses on quartz particles in porcelain tile. *J Eur Ceram Soc* **28**, 2629-2637 (2008).

4. Cultrone G, Rodriguez-Navarro C, Sebastian E, Cazalla O, De La Torre MJ. Carbonate and silicate phase reactions during ceramic firing. *Eur J Mineral* **13**, 621-634 (2001).

5. Taylor NW, Lin CY. Effect of various catalysts on conversion of quartz to cristobalite and tridymite at high temperatures. *J Am Ceram Soc* **24**, 57-63 (1941).

6. Cabané A, Pelà L, Roca P. Anisotropy and compressive strength evaluation of solid fired clay bricks by testing small specimens. *Constr Build Mater* **344**, (2022).

7. Phonphuak N, Kanyakam S, Chindaprasirt P. Utilization of waste glass to enhance physical–mechanical properties of fired clay brick. *J Clean Prod* **112**, 3057-3062 (2016).

8. Gencel O. Characteristics of fired clay bricks with pumice additive. *Energy Build* **102**, 217-224 (2015).

9. Hossiney N, Das P, Mohan MK, George J. In-plant production of bricks containing waste foundry sand-A study with Belgaum foundry industry. *Case Stud Constr Mater* **9**, (2018).

10. Görhan G, Simsek O. Porous clay bricks manufactured with rice husks. *Constr Build Mater* **40**, 390-396 (2013).

11. Kadir AA, Mohajerani A, Roddick F, Buckeridge J. Density, strength, thermal conductivity and leachate characteristics of light-weight fired clay bricks incorporating cigarette butts. *WASET* **53**, 1035-1040 (2009).

12. de la Casa JA, Lorite M, Jiménez J, Castro E. Valorisation of wastewater from two-phase olive oil extraction in fired clay brick production. *J Hazard Mater* **169**,

271-278 (2009).

13. Sutcu M, Alptekin H, Erdogmus E, Er Y, Gencel O. Characteristics of fired clay bricks with waste marble powder addition as building materials. *Constr Build Mater* **82**, 1-8 (2015).

14. Bilgil A, Simsek O, Sevim Ö, Demir I. Development of lightweight and thermally efficient clay bricks using expansion additives: effects of firing temperature and additive ratios on physicomechanical properties. *J Aust Ceram Soc* **61**, 1351-1363 (2025).

15. Chapagain YP, Sapkota S, Ghale DB, Bohara NB, Duwal N, Bhattarai J. A case study on mineralogy and physico-mechanical properties of commercial bricks produced in Nepal. *SN Appl Sci* **2**, (2020).

16. Martínez-Martínez S, Pérez-Villarejo L, Garzón E, Sánchez-Soto PJ. Influence of firing temperature on the ceramic properties of illite-chlorite-calcitic clays. *Ceram Int* **49**, 24541-24557 (2023).

17. Yu L, Zhang Y, Wang H, Zhang Z, Shen X, Liu H. Enhancing the sustainable production of cost-effective ceramics with high strength using kaolin, sewage sludge and blast-furnace slag. *Chem Eng J* **480**, 148184 (2024).

18. Johari I, Said S, Hisham B, Bakar A, Ahmad ZA. Effect of the Change of Firing Temperature on Microstructure and Physical Properties of Clay Bricks from Beruas (Malaysia). *Sci Sinter* **42**, 245-254 (2010).

19. Yu L, Zhang Y, Zhang Z, Mao H, Han H, Yang J. Recycling reuse of municipal sewage sludge in sustainable structural materials: Preparation, properties, crystallization and microstructure analyses. *Constr Build Mater* **398**, 132507 (2023).

20. Zhang Y, Yu L, Wang J, Mao H, Cui K. Microstructure and mechanical properties of high strength porous ceramics with high sewage sludge content. *J Clean Prod* **380**, 135084 (2022).

21. Sahani AK, Ghosh S, Samanta AK, Adhikari R. Influence of fly ash on refractory properties and microstructural characteristics of sustainable refractory bricks. *J Inst Eng (India): Ser A* **105**, 317-329 (2024).
