# Supplementary material for: From ancient practice to modern innovation solving the clay mineral puzzle in brickmaking
Source: Nat Commun. 2026 Oct 14;17:11596. doi: 10.1038/s41467-026-78652-0 (PMC13752296; doi:10.1038/s41467-026-78652-0)
Supplement: Supplementary file 2 [file 41467_2026_78652_MOESM2_ESM.pdf]

nature portfolio

Peer Review File

From Ancient Practice to Modern Innovation Solving the Clay Mineral Puzzle in Brickmaking

Corresponding Author: Professor Yunfei Xi

This file contains all editorial decision letters in order by version, followed by all author rebuttals in order by version.

Version 0:

Reviewer comments:

Reviewer #1

(Remarks to the Author)
The paper introduces a tunable Kaolinite–Illite–Quartz–Feldspar (KIQF) system that isolates the roles of kaolinite (Kln) and illite (Ilt) in brickmaking. Overall, the topic is important for ceramics and industrial brick manufacture. The systematic grid (60 mixes) and mineralogical analysis (in-situ HT-XRD, Rietveld with internal standard, SEM/EDS) are strong and, if tightened methodologically, the work would be impactful.

Please find my comments as follows.

1. "10× ASTM" strength framing. The comparison uses button specimens (Ø40 × 12 mm) in compression rather than standard brick units, so direct benchmarking to ASTM C62 unit strengths risks a geometry/size-effect bias. Please justify this equivalence or rephrase the claim conservatively; also report variability.

2. CS–mullite negative correlation. The statistical signal is interesting, but p-values/CI and sample counts per regression are not reported. Please provide n and statistics for each correlation and clarify whether fits pool all temperatures or are stratified (Figure 7 mentions both views).

3. Illite "melts" at 1050 °C. Main text states pure Ilt "melts" at 1050 °C, while SI maps indicate Ilt is nearly fully melted at 1200 °C with Kln still largely unmelt. Consider softening to "intense vitrification by ~1050–1200 °C" for consistency across sections.

4. Over-firing/closed-pore mechanism. The explanation is plausible and the SEM evidence compelling; adding quantitative porosity (open vs closed) via MIP or micro-CT on key points (e.g., 50/0 at 1350 °C) would make the mechanism and the ~10% expansion claim rock-solid.

5. Replicates and error bars. The manuscript shows 72 dried buttons in SI (suggesting 60+ specimens), but the number tested per condition for CS/WA and the spread (SD/SE) are not reported. Please specify replicates and plot error bars/CI for CS, WA, density, and phase fractions.

6. Firing schedule. Figure S25 is schematic; please provide exact ramps, soaks, cooling used for firing the buttons (time–temperature history), as this strongly affects phase assemblage/porosity.

7. Pressing details. State compaction pressure, dwell time and release protocol for the hydraulic pressing step (now "hydraulic compression" only).

8. Statistics for correlations. Report n, p-values, and confidence intervals for Pearson r/R^2 (Figure 7), ideally with temperature-stratified analyses and a multiple-testing note.

9. The temperature-dependent role reversal (Kln helpful at 900 °C; Ilt at 1050–1200 °C; mixed at 1350 °C) is well argued and consistent with flux content (K-rich Ilt) and observed vitrification differences. Consider adding a compact decision chart mapping firing window → optimal Kln/Ilt (you already outline this in §2.4, including Fe_2O_3 threshold guidance ≥ 6.5%).

10. Quartz/cristobalite effects at 1200 °C linked to CS dips are reasonable; a brief note on thermal cycling implications (β↔α cristobalite) would help contextualise durability claims.

11. The conclusion that glass fraction (amorphous) matters more than mullite for CS in coarse brick bodies is persuasive; still, a small nanoindentation/phase-specific modulus dataset or fractography on selected mixes would substantiate the micro-mechanics behind Figure 7.

12. Use consistent terminology for Ilt behaviour ("vitrifies/intensely vitrifies" rather than "melts") across main text and SI.

13. Consider a table comparing your best mixes with typical industrial ranges (CS, WA, density) to emphasise practical relevance (references already cited in Intro/Discussion).

Reviewer #2

(Remarks to the Author)
This manuscript is of high quality and presents an innovative contribution. The authors have addressed a relevant topic within brickmaking sector, demonstrating how certain physical properties of bricks vary according to the kaolinite or illite content of the starting material and the firing temperature.

The manuscript is clearly structured and well organized. The data and analysis are comprehensive and appropriately documented. I believe the study is of interest for an international audience. Nevertheless, in my opinion, the manuscript requires some revisions before it can be considered for acceptance.

My main concerns are as follows:
1) The criteria used by the authors to define the independent variables are not entirely clear to me. For instance, in the case of total clay mineral content, why is Hp included in the ratio of clay minerals, if the clay minerals were already separated? Moreover, why is the kln-to-clay ratio considered but no the Ilt-to-clay ratio? I think a more detailed explanation could be given.
2) A porosimetric analysis of the samples is lacking. Throughout the manuscript, the authors discuss the influence of kaolinite and illite on porosity. Including this analysis would substantially strengthen the study by testing whether the higher proportion of kaolinite or illite affects the total porosity of the bricks.
3) Although a large number of brick formulations were studied, it is unclear whether all of them were analyzed using the same techniques. For example, the thermal dilatometry data (Figure 1) are reported only for mixtures with a total clay content calculated at 35%, whereas the supplementary materials (Figure S6) include mixtures with a kaolinite-to-clay minerals ratio at 50%. Likewise, for electron microscopy, images are provided only for formulations with a kaolinite-to-clay minerals ratio at 35%.

Some other minor comments are indicated below:
-Line 68: "given the annual production of over 1,500 billion bricks". Is this data referring to worldwide production, and to which year does it correspond?
-Line 126: Please indicate "kaolinite-illite-quartz-feldspar" here, as this is the first occurrence of the abbreviation KIQF.
-Mullite Formation section (lines 295-323). Some additional references could be incorporated here. There are numerous studies describing mullite formation from metakaolinite in different ceramics.
-Figures S1, S2 and S3: Are Hp, Kln and Ilt samples here raw or purified? Including a comparison of the XRD results before and after purification would significantly improve the clarity of this section.

Version 1:

Reviewer comments:

Reviewer #2

(Remarks to the Author)
The authors have properly addressed all comments and have included new data into both the manuscript and the supplementary material, thereby strengthening the study. I consider that the manuscript is now suitable for publication.

Reviewer #3

(Remarks to the Author)
Review of Nature Communications manuscript # 616272 "From ancient practice to modern innovation: Solving the clay mineral puzzle in brickmaking.

I have read the Responses to the Reviewers, the revised manuscript and supporting information. I had not seen the original manuscript nor been involved in any prior review process. The authors responded thoroughly to the reviewer's comments and critiques and adequately incorporated their concerns into the revision
The authors comprehensively utilized reductive analytical processes to disassemble a brick making clayey substrate into three main components and then re-assembled these three components into new brick making material with a wide range of compositions- ranging from low total clay mineral to high total clay mineral and from no kaolinite to no illite (where kaolinite

and illite were the only clay minerals identified and present). This alone represents an enormous amount of work – but the authors then applied a variety of well-considered analytical methods ranging from thermal dilatancy, compressive strength, amount of open and closed porosity to in situ high temperature XRD to assess quantitatively the impact of varying kaolinite concentration (from 0 to 100%) in the clay mineral fraction as well as the total clay mineral content (20, 35 and 50%) in the material when fired at selected temperatures. A higher Illite content of clay-rich (50% total clay mineral content) brick making materials induced better strength at intermediate (and more commonly used) temperatures of 1050 and 1200 C. The highest temperature (1300 C) achieved the highest strength only for low (35%) clay mineral content in which illite:kaolinite ratio was 2:1.
The manuscript is generally very well-written, rather comprehensive in coverage of the expected thermally induced responses such as the dependence of best firing temperature (chosen for efficiency vs strength) on optimal clay mineral assemblage, but also as a result of the approach used, provides insight into why "unexpectedly observed" increased mullite content was inversely related to compressive strength (most obvious at relatively low 900C) due to excess conversion of vitrified material.

I recommend publication after minor further revision for the following reasons and per details provided below.
Regarding the compressive strength results there seem to be some misinterpretations. For example L441-443 – the authors state that at intermediate temperatures (1050 and 1200C) vitrification densified the bricks so that higher clay mineral content increases CS, but this is only true at low Kln contents (< 35% Kln for the most part).
In the section on correlations of various parameters with compressive strength – there needs to be more consistent use of statistical language for example defining a strong correlation as having an r^2>|0.75|, moderately strong (|0.5|<r^2<|0.75|) and an r^2<|0.5| as weak would seem appropriate for the complexity of the inter related interactions between the various known parameters. Obviously the authors can choose their own boundaries.
Further, the authors need to make clear where they use temperature-combined data vs temperature separated data. The former tend to mask correlations whereas the latter tend to highlight them. For example the statement on L554-555 regarding the correlation between bulk density and CS is true only for temperature-combined data. For the temperature separated data, the relationship is strong at 1200 C , moderately strong at 900 and 1350 C and only weak at 1050 C. The authors do point out that there may be confounding interactions that influence the "scatter" in the data, such as sintering-induced mullite formation, over- or under-vitrification of illite etc., which undoubtedly are at play – but another experimental error that could be considered may be – inadvertent differences in the fines content of the Hp between various mixes. Such considerations may also explain some of the lack of correlations.
In the Discussion – which is otherwise actually quite informative regarding the nuances of the dataset – some further aspects of the (non) universality of the results should be discussed in light of the given brick making material studied herein – in which the authors comprehensively disassembled it into three main components and then re-assembled these three components into a wide range of compositions- ranging from low total clay mineral to high total clay mineral and from no kaolinite to no illite (where kaolinite and illite were the only clay minerals identified and present) – given the known impacts of chemistry, crystallinity and particle size of clay minerals on their reactivity, thermal stability and etc. For example, would on expect a deposit chosen for brick making in e.g. Chechia to behave more or less alike with respect to mullite formation and/or dilatancy / vitrification / pore formation? For example the both the kaolinite and illite in this material had relatively low iron contents, so could the authors comment on how a less weathered material – with greater iron content within the structure of the layered clay minerals – behave with respect to mullite formation?

Details in Manuscript
(many syntax (non-exhaustive); some factual issues; some comments for clarification)

L40-41 two of the most critical clay minerals for brick making –
L42 strengths
L43 methods
L44 uncovered a
L46 resonses
L47 phases; and over-

L59 oxyhydroxides(^2,3) have made … understand the underlying
L60-61 Unclear rationale. Suggest adding to end … production of homogeneously performing product.
L68 high – at approximately
L69 delete etc.
L70-72. Resource use and availability of suitable virgin resources are also big issues with materials for construction worldwide.
L74-75 "defects" may be more due to weathering processes resulting in different defects from different parent material (primary factor. For e.g. granitic parent materials the inherent crystallinity and chemical composition of the main mineral constituents will influence how a given weathering regime will affect the clay minerals formed.
L75 practice because most (combine sentences)
L80 echos
L105 roles of the clay minerals Kln and Ilt in

L121 Precisely tuning clay minera's./// Phrase here is unclear. If the authors mean defining the composition of Kln and Ilt via purification methods then simply state that. Don't use jargon.
L123 the common ecoexistence
L124 making their separation
L126 purity being rare

L134-136 contraditctory? Just mentioned above (L125-126)they are rarely high pure. Possibly the authors mean here that they applied procedures to isolate Kln and Ilt as implied from the next sentence.
L139 potential issues with values see comment on XRD (Figs S16-S24) and use of corundum.
L143 sizee distributions
L144 replace performed with measured
L146-149 ? Fig S5 doesn't show this at all. S5a shows a two overlapping (normal) distributions for Kln centered near 1 micron and minor (<~5%) larger particles than 10 um and virtually all < 30 um (cumulative %). For Ilt S5b shows a normal distribution centered near 4.5 um. Also most of the HP (~75%) has particles < 50 um with most of those averaged near 5 um.
L162 Suggest making differentiating the clay minerals from the heavy particles in this manner particularly
L164 Suggest optimization practices typically used in industrial

L181 Fig 1a Authors should indicate the processes occurring in stages 1 and 2 in Figure 1a. Choice of colors not ideal in as it is hard to differentiate the light greens and light blues, particularly with the dotted lines.
L188-190 seems the authors are putting the cart before the horse here. Also seems it would be clearer to state shrinkage rather than expansion decrease?
Suggest In stage 3 brick expansion abruptly accelerates rapidly due to conversion of alpha- to beta quartz near 573C (Fig 1a). This behavior is partially counteracted by dehydroxylation of Kln resulting in its structural collapse and increases with Kln/(Kln+Ilt) ratio.
Also...... Kln dehydroxylation temperature is dependent on many aspects of kaolin chemistry and crystallinity, so it seems serendipitous for Kln dehydroxylation to correspond quartz converstion.
L191 Greater incorporation of Kln can reduce cracking
L192 effect at this temperature because
L192-193 Has the Ilt in this particular brick making material (or surrogate) dehydroxylated by 570C? Highly unlikely. Have the authors conducted TGA to show the dehydroxylation temperatures of the purified Kln and Ilt?
Like Kln, ILT dehydroxylation temperatures are dependent on chemistry and crystallinity, but additionally on the occupation of the octahedral sheet. Authors need to acknowledge this fact in their interpretations.
L198 matakaolin prior to mullite formation. Comment mullite is a spinel.
L207 ? viscous-phase formation ?

L288-229 At most this would be about 3% - is that sufficient when Ilt fully dispersed within Hp? Could this effect be more related to the PSD of the Hp?
L238 comment on item 3). Seems rather a loss of smaller pores. Data in Fig S8, could show this more effectively, depending on the voxel size - but unclear if a Kln100/Ilt0 image measured?
L260 vitrification at 900C.
L282 hercynite, being a spinnel, would this possibly be the cause of the expansion of the 900C Ilt100 sample observed? Al cations migrate within the Ilt octahedral sheet? (a well-established reaction, see works of Drits or MacCarthy and Drits).
L299 should indicate that the total clay mineral content in Fig 3a,d and g is 35%.
L322 ?? mullite is an Al spinel! it is the stable intermediate in the Al-Si system
L361 phases
L370 strongly interdependent with temperature
Fig 3b,c,d, f,g,h,J,k,l. There exist crossovers in WA : as a function of total clay content and ratio of Kln (b); as a function of Ilt content and total clay mineral content (c); as a function of clay mineral content at high Kln content (d) (data for and d are complimentary). All are interrelated to apparent porosity (f, g, h) as well as bulk density (j,k,l).
L439-440 Also greater Kln content
L441-443 Ture only for Ilt rich mixes
L445-446 due to lower vitrification of the materials with high Kln

L513-514 prevent vitrification of
L515 clay mineral content should exceed
L520 Figure S26 really deserves a pace in the main manuscript.

L525 only the thermal evolution of
L530-531 systematically evaluating 60
L537 Indeed the broader firing
L538-539 which have been often overlooked previously.
Fig 7 A pattern does emerge howeve re when looking at Fig 7c.the strongest correlations are for 1200 C, where 4 of the 5 are strong (>|0.75|). A lower temperatures the correlations are lower: Modestly strong (>0.5) to strong for 3 of 5 at 1050 C and 4 of 5 at 900 C. At 1330 C the correlations are weak or modestly strong.
L551 importance of temperature-combined factors
L554-555. Statement true for temperature-combined relationship only. Individual temperatures show this relationship to be strong at 1200 C, moderately strong at 900 and 1350 Cand weak at 1050 C. Recommend use of a consistent statistical language to define relationships. For example a strong correlation as having an r^2>|0.75|, moderately strong (|0.5| <r^2<|0.75|) and an r^2<|0.5| as weak would seem appropriate.
L563 Suggestion. is also weakly positive at 0.3
L563-566 Sentence needs reworking. Could be taken to imply that mullite contributes more strength which is not what is meant.
L566 Support this observation,
L569-570 Authors should provide the standard deviations for nano-indentation strength values
L576-577 play dominant roles

L578 areco-dominant (Figure
L579 of inter-related factors

L584 ? term ground breaking? From a science viewpoint, there is nothing "groundbreaking" of applying the reduction and recombination approach to evaluate particular component contributions. From an industry viewpoint this is probably groundbreaking in that sense.

L736-740. Authors should point to evidence that exclusion of intermediate ratios did not compromise. Otherwise why mention that they were excluded from analysis to reduce uncertainty?

Supporting Information
L36 sodium hexametaphosphate is not a surfactant. It is a dispersant. It promotes uniform dispersion by preventing agglomeration (via like charge repulsion)
L47- Purification.
Clarification needed. The separation of kln from ilt is not clearly described. Were the source materials of ilt and kln separate and different from the brick making source materials? If so this is not clear.
L121-123 Unclear. Explain why? If all the clay is removed from the source - and not lost - in the separation process why would the ratios differ? Is this because the source materials were different for each of the three components – Kln, Ilt and Hp? Again not clear if that was the case.
Figure S8 3D images show some artifacts due to either beam hardening or shadowing. The upper segments should be removed for the purpose of publication.
Figure S11 based on 11(B), 11(A) could have another separating line along the middle left where Ilt seems concentrated relative to Kln.
L272-273 (Figure S12 caption). Partial vitrification of. Alternatively could state show partially vitrified Kln
L277 (Figure S13 caption). needles
L280 (Figure S14 caption) Photos
L283 (Figure S15 caption) images …. microscopy showing growth
Figures S16-S25 If 10% corundum added, how does the amount quantify to higher levels? Shouldn't the totals all be normalized to the amount of corundum added as an internal standard? This correction would necessarily reduce the total to < 100% and, since XRD only "sees" the crystalline phases, the residual could be ascribed from difference to 100% to amorphous or glassy phases.
Figure S26 This is an excellent descriptive chart for decision making – it should be part of the main manuscript.
Figure S28 Error bar estimates for the nanoindentation hardness values would be appropriate.

Version 2:

Reviewer comments:

Reviewer #3

(Remarks to the Author)
I have read through all the responses to my (constructive) criticisms, suggestions and corrections and am satisfied that the authors have amended the manuscript or provided rational justification for any decision to not amend. This is a fine manuscript and is acceptable for publication in Nature Communications.

Open Access This Peer Review File is licensed under a Creative Commons Attribution 4.0 International License, which permits use, sharing, adaptation, distribution and reproduction in any medium or format, as long as you give appropriate credit to the original author(s) and the source, provide a link to the Creative Commons license, and indicate if changes were made.
In cases where reviewers are anonymous, credit should be given to 'Anonymous Referee' and the source.
The images or other third party material in this Peer Review File are included in the article's Creative Commons license, unless indicated otherwise in a credit line to the material. If material is not included in the article's Creative Commons license and your intended use is not permitted by statutory regulation or exceeds the permitted use, you will need to obtain permission directly from the copyright holder.
To view a copy of this license, visit https://creativecommons.org/licenses/by/4.0/

Comments from Reviewer 1

Q1*: "10× ASTM" strength framing. The comparison uses button specimens (Ø40 × 12 mm) in compression rather than standard brick units, so direct benchmarking to ASTM C62 unit strengths risks a geometry/size-effect bias. Please justify this equivalence or rephrase the claim conservatively; also report variability.*

Response: Thank you - this is a valid concern. Our compression results were obtained from button specimens (Ø40 × 12 mm), not standard brick units, so a direct, quantitative comparison to ASTM C62 unit strengths without considering size effect can be misleading. In fact, brick buttons were used here primarily for two reasons: 1) 60 formulations were prepared, and button specimens significantly reduce material consumption while enabling consistent, high-throughput screening, and 2) this is a common practice used by local Australian brick manufacturers for product development.

To ensure the button data can be interpreted in the context of full-size units, we performed a dedicated size-effect calibration using a commercial brick clay. Full-size bricks (230 × 110 × 75 mm) and button specimens (Ø40 × 12 mm) were produced under identical compaction conditions (0.62 MPa and 1 min dwell), then dried (110°C, 24 h) and fired (900, 1050, 1200, 1350°C) before compression test. Across these paired tests, full-size bricks consistently exhibited lower strengths than buttons, and we obtained an empirical conversion factor of approximately 0.85 (i.e., $\sigma\_full \approx 0.85 \cdot \sigma\_button$). This reduction is logically expected because thicker specimens are more difficult to sinter uniformly, leading to heterogeneity in densification and greater residual porosity and internal defects.

We have incorporated this calibration into the manuscript, used it to temper the ASTM comparison (now presented as an approximate, size-adjusted context of 8.5× ASTM for the strongest brick). These details can be seen in *Abstract*, *Section 2.3.4*, and *Section 3* on pages 2, 19, 20, and 24 respectively.

Q2*: CS-mullite negative correlation. The statistical signal is interesting, but p-values/CI and sample counts per regression are not reported. Please provide n and statistics for each correlation and clarify whether fits pool all temperatures or are stratified (Figure 7 mentions both views).*

Response: Thank you for the comment. We agree that the relationships among different brick properties should be interpreted in the context of sample size, confidence level, and whether the analysis is temperature-stratified or pooled. We therefore revised the manuscript to explicitly describe the two used analyses and to report the corresponding *n* and statistical significance.

First, correlations of water absorption, apparent porosity, and bulk density with compressive strength at each firing temperature (Figure 7c) were calculated using all formulations available (*n* = 15, Table 1). For amorphous and mullite contents, temperature-stratified correlations were restricted to the three Kln/(Kln+Ilt) endmember/midpoint compositions (0, 50, and 100%), excluding the 33% and 66% mixtures (*n* = 9 at each firing temperature), to reduce uncertainty; since the intermediate ratios were expected to follow the same monotonic trend, this selection does not compromise the accuracy or comparability of the correlation analysis.

Second, pooled correlations (Figure 7d) were assessed across all temperatures using *n* = 39 data points: the 0, 50%, and 100% at four temperatures (36 samples), plus three additional samples (20/33, 35/33, and 50/33) at 1350°C, for which phase analysis was also available.

All tests used a 95% confidence level, and *n* numbers and p-values have been provided in Table S4 and Figure S27 in revised Supporting Information.

Q3*: Illite “melts” at 1050 °C. Main text states pure Ilt “melts” at 1050 °C, while SI maps indicate Ilt is nearly fully melted at 1200 °C with Kln still largely unmelt. Consider softening to “intense vitrification by ~1050-1200 °C” for consistency across sections.*

Response: Thank you for this careful observation. We re-checked the SEM images and confirm that pure illite (Ilt) shows only incipient softening at 1050°C (rounded grain edges and a slightly smoother surface), while it becomes fully vitrified at 1200°C. In contrast, kaolinite (Kln) only partially vitrified at 1200°C, with clear crystal-melt coexistence and corrosion boundaries. Following your suggestion, we replaced “melt” with “(intense) vitrification” and revised the wording to indicate that Ilt undergoes strong vitrification over ~1050-1200°C rather than “melting at 1050°C”. These changes have been implemented in Figure 1, Figure S7, and the corresponding text in *Section 2.2* (page 8).

Q4*: Over-firing/closed-pore mechanism. The explanation is plausible and the SEM evidence compelling; adding quantitative porosity (open vs closed) via MIP or micro-CT on key points (e.g., 50/0 at 1350 °C) would make the mechanism and the ~10% expansion claim rock-solid.*

Response: Thank you for the suggestion. Following your recommendation, we performed micro-CT on two representative specimens (50/0 fired at 900°C and 1350°C) to quantify open versus closed porosity with increasing firing temperature. For 50/0/900, the open and

closed porosities are 9.85% and 0.12%, respectively; for 50/0/1350, they change to 1.55% and 3.40%. The marked increase in closed porosity (0.12% to 3.40%), together with the reduction in open porosity, supports progressive pore closure during densification and subsequent inflation of sealed pores under over-firing conditions, consistent with the observed expansion at 1350°C. We have incorporated this micro-CT evidence in *Section 2.2* (page 8) and added the corresponding images/quantification in Figure S8 in Supporting Information.

Q5*: Replicates and error bars. The manuscript shows 72 dried buttons in SI (suggesting 60+ specimens), but the number tested per condition for CS/WA and the spread (SD/SE) are not reported. Please specify replicates and plot error bars/CI for CS, WA, density, and phase fractions.*

Response: Thank you for this suggestion. Figure S14 shows 72 dried buttons only to illustrate the colour/appearance of green bodies across formulations. The reason 72 buttons are shown is that we used a central composite design, which includes additional points around the centre of the design space (hence more samples than the 60 formulations).

For the actual property dataset, ~390 specimens were produced in total to complete all tests. Compressive strength was measured on 72 fired button specimens, with the entire set produced and tested in two independent batches (144 tests total); the reported compressive strength for each formulation/condition is the mean of the two repeats. Firing shrinkage, water absorption, and bulk density were measured for all 60 formulations using two replicate specimens per formulation (120 specimens total). In response to reviewer requests, we also measured apparent porosity for all 60 formulations, again using two replicates per formulation, and report the averaged values.

We have added these replicate details to *Section 4* (page 27) and plotted error bars (mean ± SD) for compressive strength, firing shrinkage, water absorption, apparent porosity, and bulk density in Figure 1 (page 7), Figure 4 (page 16), and Figure 5 (page 18). For quantitative phase fractions, since only one specimen per selected condition was analysed, to demonstrate reliability, we have made sure that the Rwp value from each Rietveld refinement is below 10% (Rwp is a statistical indicator used to assess the quality of a Rietveld refinement fit in X-ray diffraction analysis; a lower Rwp value indicates a better agreement between the observed experimental data and the calculated theoretical model), and explain this criterion in *Section 4* on page 27.

Q6*: Firing schedule. Figure S25 is schematic; please provide exact ramps, soaks, cooling used for firing the buttons (time–temperature history), as this strongly affects phase assemblage/porosity.*

Response: Thank you for this comment. The firing schedule has now been detailed in *Section 4* on page 25.

***Q7**: Pressing details. State compaction pressure, dwell time and release protocol for the hydraulic pressing step (now "hydraulic compression" only).*

Response: Thank you for this comment. The samples were uniaxially compacted at a load of 5 tons for 1 min using an RK 30-ton hydraulic press. After the dwell period, the applied load was released immediately following standard laboratory practice. This information has now been added to *Section 4* on page 25.

***Q8**: Statistics for correlations. Report n, p-values, and confidence intervals for Pearson r/R^2 (Figure 7), ideally with temperature-stratified analyses and a multiple-testing note.*

Response: Thank you for this suggestion. Two complementary correlation analyses were conducted in this study to quantify relationships among the measured brick properties. First, we examined temperature-stratified relationships between compressive strength and key physical parameters at each firing temperature (Figure 7c). Second, we evaluated pooled correlations among multiple properties across the full temperature range (Figure 7d).

For the temperature-stratified analysis, Pearson's correlations between compressive strength and water absorption, apparent porosity, and bulk density were computed using all available formulations at each firing temperature (n = 15; Table 1). For correlations involving phase contents (amorphous and mullite), the analysis was restricted to the three Kln/(Kln+Ilt) endmember/midpoint compositions (0%, 50%, and 100%; n = 9 per temperature), and the 33% and 66% mixtures were excluded to minimize uncertainty in phase quantification. Because these intermediate compositions are expected to follow the same monotonic compositional trend, their exclusion does not affect the interpretability or comparability of the correlation results.

For the pooled analysis, correlations were calculated across all temperatures using n = 39 data points: the 0%, 50%, and 100% compositions measured at four firing temperatures (36 samples), plus three additional samples (20/33, 35/33, and 50/33) at 1350°C for which phase analysis was also available. All confidence intervals are reported at the 95% level. The corresponding p-values for both the temperature-stratified and pooled analyses are summarized in Table S4 and Figure S27 (Supporting Information), and the statistical approach is further explained in *Section 4* (page 28).

***Q9**: The temperature-dependent role reversal (Kln helpful at 900 °C; Ilt at 1050–1200 °C; mixed at 1350 °C) is well argued and consistent with flux content (K-rich Ilt) and observed vitrification differences. Consider adding a compact decision chart mapping firing window*

→ optimal Kln/Ilt (you already outline this in §2.4, including Fe_2O_3 threshold guidance ⩾ 6.5%).

Response: Thank you for this helpful suggestion. We agree that a compact decision chart linking the firing window to the recommended Kln and Ilt contents would make the temperature-dependent role reversal easier to follow. Accordingly, we have added a decision chart that also incorporates the Fe_2O_3 threshold guidance; given the limited space in the main text, it is provided as Figure S26 in the Supporting Information.

***Q10**: Quartz/cristobalite effects at 1200 °C linked to CS dips are reasonable; a brief note on thermal cycling implications (β↔α cristobalite) would help contextualise durability claims.*

Response: Thank you for your suggestion. The β↔α cristobalite transformation during heating and cooling involves a sudden volume change, which can generate internal stresses and trigger microcracking. Over repeated cycles, these microcracks can accumulate and grow, gradually weakening the brick structure and compromising its durability. We have added a brief note on this thermal-cycling implication to better contextualise the compressive strength reduction observed at 1200°C and to clarify the durability discussion in the revised manuscript (*Section 2.3.4*, page 19).

***Q11**: The conclusion that glass fraction (amorphous) matters more than mullite for CS in coarse brick bodies is persuasive; still, a small nanoindentation/phase-specific modulus dataset or fractography on selected mixes would substantiate the micro-mechanics behind Figure 7.*

Response: Thank you for this valuable suggestion. In our samples, mullite occurs as very fine crystals (typically < 0.5 μm in width), making it difficult to position an indent entirely within a single mullite crystal without also sampling the surrounding glassy/amorphous matrix. Therefore, rather than attempting "pure-phase" indentation on mullite, we performed statistical nanoindentation on the amorphous matrix in regions that are either rich in, or deficient in, embedded mullite crystals.

Nanoindentation tests were conducted on 35/33/1350 and 35/100/1350. For each sample, two amorphous-matrix regions (mullite-deficient and mullite-bearing) were indented using 7 × 7 arrays (49 indents per region), and the mean values were calculated. The results show consistently lower hardness in mullite-bearing amorphous regions than in mullite-deficient regions (Figure S28, Supporting Information). Specifically, for 35/33/1350, the mean hardness was 9.50 GPa (mullite-deficient) versus 8.64 GPa (mullite-bearing), and for 35/100/1350 it was 9.58 GPa versus 7.71 GPa, respectively. Since lower hardness generally indicates reduced local mechanical resistance, these observations support our interpretation

that the glassy/amorphous matrix is a primary contributor to compressive strength development in coarse brick bodies.

We have added the corresponding discussion in *Section 2.5* (page 23) and included the new nanoindentation figure as Figure S28.

***Q12**: Use consistent terminology for Ilt behaviour ("vitrifies/intensely vitrifies" rather than "melts") across main text and SI.*

Response: Thank you for this kind reminder. We have carefully checked the manuscript and Supporting Information and replaced "melt/melting" with "vitrify/vitrification" to ensure consistent terminology on pages 8, 11, 14, and 22, and the Supporting Information.

***Q13**: Consider a table comparing your best mixes with typical industrial ranges (CS, WA, density) to emphasise practical relevance (references already cited in Intro/Discussion).*

Response: Thank you for this valuable suggestion. We have now added a comparative table that benchmarks the performance of our best-performing formulations against the typical property ranges reported for bricks manufactured from industrial brickmaking clays worldwide (compressive strength, water absorption, and bulk density). This comparison shows that our formulations consistently achieve higher compressive strength and bulk density together with lower water absorption across firing temperatures, highlighting their practical relevance. These results further demonstrate that, through scientifically guided formulation optimisation, clay-based systems can develop stronger bonding and improved densification at the same firing temperature. In turn, this offers clear engineering benefits - for example, achieving a target strength at a lower firing temperature, thereby reducing energy demand and production cost.

Due to space limitations in the main text, we have included the full comparison in Table S3 (Supporting Information) and added the corresponding discussion in *Section 2.3.4* (page 20).

Comments from Reviewer 2

***Q1**: The criteria used by the authors to define the independent variables are not entirely clear to me. For instance, in the case of total clay mineral content, why is Hp included in the ratio of clay minerals, if the clay minerals were already separated? Moreover, why is the Kln-to-clay ratio considered but not the Ilt-to-clay ratio? I think a more detailed explanation could be given.*

Response: Thank you for raising this important concern. In our study, the KIQF system is designed to represent a simplified but realistic brickmaking raw-material system composed of three adjustable components: kaolinite (Kln), illite (Ilt), and heavy particles (Hp). In KIQF, the total clay minerals are defined as the sum of Kln+Ilt, whereas Hp represents the non-clay mineral fraction typically present in industrial brick clays, mainly including quartz, feldspar, and Fe-bearing minerals (Supplementary Note 1). Therefore, the total clay mineral content is expressed as (Kln+Ilt)/(Kln+Ilt+Hp), because Kln+Ilt+Hp constitutes the complete KIQF raw-material system.

Regarding the second point, we used the Kln-to-clay mineral ratio, Kln/(Kln+Ilt), as an independent variable because Kln and Ilt together comprise the clay mineral fraction (20%, 35%, and 50%) in KIQF. The corresponding Ilt-to-clay mineral ratio, Ilt/(Kln+Ilt), is mathematically complementary (i.e., 1-Kln/(Kln+Ilt)). For example, when Kln/(Kln+Ilt) is 0, 33.3%, 50%, 66.6%, and 100%, Ilt/(Kln+Ilt) is automatically 100%, 66.6%, 50%, 33.3%, and 0, respectively. Thus, including both ratios would be redundant and unable to provide additional independent information.

We have added these clarifications in *Section 2.1* of the revised manuscript (pages 5 and 6). We hope this revision addresses your concern and improves clarity.

***Q2**: A porosimetric analysis of the samples is lacking. Throughout the manuscript, the authors discuss the influence of kaolinite and illite on porosity. Including this analysis would substantially strengthen the study by testing whether the higher proportion of kaolinite or illite affects the total porosity of the bricks.*

Response: Thank you for this excellent suggestion. Recognising the central role of porosity in controlling brick performance, we have added an apparent porosity measurement for all 60 sets of fired brick specimens using a He pycnometer, following the procedure reported by Santos, Vaz [1]. For each formulation, two specimens were tested, and the mean apparent porosity was reported as the final value; the corresponding standard errors are presented in Figure 4. The detailed experimental protocol has also been added to Supplementary Note 10.

Building on these new data, we now provide a more quantitative discussion of how apparent porosity varies with the kaolinite-to-clay mineral ratio, total clay mineral content, and firing temperature in *Section 2.3.3* (page 15). In addition, apparent porosity is explicitly considered

when discussing compressive strength (*Section 2.3.4*) and the role of firing temperature (*Section 2.4*), as shown on pages 17 and 21 of the revised manuscript. Finally, the porosity dataset has been incorporated into the correlation analysis in Figure 7c and 7d within *Section 2.5* (pages 22 and 23).

Overall, in response to the reviewer's recommendation, we have conducted a comprehensive, quantitative evaluation of porosity across formulations, analysed its trends and underlying mechanisms, and clarified its relationships with other key brick properties, thereby providing direct data support for our porosity-related interpretations throughout the manuscript.

Q3*: Although a large number of brick formulations were studied, it is unclear whether all of them were analyzed using the same techniques. For example, the thermal dilatometry data (Figure 1) are reported only for mixtures with a total clay content calculated at 35%, whereas the supplementary materials (Figure S6) include mixtures with a kaolinite-to-clay minerals ratio at 50%. Likewise, for electron microscopy, images are provided only for formulations with a kaolinite-to-clay minerals ratio at 35%.*

Response: Thank you for raising this important point. In current study, all 60 formulations were assessed using the same set of core performance tests, while a smaller number of representative formulations were selected for more time-intensive characterization aimed at explaining mechanisms.

Specifically, compressive strength, firing shrinkage, water absorption, apparent porosity, and bulk density were measured for all 60 fired brick formulations. We applied these tests across the full matrix because they form the basis for comparing formulations and identifying optimal mixes. By contrast, thermal dilatometry and SEM were conducted on selected samples rather than the entire set. These techniques were used to interpret how the three key variables in our design - kaolinite-to-clay mineral ratio, total clay mineral content, and firing temperature - affect thermal behaviour and microstructural evolution, which then helps explain the observed macroscopic trends. Given the large number of specimens, extending dilatometry and SEM to every formulation would substantially increase experimental workload and generate a volume of data that cannot be accommodated in the paper without reducing readability, while not changing the main conclusions.

For thermal dilatometry, we initially tested five formulations chosen to isolate the effects of composition. The series 35/0, 35/50, and 35/100 was used to examine the influence of the kaolinite-to-clay mineral ratio at a fixed total clay content (35%), whereas 20/50, 35/50, and 50/50 was used to examine the influence of total clay mineral content at a fixed kaolinite ratio (50%). For SEM, the sampling strategy followed the same logic. To examine temperature effects, we analysed the 35/50 formulation fired at 900°C, 1050°C, 1200°C, and 1350°C. To examine the effect of total clay mineral content, we analysed 20/0, 35/0, and 50/0 fired at

1350°C, and to examine the effect of kaolinite-to-clay mineral ratio, we analysed 35/0, 35/50, and 35/100 fired at 1350°C.

To remove any remaining ambiguity and strengthen the rigor of the mechanistic dataset, we have now added the missing dilatometry results for Kln/(Kln+Ilt) = 33% and 66% into Figure 1, together with the corresponding discussion in *Section 2.2* (Page 8). We have also included SEM images of 35/33/1350 and 35/66/1350 in Figure S9 (Supporting Information). As expected, these additional data show the same evolution in expansion/shrinkage behaviour and microstructural development as the original subset, and therefore strengthen - rather than alter - our interpretation. We have now made these choices explicit in *Section 4* (pages 26 and 27) of the revised manuscript to avoid any ambiguity about which techniques were applied to the full set and which were applied to representative samples. We hope this approach maintains full comparability for key properties while using targeted characterisation to support the mechanistic interpretation without unnecessarily increasing the manuscript length.

Q4*: "given the annual production of over 1,500 billion bricks". Is this data referring to worldwide production, and to which year does it correspond?*

Response: Thank you for this comment. 1,500 billion is the global annual clay brick production. This number was initially reported by Weyant, Athalye [2]. However, unfortunately, no information about the production year was released. We have updated our statement and the reference in Page 3 of the revised manuscript.

Q5*: Line 126: Please indicate "kaolinite–illite–quartz–feldspar" here, as this is the first occurrence of the abbreviation KIQF.*

Response: Thank you for pointing this out. The full name of KIQF is provided at its first appearance in the manuscript (last paragraph of the Introduction section); please see page 4 for details.

Q6*: Mullite Formation section (lines 295–323). Some additional references could be incorporated here. There are numerous studies describing mullite formation from metakaolinite in different ceramics.*

Response: Thank you for this suggestion. In response, we have incorporated additional references ([39-44]) that discuss mullite formation during the thermal evolution of kaolinite and illite, and we have compared our results with those reported in the literature. These revisions have been made in the Mullite Formation section on pages 13 and 14 of the revised manuscript.

Q7*: Figures S1, S2 and S3: Are Hp, Kln and Ilt samples here raw or purified? Including a comparison of the XRD results before and after purification would significantly improve the clarity of this section.*

Response: Thank you for this suggestion. Figures S1-S3 present the XRD results of Hp, Kln, and Ilt after purification. To improve clarity, we have now added the XRD patterns of the raw (unpurified) clay mix (Hp), Kln, and Ilt samples and provide a direct before-after comparison: the raw bulk XRD patterns are included in Figure S1, and the corresponding clay-size fraction XRD patterns are provided in Figure S2. In addition, we have carried out a quantitative phase analysis for the raw clay mix (Hp), Kln, and Ilt samples and included the results in Figure S3 to demonstrate the effectiveness of the purification procedure (*Section 2.1*, page 5).

References

1. Santos TP, Vaz MF, Pinto ML, Carvalho AP. Porosity characterization of old Portuguese ceramic tiles. *Constr Build Mater* **28**, 104-110 (2012).

2. Weyant C, *et al.* Emissions from South Asian Brick Production. *Environmental Science & Technology* **48**, 6477-6483 (2014).

Comments from Reviewer #2

The authors have properly addressed all comments and have included new data into both the manuscript and the supplementary material, thereby strengthening the study. I consider that the manuscript is now suitable for publication.

Response: We sincerely thank the reviewer for the positive evaluation of our revised manuscript. We greatly appreciate the reviewer’s thoughtful and constructive comments, which have helped us improve the scientific rigor, clarity, and overall quality of the study.

Comments from Reviewer #3

Q1: *Regarding the compressive strength results there seem to be some misinterpretations. For example L441-443 – the authors state that at intermediate temperatures (1050 and 1200C) vitrification densified the bricks so that higher clay mineral content increases CS, but this is only true at low Kln contents (< 35% Kln for the most part).*

Response: Thank you for this correction. We agree that the positive relationship between total clay mineral content and CS at 1050°C and 1200°C is primarily observed in Ilt-rich formulations. The text on page 19 has been revised accordingly to restrict this interpretation.

Q2: *In the section on correlations of various parameters with compressive strength – there needs to be more consistent use of statistical language for example defining a strong correlation as having an r^2>|0.75|, moderately strong (|0.5|<r^2<|0.75|) and an r^2<|0.5| as weak would seem appropriate for the complexity of the inter related interactions between the various known parameters. Obviously the authors can choose their own boundaries.*

Response: Thank you for this helpful suggestion. We have adopted consistent statistical terminology throughout the revised manuscript, classifying correlations as strong ($| r | > 0.75$), moderately strong ($0.50 < | r | \leq 0.75$), or weak ($| r | \leq 0.50$). Pearson's correlation coefficient r, rather than r^2, is used because the analysis considers both the strength and direction of each relationship. These definitions are provided in the caption of Figure 8 on page 24, and the relevant discussion has been revised on pages 23-25.

Q3: *Further, the authors need to make clear where they use temperature-combined data vs temperature separated data. The former tend to mask correlations whereas the latter tend to highlight them. For example the statement on L554-555 regarding the correlation between bulk density and CS is true only for temperature-combined data. For the temperature separated data, the relationship is strong at 1200 C , moderately strong at 900 and 1350 C and only weak at 1050 C.*

Response: Thank you for this clarification. We have revised the text on pages 23-25 to clearly distinguish correlations calculated from the temperature-combined dataset from those calculated separately at each firing temperature. The statement regarding bulk density and CS now specifies that the lack of statistical significance applies only to the combined dataset, while the temperature-specific correlation strengths are discussed separately.

Q4: *The authors do point out that there may be confounding interactions that influence the "scatter" in the data, such as sintering-induced mullite formation, over- or under-vitrification of illite etc., which undoubtedly are at play – but another experimental error that could be considered may be – inadvertent differences in the fines content of the Hp between various mixes. Such considerations may also explain some of the lack of correlations.*

Response: Thank you for this valuable suggestion. We agree that inadvertent variation in the fine-particle content of Hp among different mixes could contribute to data scatter. Such variation may affect particle packing, sintering, and pore development, thereby weakening the observed correlations. This potential source of experimental variability has now been acknowledged on page 25 of the revised manuscript.

Q5: *In the Discussion – which is otherwise actually quite informative regarding the nuances of the dataset – some further aspects of the (non) universality of the results should be discussed in light of the given brick making material studied herein – in which the authors comprehensively disassembled it into three main components and then re-assembled these three components into a wide range of compositions- ranging from low total clay mineral to high total clay mineral and from no kaolinite to no illite (where kaolinite and illite were the only clay minerals identified and present) – given the known impacts of chemistry,*

crystallinity and particle size of clay minerals on their reactivity, thermal stability and etc. For example, would on expect a deposit chosen for brick making in e.g. Chechia to behave more or less alike with respect to mullite formation and/or dilatancy / vitrification / pore formation? For example the both the kaolinite and illite in this material had relatively low iron contents, so could the authors comment on how a less weathered material – with greater iron content within the structure of the layered clay minerals – behave with respect to mullite formation?

Response: Thank you for this insightful comment, which raises an important question regarding the broader applicability of our findings. Although the KIQF system covers a wide compositional range, the Kln and Ilt examined were derived from a specific source material. Therefore, the quantitative relationships established here may vary for clay minerals with different chemical compositions, structural defects, crystallinities, particle sizes, and accessory minerals. This sensitivity is supported by our previous study, in which bricks containing high-defect kaolinite exhibited 7.1% higher compressive strength at 950°C than those containing low-defect kaolinite, associated with greater spinel formation and a finer pore structure.[1] However, at higher calcination temperatures, such as 1150°C, no appreciable difference in compressive strength was observed between samples prepared using low- and high-defect kaolinites.

We have therefore clarified in the Discussion (page 27) that kaolinite-illite-rich clays from other deposits may follow similar general mechanisms but exhibit different phase-transformation temperatures, pore-development behavior, and optimum compositions. We have also expanded the "Mullite Formation" section (page 14) to explain that structural Fe may participate in mullite nucleation and be incorporated into its structure, whereas excess Fe may form hematite or Fe-Ti phases. Accordingly, less-weathered clays with higher structural Fe contents may exhibit different mullite formation behavior. We agree that systematically investigating kaolinite and illite from different geological deposits is both important and necessary, and we have identified this as a key direction for future work.

Q6: *L40-41 two of the most critical clay minerals for brick making –*

Response: Thank you for the correction. The corresponding text has been revised. Please see page 2 for details.

Q7: *L42 strengths*

Response: Thank you. The text has been corrected on page 2 of the revised manuscript.

Q8: *L43 methods*

Response: Thank you for pointing this out. The text has been revised accordingly. Please see page 2 for details.

Q9: *L44 uncovered a*

Response: Thank you for the correction. The text has been revised accordingly. Please see page 2 for details.

Q10: *L46 responses*

Response: Thank you. The text has been revised accordingly. Please see page 2 for details.

Q11: *L47 phases; and over*

Response: Thank you. We have revised the text on page 2.

Q12: *L59 oxyhydroxides(^2,3) have made … understand the underlying*

Response: Thank you for the correction. The text has been revised accordingly. Please see page 3 for details.

Q13: *L60-61 Unclear rationale. Suggest adding to end … production of homogeneously performing product.*

Response: We appreciate this suggestion. We have added the recommended rationale to clarify the text (please see page 3).

Q14: *L68 high – at approximately*

Response: Thank you for catching this. The wording on page 3 has been updated to "at approximately" as advised.

Q15: *L69 delete etc.*

Response: Agreed. We have removed "etc." from this sentence on page 3 of the revised manuscript.

Q16: *L70-72. Resource use and availability of suitable virgin resources are also big issues with materials for construction worldwide.*

Response: Thank you for this important comment. We have revised the text to acknowledge that defective brick production not only causes energy waste and environmental pollution but also increases the consumption of natural resources and pressure on the availability of suitable virgin raw materials. The corresponding revision can be found on page 3 of the revised manuscript.

Q17: *L74-75 "defects" may be more due to weathering processes resulting in different defects from different parent material (primary factor. For e.g. granitic parent materials the inherent crystallinity and chemical composition of the main mineral constituents will influence how a given weathering regime will affect the clay minerals formed.*

Response: Thank you for this helpful clarification. We agree that the properties of the parent material, including its mineralogy, crystallinity, and chemical composition, determine how it responds to weathering and influence the clay minerals that subsequently form. We have revised the text accordingly on page 3.

Q18: *L75 practice because most (combine sentences)*

Response: Thank you for the suggestion. The two sentences have been combined on page 3 of the revised manuscript.

Q19: *L80 echos*

Response: Thank you for the correction. The text has been revised accordingly. Please see page 3 for details.

Q20: *L105 roles of the clay minerals Kln and Ilt in*

Response: Thank you for the suggestion. The text has been revised accordingly. Please see page 4 for details.

Q21: *L121 Precisely tuning clay minera's./// Phrase here is unclear. If the authors mean defining the composition of Kln and Ilt via purification methods then simply state that. Don't use jargon.*

Response: Thank you for this clarification. We agree that the original phrase "precisely tuning clay minerals" was unclear. We have replaced it with "precisely controlling the relative proportions of Kln and Ilt through mineral separation and purification" to explicitly describe the approach used in this study. The corresponding revision can be found on page 5 of the revised manuscript.

Q22: *L123 the common coexistence*

Response: Thank you for this comment. The text has been revised to "the common coexistence" on page 5 of the revised manuscript.

Q23: *L124 making their separation*

Response: Thank you for the correction. The text has been changed to "making their separation". Please see page 5 for details.

Q24: *L126 purity being rare*

Response: Thank you for the suggestion. We have revised the sentence to clarify that exceptionally pure natural clay minerals are rare because they commonly coexist with other mineral phases formed through the weathering of different parent rocks. The corresponding revision can be found on page 5 of the revised manuscript.

Q25: *L134-136 contradictory? Just mentioned above (L125-126) they are rarely high pure. Possibly the authors mean here that they applied procedures to isolate Kln and Ilt as implied from the next sentence.*

Response: Thank you for this comment. We did not intend to suggest that the starting Kln and Ilt were naturally occurring high-purity minerals. Rather, they were isolated from natural clay sources and further purified in the laboratory. We have clarified this point on page 5 of the revised manuscript and in Supplementary Note 2 of the revised Supporting Information.

Q26: *L139 potential issues with values see comment on XRD (Figs S16-S24) and use of corundum*

Response: Thank you for raising this important point. We agree that the crystalline-phase contents should be calibrated against the known amount of corundum, with the remaining fraction assigned to the amorphous/glassy phase.

Figures S16-S25 show the raw TOPAS refinement outputs. In these outputs, the identified crystalline phases and corundum are automatically normalized to 100%. Because the amorphous phase is not included in this normalization, the refined corundum contents range from 12.08% to 23.86%, despite its known content of 10 wt%. Therefore, the phase percentages displayed in Figures S16-S25 represent relative proportions within the crystalline fraction and are not the final phase contents used in the manuscript.

For the final quantitative results, the refined crystalline-phase contents were corrected using the known 10 wt% corundum content. Specifically, the corundum content was restored to 10 wt%, and the sum of all corrected crystalline phases was less than 100%. The amorphous/glassy content of the corundum-spiked mixture was then calculated as the difference between 100% and the sum of all corrected crystalline phases, including corundum. Finally, corundum was excluded, and the remaining crystalline and amorphous contents were converted to the original sample basis and normalized to 100%. These corrected values, rather than the raw values shown in Figures S16-S25, are those reported in the manuscript, for example, on page 5 and Figure 3.

This calculation is fully consistent with the internal-standard procedure suggested by the reviewer. We apologize that the distinction between the raw refinement outputs and the corrected phase contents was not sufficiently clear. The complete calculation procedure has now been added to Supplementary Note 6, and the captions of Figures S16-S25 have been revised to clarify that they show the raw TOPAS refinement outputs.

Q27: *L143 size distributions*

Response: Thank you for point this out. We have changed "distribution" to "distributions" on page 5 of the revised manuscript.

Q28: *L144 replace performed with measured*

Response: Thank you. The text has been revised accordingly. Please see page 5 for details.

Q29: *L146-149 ? Fig S5 doesn't show this at all. S5a shows a two overlapping (normal) distributions for Kln centered near 1 micron and minor (<~5%) larger particles than 10 um and virtually all < 30 um (cumulative %). For Ilt S5b shows a normal distribution centered near 4.5 um. Also most of the HP (~75%) has particles < 50 um with most of those averaged near 5 um.*

Response: We sincerely thank the reviewer for this valuable comment. We agree that the volume-weighted mean particle size, $D_{[4,3]}$, did not fully represent the particle size distributions shown in Figure S5. $D_{[4,3]}$ is calculated as

$$D_{[4,3]} = \sum n_i\, d_i^4 / \sum n_i\, d_i^3$$

where n_i is the number or number frequency of particles in size class i, d_i is the representative particle diameter of that size class, and i denotes the individual particle-size class. Because particle volume scales with the cube of particle diameter, this parameter is highly sensitive to small amounts of coarse particles. For example, one 100 μm particle has

the same volume as $(100/1)^3$ = 1,000,000 particles of 1 μm diameter. Therefore, the minor coarse-particle fractions in Kln and Hp resulted in $D_{[4,3]}$ values substantially larger than their median particle sizes (D_{50}) apparent in Figure S5. In contrast, the more symmetric particle size distribution of Ilt resulted in a D_{50} value (3.94 μm) relatively close to its $D_{[4,3]}$ value.

Considering this limitation, we have replaced the $D_{[4,3]}$-based description with the cumulative particle size parameters D_{10}, D_{50}, and D_{90}, which are more commonly used to describe broad particle size distributions in the published literature. The revised text can be found on page 6 of the revised manuscript. The D_{10}, D_{50}, and D_{90} values of Kln, Ilt, and Hp have also been added to Table S1 of the revised Supporting Information.

Q30: *L162 Suggest making differentiating the clay minerals from the heavy particles in this manner particularly*

Response: We appreciate the reviewer’s insightful suggestion. We agree that the original statement did not explicitly clarify how the selected variables in the KIQF system correspond to the compositional variability of natural brick clays. In the revised manuscript, we have clarified that variations in natural brick clays mainly arise from two aspects: 1) changes in the relative proportions of clay minerals, particularly the Kln/Ilt ratio, and 2) changes in the proportion of heavy particles (mainly quartz) relative to clay minerals. Accordingly, these two variations were represented by the Kln/(Kln+Ilt) ratio and total clay mineral content [(Kln+Ilt)/(Kln+Ilt+Hp)] in the KIQF system, respectively. This clarification highlights the rationale behind the selection of independent variables in this study. Please see page 6 for details.

Q31: *L164 Suggest optimization practices typically used in industrial*

Response: Thank you for this suggestion. The text has been revised accordingly on page 6 of the revised manuscript.

Q32: *L181 Fig 1a Authors should indicate the processes occurring in stages 1 and 2 in Figure 1a. Choice of colors not ideal in as it is hard to differentiate the light greens and light blues, particularly with the dotted lines.*

Response: We sincerely thank the reviewer for this helpful comment. In the revised Figure 1a, we have added annotations indicating the dominant processes occurring in Stages 1 and 2, namely the removal of physically adsorbed water in Stage 1 and the progressive thermal expansion of the mineral particles in Stage 2. We have also replaced the light green with a contrasting light peach-orange to make the different datasets easier to distinguish, particularly those represented by dotted lines.

Q33: *L188-190 seems the authors are putting the cart before the horse here. Also seems it would be clearer to state shrinkage rather than expansion decrease? Suggest In stage 3 brick expansion abruptly accelerates rapidly due to conversion of alphato beta quartz near 573C (Fig 1a). This behavior is partially counteracted by dehydroxylation of Kln resulting in its structural collapse and increases with Kln/(Kln+Ilt) ratio. Also...... Kln dehydroxylation temperature is dependent on many aspects of kaolin chemistry and crystallinity, so it seems serendipitous for Kln dehydroxylation to correspond quartz converstion.*

Response: Thank you for this insightful comment. We agree that the original wording obscured the causal sequence. The text has been revised to first describe the expansion caused by the α-to-β quartz inversion near 573°C, followed by the compensating shrinkage associated with kaolinite dehydroxylation and structural collapse.

We also agree that kaolinite dehydroxylation temperature varies with its chemistry and crystallinity. We have therefore limited this interpretation to the present samples. The revised text can be seen on pages 8 and 9 of the revised manuscript.

Q34: *L191 Greater incorporation of Kln can reduce cracking*

Response: Thank you. We have added “greater” in front of “incorporation”. Please see page 8 for details.

Q35: *L192 effect at this temperature because*

Response: Thank you for this comment. The entire paragraph has been rewritten for clarity. Please see the revised text on pages 8 and 9.

Q36: *L192-193 Has the Ilt in this particular brick making material (or surrogate) dehydroxylated by 570C? Highly unlikely. Have the authors conducted TGA to show the dihydroxylation temperatures of the purified Kln and Ilt? Like Kln, ILT dehydroxylation temperatures are dependent on chemistry and crystallinity, but additionally on the occupation of the octahedral sheet. Authors need to acknowledge this fact in their interpretations.*

Response: We thank the reviewer for this important comment. We agree that Ilt in the present material is unlikely to have fully dehydroxylated by 570°C. The *in-situ* high-temperature XRD results in Figure 3k indicate that Ilt dehydroxylates mainly between 600 and 700°C, with only the lower part of this interval overlapping Stage 3 (500-650 °C). Unlike Kln, however, Ilt largely retains its layered structure following dehydroxylation and therefore does not undergo comparable collapse-related shrinkage.[2] We have revised the corresponding

discussion accordingly. We have also clarified that the dehydroxylation temperatures of both Kln and Ilt depend on their chemistry and crystallinity and, for Ilt, additionally on octahedral-sheet occupancy. Therefore, the present interpretation is specific to the samples examined in this study. Please see the revised discussion on pages 8 and 9.

Q37: *L198 metakaolin prior to mullite formation. Comment mullite is a spinel.*

Response: We thank the reviewer for drawing our attention to the ambiguity in the original wording. We have revised the text to "metakaolin prior to the formation of high-temperature crystalline phases" on page 9.

The high-temperature phase evolution of kaolinite remains debated. Following dehydroxylation, metakaolinite transforms through the crystallization of a spinel-type phase accompanied by the formation of amorphous SiO_2. The composition of this intermediate phase is still uncertain and has been proposed to be either γ-Al_2O_3 or an Al-Si spinel.[3]

With further heating, this intermediate phase subsequently evolves into mullite. This process is also described by two competing mechanisms: γ-Al_2O_3 may react with amorphous SiO_2 to form mullite, or the Al-Si spinel may progressively release SiO_2 and evolve toward 3: 2 mullite. These mechanisms are discussed in detail in "Phase Transformation of Kaolinite Clay by Chakraborty (2014)".

We understand that the reviewer may be referring to this mullite-related Al-Si spinel intermediate. Nevertheless, the intermediate spinel-type phase and the final orthorhombic mullite phase are generally treated as crystallographically distinct phases. To avoid ambiguity and accommodate the uncertainty surrounding this transformation pathway, we have adopted the more general expression "metakaolin prior to the formation of high-temperature crystalline phases."

Q38: *L207 ? viscous-phase formation?*

Response: Thank you. The text has been revised accordingly on page 9 of the revised manuscript.

Q39: *L228-229 At most this would be about 3% - is that sufficient when Ilt fully dispersed within Hp? Could this effect be more related to the PSD of the Hp?*

Response: Thank you for this insightful comment. We agree that the increase in the Ilt basal spacing is insufficient to fully explain the measured macroscopic expansion in Figure 1c, particularly when Ilt is dispersed within the Hp matrix.

Our recalculation shows that the $d_{(001)}$ spacing of Ilt increases by approximately 2% after dehydroxylation. Even assuming complete transfer of this crystallographic expansion to the

bulk specimen, the maximum estimated contributions from Ilt would be approximately 0.4%, 0.7%, and 1.0% for the formulations containing 20%, 35%, and 50% Ilt, respectively. These values are consistently lower than the corresponding measured expansions of 1.1%, 1.3%, and 1.6% in Figure 1c.

Moreover, these estimates represent idealized upper bounds because they do not account for the random orientation of Ilt particles, constraint by the surrounding Hp matrix, pore accommodation, or particle rearrangement. The actual contribution of Ilt lattice expansion to the bulk deformation is therefore likely to be smaller.

We consequently agree that the observed expansion cannot be attributed primarily to the increase in the Ilt $d_{(001)}$ spacing. Although the same Hp material was used in all formulations and its intrinsic particle-size distribution remained unchanged, increasing the Ilt content necessarily decreased the Hp proportion and altered the overall particle-size distribution, packing structure, initial porosity, and firing behavior of the mixtures. These formulation-dependent effects may therefore make an important contribution to the measured expansion.

The manuscript has been revised accordingly on page 10. The increase in Ilt $d_{(001)}$ spacing is now presented only as a possible contributing factor, while the potential effects of the overall particle-size distribution and particle packing are explicitly acknowledged.

Q40: *L238 comment on item 3). Seems rather a loss of smaller pores. Data in Fig S8, could show this more effectively, depending on the voxel size - but unclear if a Kln100/Ilt0 image measured?*

Response: Thank you for this comment. We have revised item 3 to “a loss of small pores” on page 10. Although micro-CT data were not collected for the Kln100/Ilt0 sample, pore structures in both Kln100/Ilt0 and Kln0/Ilt100 samples were analysed based on SEM images in Figure S9 using ImageJ software. The calculated average pore area increased from 17.4 μm^2 for Kln100/Ilt0 to 2804 μm^2 for Kln0/Ilt100, supporting a preferential loss of smaller pores with increasing Ilt content.

Q41: *L260 vitrification at 900C.*

Response: Thank you. The text has been revised accordingly. Please see page 12 for details.

Q42: *L282 hercynite, being a spinnel, would this possibly be the cause of the expansion of the 900C Ilt100 sample observed? Al cations migrate within the Ilt octahedral sheet? (a well established reaction, see works of Drits or MacCarthy and Drits).*

Response: We thank the reviewer for this insightful suggestion. Although hercynite has been reported to form at approximately 950°C following illite decomposition[4], it was not detected

in our samples fired below 1200°C. Therefore, the available evidence does not support hercynite formation as the primary cause of the expansion observed for the Ilt100 sample at 900°C.

In addition, according to Drits, Besson [2], dehydroxylation of Al-rich cis-vacant 2:1 layer silicates occurs through coordination changes and migration of Al cations from former trans-octahedral sites to vacant pentagonal-prismatic sites. The resulting dehydroxylated structure of the original cv 2: 1 layer is similar to that of the former cv 2: 1 layer. We therefore consider that dehydroxylation-induced Al rearrangement within the Ilt octahedral sheet may partly contribute to the expansion. However, the available evidence is insufficient to quantify its contribution or exclude additional effects from particle packing, pore structure, and microcracking. The discussion of the observed expansion can be seen on page 10 of the revised manuscript.

Q43: *L299 should indicate that the total clay mineral content in Fig 3a, d and g is 35%.*

Response: Thank you for this suggestion. The text has been revised accordingly. Please see page 14 for details.

Q44: *L322 ?? mullite is an Al spinel! it is the stable intermediate in the Al-Si system*

Response: We thank the reviewer for this comment. To avoid ambiguity, we have revised the relevant sentence on page 14 as follows: "In contrast, Kln in the starting material promotes the formation of Al-rich or Al-Si spinel-type intermediates prior to substantial mullite crystallization". A detailed clarification of the terminology and crystallographic distinction between the proposed spinel-type intermediate and orthorhombic mullite is provided in our response to Comment Q37.

Q45: *L361 phases*

Response: Thank you for pointing this out. We have corrected "phase" to "phases" on page 16 of the revised manuscript.

Q46: *L370 strongly interdependent with temperature; Fig 3b, c, d, f, g, h, J, k, l. There exist crossovers in WA : as a function of total clay content and ratio of Kln (b); as a function of Ilt content and total clay mineral content (c); as a function of clay mineral content at high Kln content (d) (data for and d are complimentary). All are interrelated to apparent porosity (f, g, h) as well as bulk density (j,k,l).*

Response: Thank you for this comment. We agree that the observed crossovers indicate strong interactions among firing temperature, the Kln/(Kln + Ilt) ratio, and total clay mineral

content, rather than a simple temperature dependence. We have revised the text accordingly on page 16 of the revised manuscript.

Q47: *L439-440 Also greater Kln content*

Response: Thank you for this comment. We agree that increasing the Kln content enhances the CS of bricks fired at 900°C. This effect is discussed in detail on page 18 of the revised manuscript. As the paragraph in question focuses specifically on the influence of total clay mineral content, we have not repeated the discussion here to avoid redundancy.

Q48: *L441-443 Ture only for Ilt rich mixes*

Response: Thank you for this correction. We agree that the positive relationship between total clay mineral content and CS at 1050°C and 1200°C is primarily observed in Ilt-rich formulations. The text on page 19 has been revised to restrict this interpretation accordingly.

Q49: *L445-446 due to lower vitrification of the materials with high Kln*

Response: Thank you. The text has been revised accordingly. Please see page 19 for details.

Q50: *L513-514 prevent vitrification of*

Response: Thank you for this correction. We have changed “the melting” with “vitrification”. Please see page 22 for details.

Q51: *L515 clay mineral content should exceed*

Response: We appreciate the reviewer’s suggestion and have revised the wording accordingly. The amended text is provided on page 22 of the revised manuscript.

Q52: *L520 Figure S26 really deserves a pace in the main manuscript.*

Response: We thank the reviewer for this valuable suggestion. We agree that Figure S26 provides important information supporting the main findings of this study. It has therefore been moved from the Supplementary Information to the main manuscript as Figure 7, and the corresponding figure numbering and in-text citations have been updated accordingly.

Q53: *L525 only the thermal evolution of*

Response: Thank you for this correction. The text has been revised accordingly. Please see page 22 for details.

Q54: *L530-531 systematically evaluating 60*

Response: Thank you for this comment. We have changed "examined" to "evaluating". Please see page 23 for details.

Q55: *L537 Indeed the broader firing*

Response: Thank you for this comment. We have changed "In contrast" to "Indeed". Please see page 23 for details.

Q56: *L538-539 which have been often overlooked previously; Fig 7 A pattern does emerge howevere when looking at Fig 7c.the strongest correlations are for 1200 C, where 4 of the 5 are strong (>|0.75|). A lower temperatures the correlations are lower: Modestly strong (>0.5) to strong for 3 of 5 at 1050 C and 4 of 5 at 900 C. At 1350 C the correlations are weak or modestly strong.*

Response: We thank the reviewer for the suggested correction. During revision, we substantially rewrote this paragraph to incorporate the temperature-dependent pattern identified by the reviewer. Consequently, the original sentence containing "which was usually overlooked before" was removed and the suggested wording is therefore no longer present in the revised manuscript.

We agree that Figure 8c reveals a clear temperature-dependent pattern. The correlations are strongest at 1200°C, where four of the five correlations are strong ($|r| > 0.75$). At 900°C and 1050°C, four and three of the five correlations, respectively, are moderately strong to strong, whereas those at 1350°C are weak or moderately strong. The revised paragraph now clarifies that the relatively low R^2 values obtained from the pooled dataset do not indicate uniformly weak correlations; rather, the relationships vary substantially with firing temperature and may be obscured when data across the entire temperature range are combined. Please see page 23 of the revised manuscript for details.

Q57: *L551 importance of temperature-combined factors*

Response: Thank you for this comment. The text has been revised accordingly. Please see page 24 for details.

Q58: *L554-555. Statement true for temperature-combined relationship only. Individual temperatures show this relationship to be strong at 1200 C, moderately strong at 900 and 1350 Cand weak at 1050 C. Recommend use of a consistent statistical language to define relationships. For example a strong correlation as having an r^2>|0.75|, moderately strong (|0.5|<r^2<|0.75|) and an r^2<|0.5| as weak would seem appropriate.*

Response: Thank you for this clarification. We agree that the original statement applies only to the temperature-combined dataset and does not adequately describe the temperature-specific relationships. We have therefore revised the text on pages 24 and 25 to distinguish the overall statistical significance from the correlation strength at individual firing temperatures. Consistent terminology has also been adopted throughout the manuscript, with correlations classified as strong ($|r| > 0.75$), moderately strong ($0.50 < |r| \leq 0.75$), or weak ($|r| \leq 0.50$). These classification criteria have been added to the caption of Figure 8.

Q59: *L563 Suggestion. is also weakly positive at 0.3*

Response: We appreciate the reviewer's suggestion and have amended the relevant statement to describe the correlation as weakly positive. The revised text is provided on page 25 of the manuscript.

Q60: *L563-566 Sentence needs reworking. Could be taken to imply that mullite contributes more strength which is not what is meant.*

Response: Thank you for pointing out this ambiguity. Our intention was to emphasize that the amorphous/glass phase, rather than mullite, contributes more strongly to CS development. We have reworded the sentence on page 25 to clarify this point.

Q61: *L566 Support this observation,*

Response: Thank you for this suggestion. We have revised the relevant phrase to "support this observation." The corresponding revision can be found on page 25 of the revised manuscript.

Q62: *L569-570 Authors should provide the standard deviations for nano-indentation strength values*

Response: Thank you for this suggestion. We have added the standard deviations for all nanoindentation hardness values and clarified the number of indents used in the analysis. The corresponding results are now reported as mean ± standard deviation on page 25 of the revised manuscript.

Q63: *L576-577 play dominant roles*

Response: Thank you. The phrase has been corrected on age 25 of the revised manuscript.

Q64: *L578 are co-dominant (Figure*

Response: Thank you for this comment. We have revised the term to “co-dominant”. Please see page 25 for details.

Q65: *L579 of inter-related factors*

Response: We appreciate this suggestion and have amended the relevant wording to “inter-related factors.” Details can be seen on page 25 of the revised manuscript.

Q66: *L584 term ground breaking? From a science viewpoint, there is nothing "groundbreaking" of applying the reduction and recombination approach to evaluate particular component contributions. From an industry viewpoint this is probably groundbreaking in that sense.*

Response: Thank you for this valuable comment. We agree that “groundbreaking” may overstate the scientific novelty. We have therefore replaced it with “novel” and revised the sentence on page 26 to emphasize the industrial relevance of applying the KIQF system to evaluate the individual contributions of major mineral components across a broad compositional range.

Q67: *L736-740. Authors should point to evidence that exclusion of intermediate ratios did not compromise. Otherwise why mention that they were excluded from analysis to reduce uncertainty?*

Response: We sincerely thank the reviewer for identifying this important issue, and we apologize for the inaccurate and insufficiently justified wording in the original manuscript. The reviewer is correct that, without quantitative phase data for the 33% and 66% Kln/(Kln+Ilt) compositions, we cannot demonstrate that these intermediate compositions follow a monotonic trend or conclude that their omission does not affect the correlation analysis.

The complete experimental design comprised 60 composition-temperature combinations (three total clay mineral contents × five Kln/(Kln+Ilt) ratios × four firing temperatures). Given the scale of this experimental matrix and the extensive complementary characterization conducted for this study, quantitative phase analysis was performed for a representative

subset of 36 samples comprising the two compositional endmembers and the midpoint (0%, 50%, and 100%) at each total clay mineral content and firing temperature. This sampling strategy covered the full compositional range while keeping the quantitative phase analysis within a manageable scope. However, we acknowledge that this practical consideration does not provide evidence for the behaviour of the unmeasured intermediate compositions, and this limitation should have been stated clearly in the original manuscript.

We have therefore removed the statements that the 33% and 66% compositions were "excluded to reduce uncertainty," that they were expected to follow the same monotonic trend, and that their omission did not compromise the accuracy or comparability of the analysis. The revised text on page 30 now makes clear that these compositions were not quantitatively analysed, restricts the interpretation of the correlations to the measured endmember and midpoint compositions. We appreciate the reviewer's comment, which has helped us present the scope and limitations of this analysis more transparently.

Supporting Information

Q68: *L36 sodium hexametaphosphate is not a surfactant. It is a dispersant. It promotes uniform dispersion by preventing agglomeration (via like charge repulsion)*

Response: Thank you for this correction. We agree that sodium hexametaphosphate is more accurately described as a dispersant rather than a surfactant. The text on page S2 of the revised Supporting Information has been amended accordingly.

Q69: *L47- Purification.*

Clarification needed. The separation of kln from ilt is not clearly described. Were the source materials of ilt and kln separate and different from the brick making source materials? If so this is not clear.

Response: Thank you for pointing out this ambiguity. The Kln- and Ilt-rich source materials were obtained separately and were distinct from the brickmaking raw material. They were purified independently by sedimentation to remove coarse, non-clay impurities; Kln and Ilt were not separated from each other from a common source material. We have revised Supplementary Note 2 on page S3 to clearly describe the origins and independent purification of the two materials.

Q70: *L121-123 Unclear. Explain why? If all the clay is removed from the source - and not lost - in the separation process why would the ratios differ? Is this because the source materials were different for each of the three components – Kln, Ilt and Hp? Again not clear if that was the case.*

Response: Thank you for highlighting this lack of clarity. We have revised Supplementary Note 7 on page S8 of the revised Supporting Information to clarify that the clay-fraction preparation was an analytical procedure independent of, and unrelated to, the purification described in Supplementary Note 2. It was performed solely to identify the clay mineral species in the Hp, Kln, and Ilt samples and to determine whether any expandable clay minerals were present.

We have also clarified that only approximately 1 mL of the uppermost suspension, containing the finest particles and most enriched in clay minerals, was collected for XRD analysis. Because sedimentation fractionated the clay particles by size, not all clay particles entered this uppermost fraction; relatively coarser clay particles remained lower in the suspension or settled to the bottom. Consequently, the phase proportions in the collected fine fraction may differ from those in the corresponding bulk powder (Kln, Ilt, or Hp), and the results were interpreted qualitatively only.

Q71: *Figure S8 3D images show some artifacts due to either beam hardening or shadowing. The upper segments should be removed for the purpose of publication.*

Response: Thank you for pointing this out. In the original 3D rendering, an artificial opaque segment covered the upper surface and obscured the underlying microstructure. To remove this artifact, we first delineated the affected upper segment using the region-of-interest tool and excluded it from the binary mask used for 3D visualization. The 3D image was then re-rendered using the remaining voxels. No gray values or structural features within the retained volume were edited, and the original reconstructed dataset, sample dimensions, scale, and quantitative results remained unchanged. Figure S8 on page S21 has been updated accordingly.

Q72: *Figure S11 based on 11(B), 11(A) could have another separating line along the middle left where Ilt seems concentrated relative to Kln.*

Response: Thank you for the suggestion. An additional separation line has been added to the middle-left region of Figure S11a on page S24 of the revised Supporting Information.

Q73: *L272-273 (Figure S12 caption). Partial vitrification of. Alternatively could state show partially vitrified Kln*

Response: Thank you. The caption of Figure S12 has been revised to “partial vitrification of…”. Please see page S25 of the revised Supporting Information for details.

Q74: *L277 (Figure S13 caption). Needles*

Response: Thank you. This correction has been made to the caption of Figure S13 (page S26 of the revised Supporting Information).

Q75: *L280 (Figure S14 caption) Photos*

Response: We thank the reviewer for pointing this out. The caption of Figure S14 has been corrected accordingly. Please see page S27 of the revised Supporting Information for details.

Q76: *L283 (Figure S15 caption) images …. microscopy showing growth; Figures S16-S25 If 10% corundum added, how does the amount quantify to higher levels? Shouldn't the totals all be normalized to the amount of corundum added as an internal standard? This correction would necessarily reduce the total to < 100% and, since XRD only "sees" the crystalline phases, the residual could be ascribed from difference to 100% to amorphous or glassy phases.*

Response: We thank the reviewer for these comments. The caption of Figure S15 has been corrected accordingly on page S28 of the revised Supporting Information. Regarding the phase quantification using 10 wt% corundum, a detailed explanation of the internal-standard normalization and calculation of the amorphous content is provided in our response to Comment Q26. To avoid repetition, we respectfully refer the reviewer to that response.

Q77: *Figure S26 This is an excellent descriptive chart for decision making – it should be part of the main manuscript.*

Response: Thank you for this helpful suggestion. Figure S26 has now been included in the main manuscript as Figure 7.

Q78: *Figure S28 Error bar estimates for the nanoindentation hardness values would be appropriate.*

Response: Thank you for this helpful suggestion. We clarify that nanoindentation was conducted once for each selected microstructural region; therefore, repeated-test error bars cannot be calculated. However, each test comprised a 7 × 7 array of 49 individual indentations, and each point in Figure S27 represents one indentation measurement. To quantify the variation among these measurements, we calculated the mean hardness and standard deviation of the 49 values for each region. The revised Figure S27 now presents the mean hardness with error bars representing ±1 standard deviation. These error bars describe the local spatial variability in hardness within the tested region, rather than the uncertainty or reproducibility between independently repeated tests. This distinction has also been clarified in the figure caption.

References

1. Wang, S., Gainey, L., Mackinnon, I. D. & Xi, Y. High-and low-defect kaolinite for brick making: Comparisons of technological properties, phase evolution and microstructure. *Construction and Building Materials* **366**, 130250 (2023).
2. Drits, V., Besson, G. & Muller, F. An improved model for structural transformations of heat-treated aluminous dioctahedral 2: 1 layer silicates. *Clays and Clay minerals* **43**, 718-731 (1995).
3. Chakraborty, A. K. *Phase transformation of kaolinite clay*. (Springer, 2014).
4. Jordán, M., Meseguer, S., Pardo, F. & Montero, M. Properties and possible ceramic uses of clays from lignite mine spoils of NW Spain. *Applied Clay Science* **118**, 158-161 (2015).

Comments from Reviewer #3

I have read through all the responses to my (constructive) criticisms, suggestions and corrections and am satisfied that the authors have amended the manuscript or provided rational justification for any decision to not amend. This is a fine manuscript and is acceptable for publication in Nature Communications.

Response: We sincerely thank the reviewer for the positive evaluation of our revised manuscript. We greatly appreciate the reviewer's thoughtful and constructive comments, which have helped us improve the scientific rigor, clarity, and overall quality of the study. We are grateful for the reviewer's time and recommendation for publication.
